# Cellular senescence in the dental pulp and its implications for endodontics: a scoping review

**DOI:** 10.1007/s00784-026-06822-x

**Published:** 2026-03-31

**Authors:** Johnny Carvalho da Silva, Raquel Figuerêdo Ramos, Maria Ester França de Melo, Maria Julia Alves da Silva, Prasanna Neelakantan, Octávio Luiz Franco, Taia Maria Berto Rezende

**Affiliations:** 1 Postgraduate Program in Health Sciences, Faculty of Health Sciences, University of Brasília, Brasília, Brazil; 2 Department of Dentistry, Faculty of Health Sciences, University of Brasília, Darcy Ribeiro Campus, s/n – Asa Norte, DF 70.910-900 Brasília, Brazil; 3 Mike Petryk School of Dentistry, Faculty of Medicine and Dentistry, University of Alberta Edmonton, Edmonton, AB Canada; 4 Women and Children’s Health Research Institute, University of Alberta, Edmonton, AB Canada; 5 Medical Microbiology and Immunology, Faculty of Medicine and Dentistry, University of Alberta Edmonton, Edmonton, AB Canada; 6 Center for Proteomics and Biochemical Analysis, Postgraduate Program in Genomic Sciences and Biotechnology, Catholic University of Brasília, Brasília, Brazil; 7 Postgraduate Program in Biotechnology, S-Inova Biotech, Dom Bosco Catholic University, Campo Grande, Brazil; 8 Postgraduate Program in Dentistry, Faculty of Health Sciences, University of Brasília, Brasília, Brazil

**Keywords:** Cellular senescence, Pulp vitality, Dental materials, Senotherapeutics, Regenerative endodontics

## Abstract

**Introduction:**

Cellular senescence compromises pulp vitality and may negatively affect vital pulp therapy and regenerative endodontics. Exposure to dental materials may accelerate this process, highlighting the need for safer biomaterials and novel therapeutic strategies.

**Objectives:**

To map and analyze the scientific evidence on cellular senescence in dental pulp, focusing on triggers, biological consequences, and implications for endodontic therapies.

**Materials and methods:**

This scoping review was conducted following PRISMA-ScR guidelines (OSF registration: 10.17605/OSF.IO/5ARJH). PubMed, EMBASE, Scopus, Web of Science, SciELO, and Cochrane Library were searched (1999–2026).

**Results:**

From 1,391 records, 94 studies met the inclusion criteria. Most were in vitro (78%) and conducted in Asia (77.7%), mainly using human dental pulp stem cells. Senescence was primarily induced by oxidative stress (hydrogen peroxide), replicative exhaustion, and inflammatory stimuli like lipopolysaccharide. The most consistent markers were SA-β-gal activity and increased p16, p21, and p53 expression. Resin-based monomers, particularly TEGDMA, were associated with senescence induction via oxidative stress and DNA damage. Antioxidants and senolytic compounds reduced senescence markers and improved cell viability and proliferation in vitro.

**Conclusions:**

Oxidative stress and inflammation are key drivers of dental pulp senescence, and resin monomers may contribute to this process. Senescence assessment should be incorporated into biomaterial testing, and senotherapeutics represent potential adjuncts to improve regenerative endodontic outcomes.

**Clinical relevance:**

Identifying senescence-inducing factors may support safer material selection and biologically guided strategies to preserve pulp vitality and improve long-term outcomes.

**Supplementary Information:**

The online version contains supplementary material available at 10.1007/s00784-026-06822-x.

## Introduction

Cellular senescence, once described primarily as a protective mechanism against the proliferation of damaged cells, is now recognized as a context-dependent biological process, contributing to tissue aging and chronic inflammatory conditions when senescent cells persist and accumulate [1]. In dental tissues, the senescent phenotype of the pulp has direct implications for the success of vital pulp therapy and regenerative endodontic procedures, making the understanding of senescence a critical factor in clinical decision-making [2].

Dental pulp is unique amongst tissues impacted by senescence due to its distinct structural and functional characteristics. As a highly vascularized and innervated connective tissue composed of heterogeneous cell populations, dental pulp plays crucial roles in oral health, including sensory, immune, reparative, and regenerative functions. However, with aging and chronic exposure to intrinsic and extrinsic stressors, such as caries, trauma, and restorative materials, the pulp undergoes morphological and functional changes. These alterations result from a multifactorial process involving physiological aging and sustained inflammatory stimuli, driven by the accumulation of senescent cells, compromising not only the vitality but also the effectiveness of conservative and regenerative endodontic therapies [3].

**Fig. 1:**
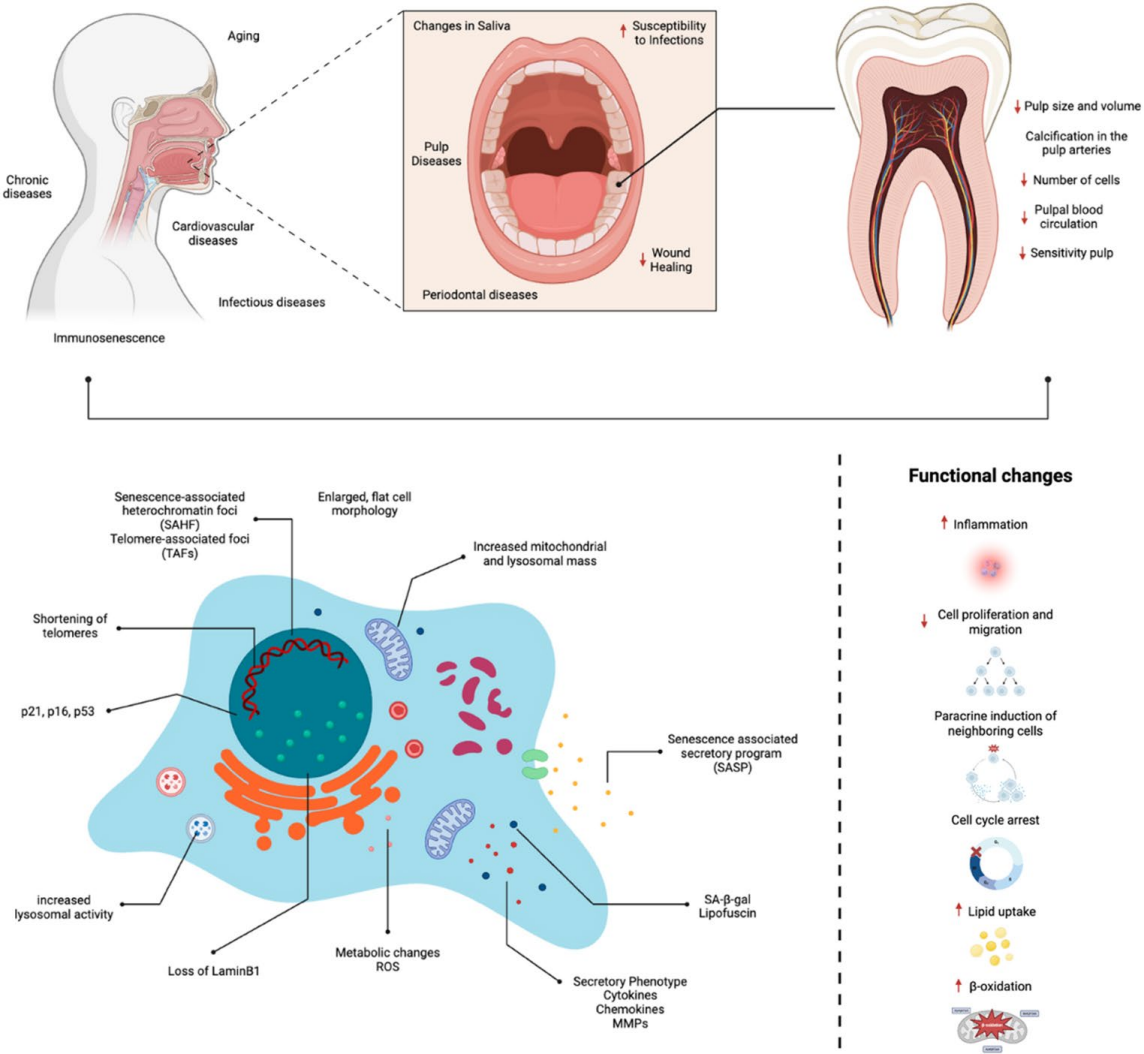
Schematic figure of the systemic repercussions of cellular senescence and its relationships with oral cavity and the dental pulp. Created by BioRender.com

Recent research leverages powerful molecular biology tools has advanced the understanding of the molecular mechanisms underlying pulp senescence. Among the characteristic markers, notable ones include telomere shortening, increased expression of senescence-associated genes such as p21 and p16, and the manifestation of a senescence-associated secretory phenotype (SASP) (Fig. 1) [1, 4]. The SASP is characterized by the secretion of pro-inflammatory cytokines, including mediators such as interleukin-6 (IL-6) and interleukin-8 (IL-8), and matrix-degrading enzymes, contributing to heightened local inflammation, accelerated tissue degeneration, and impaired pulp functionality [2]. Importantly, cellular senescence should be conceptually distinguished from general cytotoxicity or transient inflammatory responses. While cytotoxic effects typically result in cell death or acute cellular damage, senescence represents a stable state of cell-cycle arrest, accompanied by specific molecular markers and the secretion of SASP factors [2, 3].

Despite increasing recognition of cellular senescence as a determinant of pulp aging and treatment outcomes, the existing evidence remains fragmented across a wide variety of study designs, including in vitro models, animal studies, clinical investigations, and narrative reviews. This substantial variability in experimental models, methodologies, and outcome measures constrains quantitative synthesis, particularly meta-analytical approaches, in the context of this emerging field. According to the methodological guidance of the Joanna Briggs Institute and the PRISMA-ScR extension, scoping reviews are particularly suitable for mapping the breadth of available evidence, identifying research gaps, and clarifying key concepts in emerging fields. In this context, a scoping review is an appropriate approach to provide an overview of how cellular senescence in dental pulp has been investigated, to highlight potential clinical implications for vital pulp therapy and regenerative endodontics, and to inform future research directions. Therefore, this review aims to map and descriptively analyze the available scientific literature on cellular senescence in dental pulp, with emphasis on its triggers, and biological consequences.

## Materials and methods

### Eligibility criteria

This scoping review was conducted to address the following research question: “How has cellular senescence in dental pulp been investigated in the scientific literature, and what are its main triggers and biological consequences?” A scoping review was chosen instead of a systematic review or meta-analysis because the available literature is highly heterogeneous in terms of populations (human, animal, and in vitro), interventions (various senescence-inducing stimuli), outcomes (molecular markers, morphological features, functional assays), and study designs (in vitro, in vivo, observational), making quantitative synthesis unfeasible.

Sources of evidence considered eligible included experimental studies (in vitro and in vivo), clinical investigations, and observational studies that assessed cellular senescence in dental pulp tissue or cells derived from dental pulp. Studies addressing senescence induced by physiological aging, replicative exhaustion, inflammatory stimuli, oxidative stress, or exposure to stressors related to the dental environment were included.

For eligibility purposes, studies were required to directly investigate cellular senescence in dental pulp cells or tissue and confirm the senescent state using at least one widely recognized marker, such as senescence-associated β-galactosidase activity, altered expression of senescence-related genes or proteins (e.g., p16, p21, and p53), telomere shortening, activation of the DNA damage response, or features associated with the senescence-associated secretory phenotype.

Only studies published between 1999 and 2026 were included. Studies published before 1999 were excluded because the literature on dental pulp senescence began to clearly distinguish cellular senescence from general aging only after this period. Review articles, systematic reviews, and narrative reviews were excluded, as the objective of this scoping review was to map primary evidence on cellular senescence in dental pulp.

Studies were excluded if they did not focus on dental pulp or pulp-derived cells, did not include any assessment or confirmation of cellular senescence, or investigated exclusively genetically modified, immortalized, or non-dental cell lines without relevance to pulp biology.

### Information sources and search strategy

This review was conducted in accordance with PRISMA 2020 guidelines and registered in Open Science Framework (DOI: 10.17605/OSF.IO/5ARJH). Searches were performed in PubMed (MEDLINE), EMBASE, Scielo, Web of Science Core Collection, Scopus, and the Cochrane Library. Full search strategies for all databases are provided in Supplementary Table 1.

### Selection process

Duplicate records were removed using EndNote and Rayyan^®^. Articles were selected by three independent researchers (J.C.S., M.E.F.M., and R.F.R.) through review of titles, abstracts, and full texts when necessary. Disagreements were resolved by consensus with a fourth researcher (T.M.B.R.). The study selection process is summarized using a PRISMA flow diagram.

### Data extraction from selected studies

Data extraction was conducted independently by three reviewers (J.C.S., M.E.F.M., and R.F.R.) using a standardized form. To support the process, an artificial intelligence tool (Deepseek) was employed to assist in capturing key study characteristics, including study design, cell or tissue type, main objectives, methods of senescence induction, primary senescence markers assessed, key findings, implications for endodontics, and study limitations. The role of artificial intelligence was limited to assisting in the preliminary organization and capture of information, and it was not used to make decisions regarding study selection or final data extraction. All AI-assisted extractions were subsequently verified manually for accuracy. Bibliometric data were also collected for each article, including publication year, study type, geographic distribution, authors, affiliated institutions, and keywords. Study types were categorized as in vitro, in vivo, in vitro & in vivo, in vitro & ex vivo, or observational. Extracted data were organized and managed using Microsoft Excel 2024 (Microsoft, Redmond, WA).

### Risk of bias assessment

Two reviewers (J.C.S. and R.F.R.) independently assessed the risk of bias using a critical appraisal instrument adapted from the Petriccs guideline for in vitro studies [5] and the SYRCLE tool for in vivo studies [6]. Discrepancies between reviewers were resolved through discussion, with a third reviewer (M.E.F.M.) consulted when consensus could not be reached. Each item was scored as “yes,” “no,” or “unclear.” Studies were classified according to the proportion of “yes” responses as high risk (< 50%), moderate risk (50–69%), or low risk (≥ 70%).

### Data analysis

Bibliometric maps were generated using Visualization of Similarities Viewer (VOSviewer) software (version 1.6.20, Netherlands), and additional graphs were created with GraphPad Prism 10.

## Results

### Selection of the studies

**Fig. 2:**
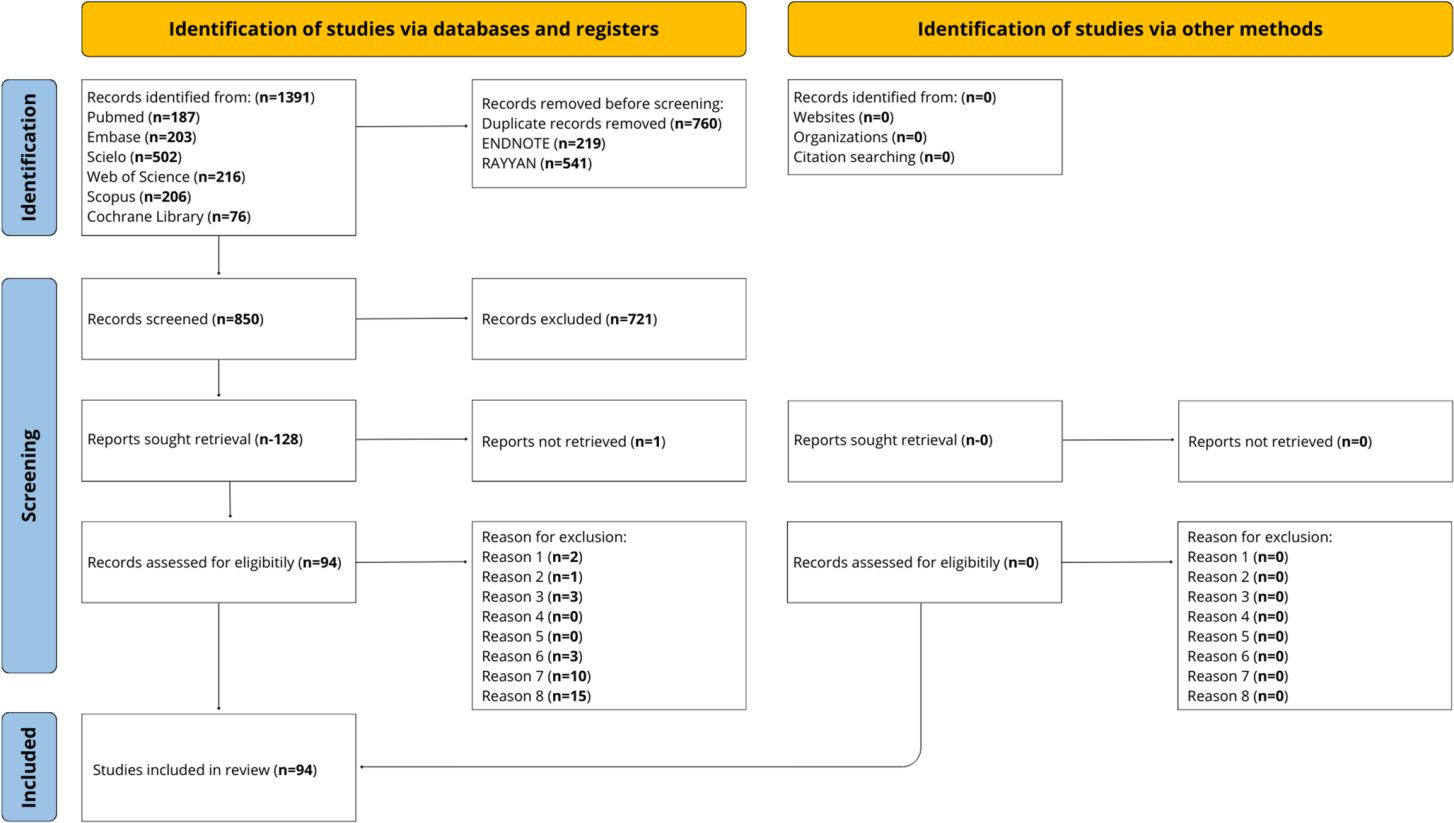
Flow diagram of literature search and selection criteria adapted from PRISMA 2020

After a database search, 1,391 records were initially identified. After removing duplicates and records marked as ineligible by automation tools, 850 studies remained for the screening phase. Upon examining the titles and abstracts, 721 studies were excluded for not meeting the inclusion criteria. Thus, 128 studies were selected for a full-text eligibility assessment. Of these, 34 reports were subsequently excluded, each for specific reasons (Supplementary Table 2). Finally, 94 studies published between 1999 and 2026 were included in this review (Fig. 2; Supplementary Table 3).

### Risk of bias in studies

The majority of in vitro studies were rated as having a low overall risk of bias according to the PETRICCS-based assessment, exhibiting high completeness in reporting, and none were categorized as high risk (Supplementary Fig. 1). Frequently observed shortcomings included inadequate details on blinding, randomization, and the number of replicates. In vivo studies evaluated with SYRCLE’s tool displayed methodological quality ranging from low to moderate, often showing deficits in randomization, blinding, and protocol registration, which could compromise both internal validity and the translational applicability of the findings (Supplementary Fig. 2).

### General overview of studies and bibliometric analysis

**Fig. 3:**
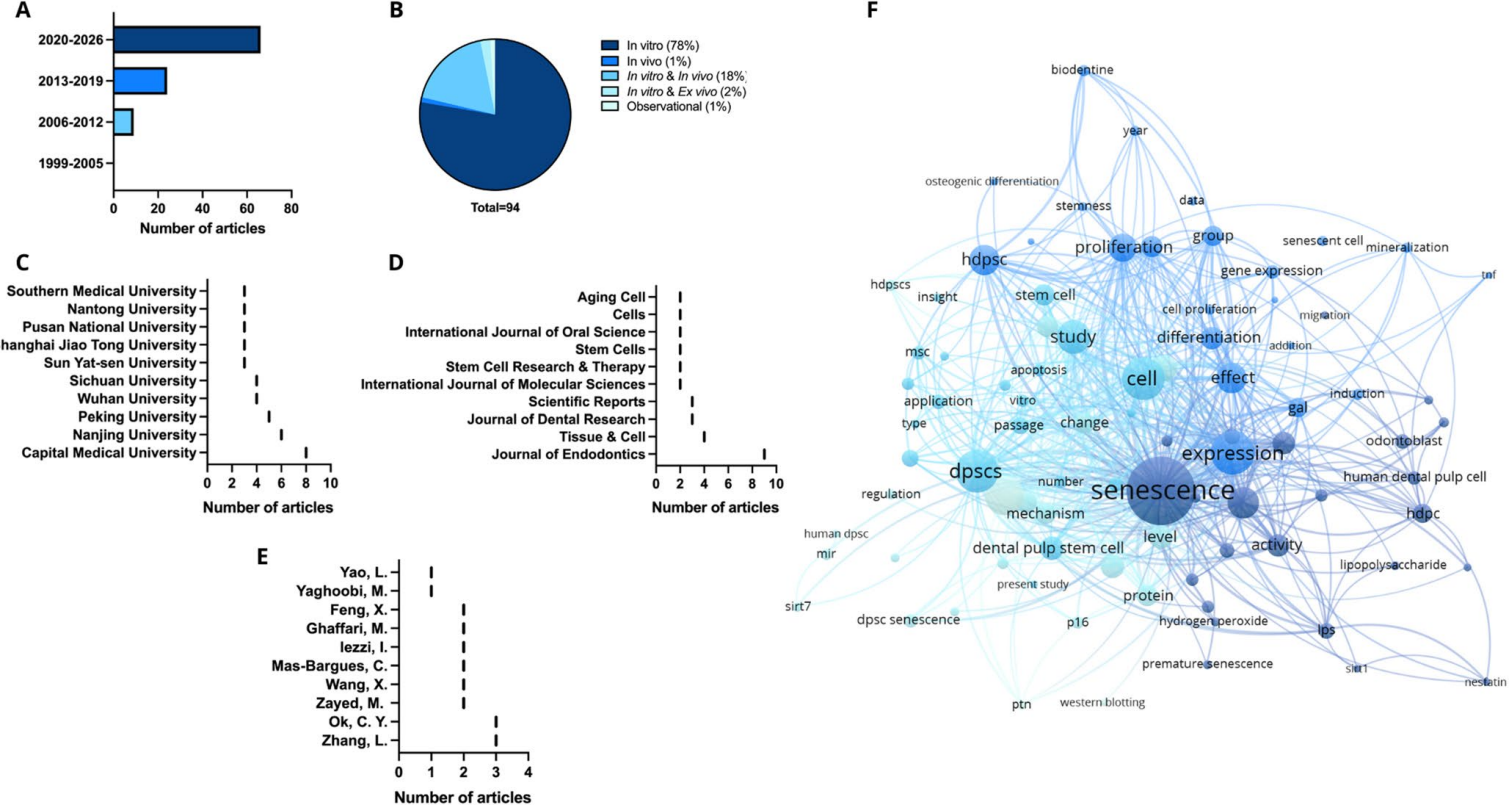
Overview of scientific production on cellular senescence in dental pulp (1999–2026). (A) Temporal distribution of published articles in seven-year intervals, illustrating the increasing research interest over time. (B) Study design classification of the 94 analyzed studies, showing the proportion (%) of in vitro, in vivo, combined, and observational approaches. (C) Top 10 most productive institutions in dental pulp senescence research. (D) Top 10 journals publishing on the topic. (E) Top 10 authors with the highest number of publications related to dental pulp cell senescence. (F) Keyword co-occurrence network, highlighting major research topics and their interconnections within the context of pulp aging. Note: Data for 2026 are partial, corresponding to the beginning of the year

A total of 94 studies on dental pulp senescence were included, with a marked increase in the number of publications from 2020 onwards (Fig. 3A). Regarding study design, most were in vitro (*n* = 73; 78%), followed by studies combining in vitro and ex vivo approaches (*n* = 17; 18%). No clinical studies on this topic were identified (Fig. 3B).

Most studies originated from Asia (*n* = 73; 77.7%), with the main contributing countries being China (*n* = 54), South Korea (*n* = 5), and Japan (*n* = 4). Europe contributed 12 studies (12.8%), primarily from Italy (*n* = 3), the United Kingdom (*n* = 2), Spain (*n* = 2), and Germany (*n* = 2). North America accounted for four studies (United States: *n* = 2; Canada: *n* = 2). South America and Africa had limited representation, with studies from Brazil (*n* = 2), Chile (*n* = 1), and Egypt (*n* = 2) (Supplementary Fig. 3).

The leading institutions, identified based on the total number of publications, were Capital Medical University (*n* = 8) and Nanjing University (*n* = 6) (Fig. 3C). Among the 84 listed authors, 76 published only one study, while 8 authors contributed to two or more articles. Zhang (*n* = 3) and Ok (*n* = 3) were the most prolific authors (Fig. 3D). Publications were concentrated in a limited number of journals, with Journal of Endodontics being the most frequent (*n* = 9) (Fig. 3E).

A total of 87 keywords were identified and standardized regarding singular/plural forms and semantically equivalent terms. The frequency of each term was determined by the absolute count of occurrences across all studies. For the co-occurrence analysis, a minimum threshold of four occurrences per keyword was applied. This criterion was adopted to reduce analytical noise from infrequent terms and to robustly highlight the central concepts and their interrelationships in the field.

The most frequent terms were “senescence,” “cell,” and “expression” (Fig. 3F). The co-occurrence network demonstrated the centrality of these concepts, while the most cited articles focused on immunosenescence and senescence-related genes (Supplementary Table 4).

### Study characteristics

This scoping review included a total of 94 studies, the main characteristics of which are summarized in Table 1 and detailed in Supplementary Table 5. The most frequently used cellular models were human dental pulp stem cells (hDPSCs), present in most studies, followed by heterogeneous cultures of pulp cells (HDPCs) and cells from other niches, such as those derived from deciduous tooth pulp (SHED) (Supplementary Table 5). Senescence was induced through a variety of methods, reflecting the multiple etiologies of the phenomenon (Supplementary Table 5). The most common models included replicative senescence induced by prolonged serial passaging in vitro; oxidative stress-induced senescence, predominantly through exposure to hydrogen peroxide (H₂O₂); and acute chemical induction. Furthermore, studies employed natural aging models, comparing cells from donors of different age groups, or inflammatory models, using lipopolysaccharide (LPS) to mimic a chronic pulpitis microenvironment. The senescence markers assessed were consistent across studies, with senescence-associated β-galactosidase (SA-β-gal) activity being the most frequently reported morphological marker, accompanied by the analysis of cyclin-dependent kinase inhibitor expression, such as p16INK4a, p21CIP1/WAF1, and p53 (Supplementary Table 5). The interventions investigated, as categorized in Table 1, range from antioxidant strategies and modulation of specific signaling pathways to the use of bioactive materials and emerging approaches such as peptide therapy and senolytics. The main clinical implications in Endodontics, extracted from the findings and synthesized in Table 1, focus on the potential to improve the regenerative response in inflamed or aged pulp, optimize cell expansion protocols, and develop targeted therapeutic strategies. In summary, the body of evidence presents a robust and diverse profile that underpins the thematic understanding summarized in Table 1 and charts a promising path for its translation into regenerative clinical applications.

**Table 1:** Synthesis of knowledge on cellular senescence applied to Endodontics

Thematic Category	Mechanisms/Pathways Involved	Interventions/Strategies Studied	Main Effect	References (from supplementary table I)	Clinical Implications in Endodontics
Oxidative Stress-Induced Senescence	H₂O₂, ROS, p53/p21, SIRT1, Nrf2/HO-1	Antioxidants (melatonin, LMWP-SOD1, resveratrol), PTN, Cpne7, Apelin-12, visfatin inhibition	Reduction of senescence markers, restoration of cell function	[5, 7, 16, 18, 20, 23, 24, 26–28, 32, 33, 35–42, 44–94]	Improves regenerative response in pulpitis; protects against oxidative damage during endodontic procedures.
Replicative & Culture-Induced Senescence	Prolonged passaging, telomeres, p16/p21, autophagy, SIRT7, METTL3, m6A	Culture conditions (hypoxia, 3D, substrate stiffness), epigenetic modulation, senolytics, serine/folate metabolism	Delays senescence, maintains stemness, improves cell expansion yield	[1, 4, 10–15, 17, 19, 21, 22, 25, 29–31, 34, 43–94]	Optimizes ex vivo cell expansion protocols for regenerative therapies; informs selection of senescence-resistant cell sources.
Inflammatory-Driven Senescence	LPS, TLR4/NF-κB, TNF-α, SASP, IL-6, IL-8, p16INK4a	TLR4 inhibitors, inflammatory pathway modulators, antioxidants, Nesfatin-1, Apelin-12	Reduces inflammation-induced senescence, improves reparative capacity	[8, 9, 16, 20, 23, 24, 26–28, 32, 33, 35–42, 44–94]	Provides strategies to manage chronic pulpitis and protect pulp from inflammatory damage, potentially improving vital pulp therapy outcomes.
Senescence & Osteo/Odontogenic Differentiation	Wnt/β-catenin, BMP, RUNX2, DSPP, OCN, SIRT6, ILK/AKT/mTOR	Biodentine, Wnt modulators, SIRT activators, mTOR inhibitors, peptide therapy (Cpne7)	Restores differentiation potential, promotes dentin repair	[1, 6, 7, 11–16, 18, 20, 23, 24, 26–28, 32, 33, 35–42, 44–94]	Guides development of bioactive materials and therapies to enhance dentin regeneration in aged or compromised pulp.
Biomarkers & Cellular Heterogeneity in Senescence	IGFBP7, SASP, miRNAs (miR-152, miR-433), proteomics, scRNA-seq	Identification of resistant subpopulations (e.g., MCAM + JAG+ PDGFRA-), cell purification (STRO-1+), secretome analysis	Identifies cells with higher regenerative potential and lower senescence	[2, 3, 8–15, 17, 19, 21, 22, 25, 29–31, 34, 43–94]	Enables development of diagnostic tools and personalized cell selection strategies for regenerative endodontics.
Systemic & Environmental Factors in Pulp Senescence	Hyperglycemia, uremic toxins (p-Cresol), radiation, D-galactose, obesity	Metabolic control, antioxidants, detoxification pathway modulation	Mitigates senescence acceleration from systemic comorbidities	[8, 9, 16, 20, 23, 24, 26–28, 32, 33, 35–42, 44–94]	Highlights the need for an integrated treatment approach, considering systemic patient health in endodontic prognosis and planning.

### Dental products associated with cellular senescence

Numerous dental materials were associated with processes that can lead to cellular senescence in the dentin-pulp complex, primarily via oxidative stress, DNA damage, and chronic inflammation (Table 2). All studies included in Table 2 were conducted in vitro, and no animal studies are reported, as extrapolation from animal models to human pulp tissue may be problematic. While many studies report general cytotoxicity or pulp irritation, findings not synonymous with senescence, several materials demonstrate mechanisms or specific responses, such as irreversible cell cycle arrest and activation of senescence pathways, that align with a senescent phenotype.

**Table 2:** Dental products that can cause pulp senescence

Product Name	Main Components	Proposed Mechanism for Senescence Induction	Type of Senescence/Identified Markers	Potential Biological/Clinical Consequences	Notes (Beneficial vs. Detrimental Context)	Reference
Chlorhexidine	Chlorhexidine digluconate	(1) Endoplasmic reticulum stress → unfolded protein response. (2) Membrane disruption → oxidative stress & DNA damage.	Likely Stress-Induced Premature Senescence (SIPS). Markers not directly assessed (inferred from mechanism).	(1) Reduced reparative capacity. (2) Chronic inflammation via SASP if senescent cells accumulate.	Antimicrobial effect beneficial; cytotoxicity and potential senescence induction are detrimental to tissue repair. Requires careful concentration/exposure control.	[7]
Calcium hydroxide cement	Calcium tungstate; zinc oxide; disalicylate ester; calcium phosphate	(1) Chemical injury via high pH (~ 12.5). (2) Moderate oxidative stress (↑ ROS).	Not directly assessed. Severe cytotoxicity and cell cycle delay observed. Senescence markers not evaluated.	(1) Depletion of progenitor cells. (2) Persistent inflammatory niche if senescence occurs.	Paradox: Significant in vitro cytotoxicity vs. clinical success. Detrimental cytotoxic effect may be balanced by beneficial bio-stimulatory/sterilizing effect triggering reparative response.	[8]
Dental Amalgam	Mercury (vapor, Hg²⁺ ions)	(1) Oxidative stress & glutathione depletion. (2) DNA damage & activation of cell death pathways. (3) Neurotoxicity via zinc release.	Stress-Induced Premature Senescence (SIPS) inferred. Markers not directly measured (ROS, DNA damage, p53/p21 inferred).	Cytotoxicity, reduced viability, persistent pulp inflammation. Potential systemic toxicity at high exposure.	Predominantly detrimental in vitro. Clinical relevance at standard exposure is considered low by regulatory agencies; risk-benefit favors use in many situations.	[9–11]
Riva Light Cure	HEMA; Camphorquinone	(1) Transdentinal diffusion of monomers. (2) Induction of oxidative stress & DNA damage. (3) Low-grade persistent inflammatory stimulus.	SIPS inferred from mechanism/histopathology. Direct senescence markers not measured.	Short-term: Mild pulp inflammation. Long-term: Possible persistent irritation, impaired optimal repair.	Dual context: Initial detrimental pulp damage, but inflammatory response diminishes and reparative dentin forms. Outcome is positive (vital pulp, dentin bridge).	[12]
Riva Self Cure	Polyacrylic acid, Tartaric acid, Fluoroaluminosilicate glass	(1) Initial inflammatory stimulus from conditioning. (2) Persistent low-grade chemical irritation.	Tissue response consistent with early senescence phenotype. Classic senescence markers not assayed.	Short-term: Transient inflammation. Long-term: Risk of chronic low-grade inflammation exhausting reparative capacity.	Detrimental senescence not strongly evidenced. Response appears beneficial/adaptive, leading to tissue repair (dentin bridge).	[12]
Vitrebond	HEMA, Dichlorodiphenyliodonium chloride	(1) Monomer-induced oxidative stress & DNA damage. (2) Chronic foreign-body reaction.	Tissue response consistent with senescent outcomes. Molecular markers not assayed.	High risk of direct capping failure: persistent inflammation, lack of dentin bridge. Deep lining: chronic cytotoxicity & inflammation.	Clearly detrimental. Lacks reparative stimulus; promotes chronic pathology. Not recommended for direct pulp contact.	[13]
Vitremer	HEMA, TEGDMA, Polyacrylic acid, Acrylic acid copolymer, Diphenyliodonium hexafluorophosphate	(1) Leaching of unpolymerized monomers (HEMA, TEGDMA) causing oxidative stress & DNA damage. (2) Cytotoxicity of acidic components.	Severe cytotoxic effects (precursors to senescence). Classic markers not assayed.	Direct contact: severe damage, necrosis. Indirect contact: senescence induction, depleted regeneration, chronic inflammation.	Clearly detrimental. High cytotoxicity, lacks reparative properties. Should not contact pulp tissue.	[13]
Phosphoric Acid (32%)	Phosphoric acid	Acid etching increases dentin permeability, facilitating toxin diffusion and causing persistent inflammation/oxidative stress.	Stress-induced senescence plausible. Specific markers not assessed.	Persistent pulp inflammation, tissue disorganization, failure of reparative dentin formation in direct capping.	Context-dependent. Essential for adhesion but detrimental on deep dentin without protective liner.	[14–16]
Phosphoric acid (35%)	Phosphoric acid	Acid etching of deep dentin facilitates monomer diffusion, causing persistent inflammatory response and cellular stress.	Senescence plausible from chronic injury. Markers not assessed.	Persistent inflammation, odontoblast layer disruption, impaired reparative dentinogenesis.	Context-dependent. Beneficial for adhesion; detrimental on deep dentin without liner.	[17]
Adper™ Scotchbond™ 1 XT	Phosphoric acid, Bis-GMA, HEMA	(1) Monomer-induced oxidative stress & DNA damage. (2) Cell cycle disruption (G0/G1 arrest). (3) Diffusional toxicity via opened tubules.	Stress-Induced Premature Senescence (SIPS). Cell cycle arrest, reduced metabolism, apoptosis noted. Canonical markers not assayed.	Impaired pulp repair, chronic low-grade inflammation via SASP, reduced long-term vitality.	Mainly detrimental, especially in deep cavities. Proper handling/polymerization are mitigating factors.	[18–20]
Clearfil™ SE Bond 2	10-MDP, HEMA, Dimethacrylates, Photoinitiators	(1) Acidic/monomer-induced oxidative stress & DNA damage. (2) Potent cell cycle arrest. (3) Activation of apoptotic pathways.	SIPS/Therapy-Induced Senescence. Profound cell cycle arrest, reduced viability, apoptosis. SA-β-gal not assayed.	Severe impairment of pulp repair, chronic inflammation via SASP, high necrosis risk in deep cavities.	Predominantly detrimental, undermining pulp reparative capacity. Cytotoxicity may be higher than etch-and-rinse systems in extracts.	[18, 20, 21]
Scotchbond™ Universal	10-MDP, Bis-GMA, HEMA, Ethanol, Water	(1) Monomer leachability & diffusion. (2) Cytotoxicity & cell cycle disruption (G0/G1 trend). (3) Moderate inflammatory potential.	Senescence phenotype inferred from cytotoxicity data (reduced proliferation, cell cycle alteration, apoptosis).	Pulp tissue injury, delayed healing, potential post-operative sensitivity or chronic pulpitis.	Predominantly detrimental but lower cytotoxicity vs. strong self-etch adhesives. No evidence of beneficial role.	[18, 21]
Scotchbond MP	Bis-GMA, HEMA	(1) Direct cytotoxicity of unpolymerized monomers. (2) Persistent foreign-body reaction. (3) Inhibition of pulp repair.	Inferred from histopathology: apoptosis induction, chronic inflammation, disrupted tissue architecture.	Pulp necrosis/chronic pulpitis, failed direct capping, subcutaneous granuloma formation.	Exclusively detrimental. Not for direct pulp capping; requires adequate dentin thickness as barrier.	[13]
Filtek Supremo	UDMA, Bis-GMA, TEGDMA, Bis-EMA, Zirconium, Silica	(1) Persistent monomer leaching. (2) Induction of oxidative stress & inflammation. (3) Direct cytotoxicity & genotoxicity.	Senescence pathways inferred: oxidative stress, chronic inflammation, DNA damage, cell cycle disruption.	Pulp tissue degeneration via chronic low-grade exposure, systemic allergic risks, compromised restoration longevity.	Predominantly detrimental. Prolonging curing time reduces monomer leaching and potential risk.	[22]
Filtek Flow	Bis-GMA, UDMA, TEGDMA, Bis-EMA	Leaching of residual monomers (TEGDMA, Bis-GMA) inducing cytotoxicity, genotoxicity, pro-inflammatory responses.	Stress-induced senescence implied via DNA damage/SASP mechanisms. Markers not directly studied.	Compromised pulp cell viability/function, chronic inflammation, impaired repair, reduced regeneration.	Adverse reaction to cytotoxic/genotoxic monomers; represents loss of homeostasis and repair potential.	[22]
Bulk Fill	UDMA, TEGDMA, HEMA	(1) TEGDMA-induced oxidative stress & DNA damage (8-oxoG). (2) DNA damage response (ATM) activation. (3) Sustained stress kinase (p38, ERK1/2) signaling.	Stress-Induced Premature Senescence (SIPS)/DNA Damage-Induced Senescence. Markers: 8-oxoG, p-ATM, cell cycle arrest, GSH depletion, p-p38/p-ERK.	Senescence of hDPSCs depletes regenerative pool, causes chronic inflammation via SASP, long-term pulp degeneration.	Detrimental. Maladaptive response impairing pulp regenerative potential.	[23–26]
X-trafil	Bis-GMA, UDMA, TEGDMA	Monomer leaching (esp. TEGDMA) → oxidative stress (ROS, GSH depletion) → oxidative DNA damage → sustained DDR (ATM) & stress kinase (p38, ERK1/2) activation.	SIPS driven by oxidative stress/DNA damage. Markers: ROS/GSH, 8-oxoG, p-ATM, p-p38/p-ERK, cell cycle arrest, reduced hDPSC viability.	hDPSC senescence compromises pulp regeneration, leading to impaired reparative dentin, chronic pulpitis, pulp necrosis risk.	Predominantly detrimental. Senescence in hDPSCs depletes regenerative pool, accelerating functional decline.	[23–26]
Carbamide Peroxide & Hydrogen Peroxide Vital Bleaching	H₂O₂ released from carbamide or hydrogen peroxide gels	H₂O₂ diffusion into pulp → potent pro-oxidant → ROS generation → oxidative stress → mitochondrial dysfunction & DNA damage.	SIPS driven by oxidative stress. Markers: mitochondrial inhibition, reduced viability; oxidative DNA damage & cell cycle arrest inferred.	Acute: cytotoxicity, inflammation, vascular necrosis. Chronic: senescence of progenitor cells impairs reparative capacity, increases pulp necrosis risk.	Predominantly detrimental in this context. Benefit is aesthetic. Risk elevated with thin dentin, patent tubules, prolonged exposure. Requires careful case selection and protective barriers.	[27]

Notably, resin-based materials, including adhesives, resin-modified glass ionomers, and composites, leach monomers capable of inducing oxidative stress, DNA damage, and direct cell cycle arrest. For TEGDMA, specific evidence confirms the triggering of senescence in dental pulp stem cells. Other agents, such as chlorhexidine, hydrogen peroxide whiteners, and phosphoric acid etchants applied to deep dentin, create sustained cellular stress or inflammatory microenvironments recognized as drivers of senescence.

## Discussion

The scoping review conducted highlights the progressive consolidation of cellular senescence as a relevant topic in dental research, with direct implications for contemporary endodontics. By integrating bibliometric analyses and thematic mapping, this study provides a comprehensive and up-to-date overview of scientific production, enabling the identification of research trends, geographic concentration of studies, and knowledge gaps that have not yet been systematically explored in the literature.

The marked growth in publications from 2020 onward, predominantly originating from Asian countries and mainly focused on in vitro models, which represented approximately 78% of the included studies, suggests that the field is still in a phase of biological and molecular characterization of the phenomenon. This approach has been essential for consolidating markers and pathways associated with pulp senescence, including activation of the p53/p21 axis, increased SA-β-galactosidase activity, and modulation of the senescence-associated secretory phenotype [28–31]. However, it also highlights a relevant gap, characterized by the scarcity of clinical studies directly correlating the burden of senescent cells with therapeutic outcomes in endodontics, such as the success of vital pulp therapy or regenerative procedures, representing one of the main challenges for the clinical translation of knowledge generated in experimental models [32, 33].

 The analysis of the included studies also demonstrated that pulp senescence is not restricted to physiological aging and may be induced by stressors frequently present in clinical practice, such as chronic inflammation, oxidative stress, and exposure to bioactive components released from dental materials [29; 34; 12; 13). In this context, the senescence-associated secretory phenotype, characterized by the release of proinflammatory cytokines and metalloproteinases, contributes to the formation of a microenvironment unfavorable to tissue homeostasis, impairing odontoblastic function, the proliferative and migratory capacity of dental pulp stem cells, and extracellular matrix integrity [34–36]. This mechanism provides a plausible biological basis for the observed clinical implications, since pulps with a higher burden of senescent cells tend to show reduced intrinsic regenerative potential, which may negatively impact dentin bridge formation in pulp capping procedures, local immune response, and the performance of regenerative strategies, including revascularization and cell-based therapies [31, 37, 38].

The synthesis of evidence also suggests that materials capable of releasing residual monomers, such as HEMA, TEGDMA, and Bis-GMA, or that induce pronounced oxidative stress, such as high concentrations of hydrogen peroxide, show greater senogenic potential [12, 13, 22, 27], whereas calcium silicate-based cements tend to demonstrate a more favorable biological profile, with lower cytotoxicity and higher bioactivity [37, 39]. Likewise, findings related to the functional impairment of senescent pulp stem cells reinforce the need for strict control of variables such as donor age, cell passage number, and culture conditions in protocols involving cell therapies or the use of platelet-rich plasma [31, 38, 40].

In parallel, the mapping of experimental interventions indicates promising perspectives for the development of senotherapeutic strategies, including the potential local use of senolytic agents for selective elimination of senescent cells [41] and senomorphic or antioxidant agents for modulation of the SASP phenotype and oxidative stress [41–43], although these approaches still depend on robust preclinical validation in pulp injury models before any clinical application [32, 41].

Despite the relevance of the findings, this study presents limitations inherent to the methodological design and to the current stage of knowledge in the field. The high heterogeneity of the included studies, involving different cellular models, senescence induction methods, and analyzed markers, prevented the performance of quantitative syntheses or meta-analysis, restricting interpretation to a descriptive and qualitative approach. In addition, the geographic predominance of Asian studies may limit the global representativeness of the available data, and the scarcity of clinical investigations prevents direct confirmation of the discussed implications in patient populations. Finally, although the use of artificial intelligence-based tools to support data extraction was followed by manual verification, this strategy still represents a potential methodological limitation, which is likely to be progressively overcome with the advancement and validation of these technologies in future scientific reviews. Another limitation is that studies published before 1999 were not included, which may have excluded early insights into dental pulp senescence and could affect the comprehensiveness of the review.

## Conclusion

This scoping review achieved its objective by mapping and critically analyzing the available literature on cellular senescence in dental pulp, enabling the consolidation of the current state of knowledge regarding its triggers, molecular mechanisms, and biological consequences. Overall, the evidence indicates that senescence represents a central component of pulp pathophysiology, being modulated by both chronological aging and environmental and iatrogenic factors. Furthermore, the senescence-associated secretory phenotype (SASP) emerges as a key element in maintaining a pro-inflammatory microenvironment, potentially impairing the reparative and regenerative processes of pulp tissue. From a clinical perspective, three main implications stand out for Endodontics. First, it becomes relevant to consider the senogenic potential when selecting materials and protocols used in conservative therapies. Second, senescence may represent an underlying biological factor in cases of vital pulp therapy failure or unsatisfactory regenerative responses. Third, a new therapeutic field based on senotherapeutic agents is emerging, which may eventually be incorporated into clinical strategies with the potential to enhance the predictability and success of regenerative Endodontics. Finally, progress in this field will depend on strengthening the translational bridge between basic research and clinical application. In this context, longitudinal clinical studies are needed to validate reliable senescence biomarkers in human pulp tissue, as well as robust investigations to assess the safety, efficacy, and clinical applicability of senomodulatory interventions in specific endodontic scenarios.

## Supplementary Information

Below is the link to the electronic supplementary material.

Supplementary Material 1 (DOCX 16.2 KB)

Supplementary Material 2 (DOCX 28.9 KB)

Supplementary Material 3 (DOCX 31.2 KB)

Supplementary Material 4 (DOCX 22.3 KB)

Supplementary Material 5 (DOCX 81.5 KB)

Supplementary figure 4 (PNG 70.9 KB)

High Resolution Image (TIF 6.38 MB)

Supplementary figure 5 (PNG 144 KB)

High Resolution Image (TIF 57.6 MB)

Supplementary figure 6 (PNG 585 KB)

High Resolution Image (TIF 5.95 MB)

## Data Availability

No datasets were generated or analysed during the current study.
