# Supplementary material for: Cellular senescence in the dental pulp and its implications for endodontics: a scoping review
Source: Clin Oral Investig. 2026 Mar 31;30(4):161. doi: 10.1007/s00784-026-06822-x (PMC13035753; doi:10.1007/s00784-026-06822-x)
Supplement: Supplementary file 1 — Supplementary Material 1 (DOCX 16.2 KB) [file 784_2026_6822_MOESM1_ESM.docx]

| Search | Query | Items Found |
| --- | --- | --- |
| Pubmed | (("Cellular Senescence"[Mesh] OR "cellular senescence"[tiab] OR "senescent cell*"[tiab] OR senescence[tiab] OR "pulp senescence"[tiab] OR "senescent pulp"[tiab]) AND ("Dental Pulp"[Mesh] OR "dental pulp"[tiab] OR "pulp cell*"[tiab] OR "dentin-pulp complex"[tiab] OR "dentin pulp complex"[tiab] OR endodont*[tiab] OR "vital pulp therapy"[tiab] OR "regenerative endodontics"[tiab])) NOT ("neoplasms"[Mesh] OR cancer[tiab] OR tumor[tiab]) | 187 |
| Embase | ('cell senescence'/exp OR 'cellular senescence':ti,ab OR senescence:ti,ab OR 'senescent cell*':ti,ab OR 'pulp senescence':ti,ab) AND ('dental pulp'/exp OR 'dental pulp':ti,ab OR 'pulp cell*':ti,ab OR 'dentin pulp complex':ti,ab OR endodont*:ti,ab OR 'vital pulp therapy':ti,ab OR 'regenerative endodontics':ti,ab) | 203 |
| Scielo | (senescência OR "senescência celular" ) AND ( "polpa dentária" OR "complexo dentina-polpa" ) OR ( "materiais odontológicos" OR resinas OR adesivos) | 502 |
| Web Of Science | TS=(("cellular senescence" OR senescence OR "senescent cell*" OR "pulp senescence") AND ("dental pulp" OR "pulp cell*" OR "dentin-pulp complex" OR endodont* OR "vital pulp therapy" OR "regenerative endodontics")) | 216 |
| Scopus | TITLE-ABS-KEY (("cellular senescence" OR senescence OR "senescent cell*" OR "pulp senescence") AND ("dental pulp" OR "pulp cell*" OR "dentin pulp complex" OR "dentin-pulp complex" OR endodont* OR "vital pulp therapy" OR "regenerative endodontics")) | 206 |
| Cochrane Library | ("cellular senescence" OR senescence OR "cell aging" OR "cellular aging") AND ("dental pulp" OR "dentin pulp complex" OR endodont* OR "vital pulp therapy" OR "regenerative endodontics" OR "dental materials" OR "oxidative stress" OR senotherapeutics) | 76 |

**Supplementary Table 1.** Detailed search strategies used in each database. The initial search was conducted on 11 April 2025 and updated on 15 January 2026; all databases were searched on the same day for each search.
