# Supplementary material for: Cellular senescence in the dental pulp and its implications for endodontics: a scoping review
Source: Clin Oral Investig. 2026 Mar 31;30(4):161. doi: 10.1007/s00784-026-06822-x (PMC13035753; doi:10.1007/s00784-026-06822-x)
Supplement: Supplementary file 2 — Supplementary Material 2 (DOCX 28.9 KB) [file 784_2026_6822_MOESM2_ESM.docx]

| Reference |
| --- |
| 1. Dhok M, Kharat A, Bhonde R, Ghangale N. (2024). Evidence based Osteogenic, anti adipogenic and anti- senescence action of Centella asiatica extract on Dental pulp stem cells. International Journal of Ayurvedic Medicine, 15(3), 684–688. |
| 1. Tong Z, Wu J, Gong Q, Yuan Y, Wang S, Jiang W. (2025). Insulin-like growth factor binding protein 7 identified in aged dental pulp by single-cell RNA sequencing. Journal of Advanced Research, 76, 371–385. https://doi.org/10.1016/j.jare.2024.12.018 |
| 1. Bhandi S, Al Khatani A, Sumayli HA, et al. (2021). Comparative analysis of cytokines and growth factors in the conditioned media of stem cells from the pulp of deciduous, young, and old permanent tooth. Saudi Journal of Biological Sciences, 28(6), 3559–3565. https://doi.org/10.1016/j.sjbs.2021.03.031 |
| 1. Morsczeck C, Hullmann M, Reck A, Reichert TE. (2018). The cell cycle regulator protein P16 and the cellular senescence of dental follicle cells. Molecular and Cellular Biochemistry, 439(1), 45–52. https://doi.org/10.1007/s11010-017-3134-6 |
| 1. Xu K, Feng GJ, Feng XM, Huang D, Zheng K, Tang EY. (2016). Hydrogen peroxide accelerates senescence of human dental pulp stem cells. Chinese Journal of Tissue Engineering Research, 20(10), 1481–1487. https://doi.org/10.3969/j.issn.2095-4344.2016.10.016 |
| 1. Chen L, Wang X, Tian S, et al. (2024). Integrin-linked kinase control dental pulp stem cell senescence via the mTOR signaling pathway. Stem Cells, 42(10), 861–873. https://doi.org/10.1093/stmcls/sxae047 |
| 1. Lee YS, Park YH, Hwang G, et al. (2024). Cpne7 deficiency induces cellular senescence and premature aging of dental pulp. Aging Cell, 23(3), e14061. https://doi.org/10.1111/acel.14061 |
| 1. Iezzi I, Cerqueni G, Licini C, Lucarini G, Mattioli Belmonte M. (2019). Dental pulp stem cells senescence and regenerative potential relationship. Journal of Cellular Physiology, 234(5), 7186–7197. https://doi.org/10.1002/jcp.27472 |
| 1. Dong J, Sakai K, Koma Y, et al. (2021). Dental pulp stem cell-derived small extracellular vesicle in irradiation-induced senescence. Biochemical and Biophysical Research Communications, 575, 28–35. https://doi.org/10.1016/j.bbrc.2021.08.046 |
| 1. Ok CY, Park S, Jang HO, Takata T, Bae MK, Kim YD, Ryu MH, Bae SK. (2020). Visfatin Induces Senescence of Human Dental Pulp Cells. Cells, 9(1). https://doi.org/10.3390/cells9010193 |
| 1. Xu J, Hu M, Liu L, Xu X, Xu L, Song Y. (2024). A transcriptomic analysis of dental pulp stem cell senescence in vitro. Biomedical Engineering Online, 23(1), 102. https://doi.org/10.1186/s12938-024-01298-w |
| 1. He X, Yang Z, Chu XY, et al. (2022). ROR2 Downregulation Activates the MSX2/NSUN2/p21 Regulatory Axis and Promotes Dental Pulp Stem Cell Senescence. Stem Cells, 40(3), 290–302. https://doi.org/10.1093/stmcls/sxab024 |
| 1. Zhang Z, Bao Y, Wei P, Yan X, Qiu Q, Qiu L. (2024). Melatonin attenuates dental pulp stem cells senescence due to vitro expansion via inhibiting MMP3. Oral Diseases, 30(4), 2410–2424. https://doi.org/10.1111/odi.14649 |
| 1. de Farias OJ, da Costa Silva Martins G, Martins DCM, et al. (2024). Senescence on Dental Pulp Cells: Effects on Morphology, Migration, Proliferation, and Immune Response. Journal of Endodontics, 50(3), 362–369. https://doi.org/10.1016/j.joen.2023.12.009 |
| 1. Liu C, Lei Y, Zhang H, Wang X, Chen J. (2025). Pleiotrophin prevents H₂O₂-induced senescence of dental pulp stem cells. Journal of Oral Rehabilitation, 52(3), 391–400. https://doi.org/10.1111/joor.13918 |
| 1. Liang H, Li W, Yang H, et al. (2020). FAM96B inhibits the senescence of dental pulp stem cells. Cell Biology International, 44(5), 1193–1203. https://doi.org/10.1002/cbin.11319 |
| 1. Nozu A, Hamano S, Tomokiyo A, et al. (2018). Senescence and odontoblastic differentiation of dental pulp cells. Journal of Cellular Physiology, 234(1), 849–859. https://doi.org/10.1002/jcp.26905 |
| 1. Wang X, Wang L, Zhou L, et al. (2025). NUP62 alleviates senescence and promotes the stemness of human dental pulp stem cells via NSD2-dependent epigenetic reprogramming. International Journal of Oral Science, 17(1), 34. https://doi.org/10.1038/s41368-025-00362-y |
| 1. Alraies A, Alaidaroos NY, Waddington RJ, Moseley R, Sloan AJ. (2017). Variation in human dental pulp stem cell ageing profiles reflect contrasting proliferative and regenerative capabilities. BMC Cell Biology, 18(1), 12. https://doi.org/10.1186/s12860-017-0128-x |
| 1. Ding Y, Ran Y. (2025). OGA promotes human dental pulp stem cell senescence and inhibits mitophagy by inhibition of O-GlcNAcylation of KLF2. BMC Oral Health, 25(1), 595. https://doi.org/10.1186/s12903-025-05927-1 |
| 1. Li B, Xu M, Huang J, Jia R. (2025). FTO Suppresses Dental Pulp Stem Cell Senescence by Destabilizing NOLC1 mRNA. Biomolecules, 15(11). https://doi.org/10.3390/biom15111627 |
| 1. Gu S, Ran S, Liu B, Liang J. (2016). miR-152 induces human dental pulp stem cell senescence by inhibiting SIRT7 expression. FEBS Letters, 590(8), 1123–1131. https://doi.org/10.1002/1873-3468.12138 |
| 1. Zhang Q, Cao S, Zhang M, Wu D, Zhang C, Li J. (2025). Biodentine Counteracts the Aging Process of Human Dental Pulp Stem Cells Through Wnt/β-Catenin Pathway. International Dental Journal, 75(4), 100819. https://doi.org/10.1016/j.identj.2025.03.028 |
| 1. Liu H, Gong Y, Nakagawa M, et al. (2023). Localization of senescent cells under cavity preparations in rats and restoration of reparative dentin formation by senolytics. Dental Materials Journal, 42(3), 360–367. https://doi.org/10.4012/dmj.2022-245 |
| 1. Yang RL, Huang HM, Han CS, Cui SJ, Zhou YK, Zhou YH. (2021). Serine Metabolism Controls Dental Pulp Stem Cell Aging by Regulating the DNA Methylation of p16. Journal of Dental Research, 100(1), 90–97. https://doi.org/10.1177/0022034520958374 |
| 1. da Silva CJ, Ramos RF, da Silva AASV, et al. (2025). Impact of Cellular Senescence on the Immune-Inflammatory Response and Regenerative Capacity of Human Dental Pulp Cells. Journal of Endodontics, 51(11), 1590–1598. https://doi.org/10.1016/j.joen.2025.07.009 |
| 1. Song Y, Zhang S, Cao C, et al. (2024). Imaging Structural and Electrical Changes of Aging Cells Using Scanning Ion Conductance Microscopy. Small Methods, 8(8), e2301315. https://doi.org/10.1002/smtd.202301315 |
| 1. Vongprommool A, Mutirangura A, Pavasant P, Subbalekha K. (2024). Alu methylation level, morphological, and senescence changes during in vitro aging of human dental pulp stem cells. Tissue & Cell, 90, 102512. https://doi.org/10.1016/j.tice.2024.102512 |
| 1. Feng X, Feng G, Xing J, et al. (2014). Repeated lipopolysaccharide stimulation promotes cellular senescence in human dental pulp stem cells (DPSCs). Cell and Tissue Research, 356(2), 369–380. https://doi.org/10.1007/s00441-014-1799-7 |
| 1. Zayed M, Iohara K. (2020). Effects of p-Cresol on Senescence, Survival, Inflammation, and Odontoblast Differentiation in Canine Dental Pulp Stem Cells. International Journal of Molecular Sciences, 21(18). https://doi.org/10.3390/ijms21186931 |
| 1. Lyu X, Xu W, Zhou J, et al. (2025). Molecular changes of cellular senescence in dental pulp stem cells during in vitro culture: A potential role of PSG4. Tissue & Cell, 93, 102758. https://doi.org/10.1016/j.tice.2025.102758 |
| 1. Yi Q, Liu O, Yan F, et al. (2017). Analysis of Senescence-Related Differentiation Potentials and Gene Expression Profiles in Human Dental Pulp Stem Cells. Cells, Tissues, Organs, 203(1), 1–11. https://doi.org/10.1159/000448026 |
| 1. Shi Y, Xiao T, Weng Y, et al. (2024). 3D culture inhibits replicative senescence of SCAPs via UQCRC2-mediated mitochondrial oxidative phosphorylation. Journal of Translational Medicine, 22(1), 1129. https://doi.org/10.1186/s12967-024-05953-7 |
| 1. Li L, Zhu YQ, Jiang L, Peng W. (2012). Increased autophagic activity in senescent human dental pulp cells. International Endodontic Journal, 45(12), 1074–1079. https://doi.org/10.1111/j.1365-2591.2012.02064.x |
| 1. Macrin D, Alghadeer A, Zhao YT, et al. (2019). Metabolism as an early predictor of DPSCs aging. Scientific Reports, 9(1), 2195. https://doi.org/10.1038/s41598-018-37489-4 |
| 1. Das M, Das A, Barui A, Paul RR. (2022). Comparative evaluation of proliferative potential and replicative senescence associated changes in mesenchymal stem cells derived from dental pulp and umbilical cord. Cell and Tissue Banking, 23(1), 157–170. https://doi.org/10.1007/s10561-021-09926-8 |
| 1. Li Z, Chu X, Guo J, et al. (2025). Resveratrol delays senescence of human dental pulp stem cells via activating the SIRT1-mitochondrial autophagy. Scientific Reports, 15(1), 41354. https://doi.org/10.1038/s41598-025-25240-9 |
| 1. Zhang R, Chen J, Chen Y, Li Y. (2025). SIRT7 promotes dental pulp stem cells replicative senescence through desuccinylation of ROCK1. Tissue & Cell, 92, 102636. https://doi.org/10.1016/j.tice.2024.102636 |
| 1. Yaghoobi MM, Sheikoleslami M, Ebrahimi M. (2020). Effects of hydrogen peroxide, doxorubicin and ultraviolet irradiation on senescence of human dental pulp stem cells. Archives of Oral Biology, 117, 104819. https://doi.org/10.1016/j.archoralbio.2020.104819 |
| 1. Zayed M, Iohara K, Watanabe H, Nakashima M. (2020). CCR3 antagonist protects against induced cellular senescence and promotes rejuvenation in periodontal ligament cells for stimulating pulp regeneration in the aged dog. Scientific Reports, 10(1), 8631. https://doi.org/10.1038/s41598-020-65301-9 |
| 1. Choi YJ, Lee JY, Chung CP, Park YJ. (2012). Cell-penetrating superoxide dismutase attenuates oxidative stress-induced senescence by regulating the p53-p21(Cip1) pathway and restores osteoblastic differentiation in human dental pulp stem cells. International Journal of Nanomedicine, 7, 5091–5106. https://doi.org/10.2147/IJN.S31723 |
| 1. Mas-Bargues C, Viña-Almunia J, Inglés M, et al. (2017). Role of p16(INK4a) and BMI-1 in oxidative stress-induced premature senescence in human dental pulp stem cells. Redox Biology, 12, 690–698. https://doi.org/10.1016/j.redox.2017.04.002 |
| 1. Ou Y, Zhou Y, Liang S, Wang Y. (2018). Sclerostin promotes human dental pulp cells senescence. PeerJ, 6, e5808. https://doi.org/10.7717/peerj.5808 |
| 1. Zhang L, Xia D, Wang C, Gao F, Hu L, Li J, Jin L. (2023). Pleiotrophin attenuates the senescence of dental pulp stem cells. Oral Diseases, 29(1), 195–205. https://doi.org/10.1111/odi.13929 |
| 1. Muthna D, Soukup T, Vavrova J, et al. (2010). Irradiation of adult human dental pulp stem cells provokes activation of p53, cell cycle arrest, and senescence but not apoptosis. Stem Cells and Development, 19(12), 1855–1862. https://doi.org/10.1089/scd.2009.0449 |
| 1. Wang K, Li L, Wu J, Qiu Q, Zhou F, Wu H. (2015). The different expression profiles of microRNAs in elderly and young human dental pulp and the role of miR-433 in human dental pulp cells. Mechanisms of Ageing and Development, 146-148, 1–11. https://doi.org/10.1016/j.mad.2015.03.001 |
| 1. Zou XY, Zhuang H, Yue L, Gao XJ. (2010). Involvement of Notch signalling pathway in senescence of human dental pulp cells. The Chinese Journal of Dental Research, 13(1), 45–49. https://pubmed.ncbi.nlm.nih.gov/20936191/ |
| 1. Lee YH, Kim GE, Cho HJ, et al. (2013). Aging of in vitro pulp illustrates change of inflammation and dentinogenesis. Journal of Endodontics, 39(3), 340–345. https://doi.org/10.1016/j.joen.2012.10.031 |
| 1. Asghari M, Nasoohi N, Hodjat M. (2021). High glucose promotes the aging of human dental pulp cells through Wnt/beta-catenin signaling. Dental and Medical Problems, 58(1), 39–46. https://doi.org/10.17219/dmp/130090 |
| 1. Dou W, Xie J, Chen J, et al. (2023). Overexpression of adrenomedullin (ADM) alleviates the senescence of human dental pulp stem cells by regulating the miR-152/CCNA2 pathway. Cell Cycle, 22(5), 565–579. https://doi.org/10.1080/15384101.2022.2135621 |
| 1. Liu L, Wei X, Ling J, Wu L, Xiao Y. (2011). Expression pattern of Oct-4, Sox2, and c-Myc in the primary culture of human dental pulp derived cells. Journal of Endodontics, 37(4), 466–472. https://doi.org/10.1016/j.joen.2010.12.012 |
| 1. Ghaffari M, Shrestha A. (2025). Optimizing Stem Cell Expansion: The Role of Substrate Stiffness in Enhancing Dental Pulp Stem Cell Quiescence and Regeneration. Journal of Endodontics, 51(4), 491–498. https://doi.org/10.1016/j.joen.2025.01.004 |
| 1. Yao L, Li F, Yu C, Wang H, Wang Y, Ye L, Yu F. (2023). Chronological and Replicative Aging of CD51(+)/PDGFR-α(+) Pulp Stromal Cells. Journal of Dental Research, 102(8), 929–937. https://doi.org/10.1177/00220345231158038 |
| 1. Tang W, Wang W, Wan X, et al. (2025). The Role of Abnormal Mitochondrial Fusion and Fission in the Inflammatory Senescence of Dental Pulp Stem Cells. International Dental Journal, 75(6), 103877. https://doi.org/10.1016/j.identj.2025.103877 |
| 1. Horibe H, Murakami M, Iohara K, et al. (2014). Isolation of a stable subpopulation of mobilized dental pulp stem cells (MDPSCs) with high proliferation, migration, and regeneration potential is independent of age. PLoS One, 9(5), e98553. https://doi.org/10.1371/journal.pone.0098553 |
| 1. Nakashima M, Iohara K. (2014). Mobilized dental pulp stem cells for pulp regeneration: initiation of clinical trial. Journal of Endodontics, 40(4), S26-32. https://doi.org/10.1016/j.joen.2014.01.020 |
| 1. Feng X, Xing J, Feng G, et al. (2014). p16(INK4A) mediates age-related changes in mesenchymal stem cells derived from human dental pulp through the DNA damage and stress response. Mechanisms of Ageing and Development, 141, 46–55. https://doi.org/10.1016/j.mad.2014.09.004 |
| 1. Zhai Y, Wei R, Liu J, et al. (2017). Drug-induced premature senescence model in human dental follicle stem cells. Oncotarget, 8(5), 7276–7293. https://doi.org/10.18632/oncotarget.14085 |
| 1. Ok CY, Park S, Jang HO, Takata T, Lee OH, Bae MK, Bae SK. (2021). FK866 Protects Human Dental Pulp Cells against Oxidative Stress-Induced Cellular Senescence. Antioxidants, 10(2). https://doi.org/10.3390/antiox10020271 |
| 1. Hu M, Zhang Q, Xu J, et al. (2025). New considerations in selecting donors for dental pulp stem cells: a pilot study. Biomedical Engineering Online, 24(1), 37. https://doi.org/10.1186/s12938-025-01367-8 |
| 1. Zhou S, Cui J, Shi Y. (2024). Serine Metabolism Regulates the Replicative Senescence of Human Dental Pulp Cells through Histone Methylation. Current Issues in Molecular Biology, 46(4), 2856–2870. https://doi.org/10.3390/cimb46040179 |
| 1. Zhang L, Pang B, Wang R, Yang B, Jia X. (2024). Nesfatin-1 attenuated lipopolysaccharide-induced inflammatory response and senescence in human dental pulp cells. Heliyon, 10(12), e32108. https://doi.org/10.1016/j.heliyon.2024.e32108 |
| 1. Ok CY, Park S, Jang HO, Bae MK, Bae SK. (2023). Involvement of the visfatin/toll-like receptor 4 signaling axis in human dental pulp cell senescence: Protection via toll-like receptor 4 blockade. Journal of Dental Sciences, 18(3), 1177–1188. https://doi.org/10.1016/j.jds.2022.10.008 |
| 1. Sattari M, Masoudnia M, Mashayekhi K, et al. (2022). Evaluating the effect of LPS from periodontal pathogenic bacteria on the expression of senescence-related genes in human dental pulp stem cells. Journal of Cellular and Molecular Medicine, 26(22), 5647–5656. https://doi.org/10.1111/jcmm.17594 |
| 1. Alaidaroos NYA, Alraies A, Waddington RJ, Sloan AJ, Moseley R. (2021). Differential SOD2 and GSTZ1 profiles contribute to contrasting dental pulp stem cell susceptibilities to oxidative damage and premature senescence. Stem Cell Research & Therapy, 12(1), 142. https://doi.org/10.1186/s13287-021-02209-9 |
| 1. Ma D, Ma Z, Zhang X, et al. (2009). Effect of age and extrinsic microenvironment on the proliferation and osteogenic differentiation of rat dental pulp stem cells in vitro. Journal of Endodontics, 35(11), 1546–1553. https://doi.org/10.1016/j.joen.2009.07.016 |
| 1. Meng H, Wei F, Ge Z, et al. (2022). Long-term hypoxia inhibits the passage-dependent stemness decrease and senescence increase of human dental pulp stem cells. Tissue & Cell, 76, 101819. https://doi.org/10.1016/j.tice.2022.101819 |
| 1. Luo H, Liu W, Zhang Y, Yang Y, Jiang X, Wu S, Shao L. (2021). METTL3-mediated m(6)A modification regulates cell cycle progression of dental pulp stem cells. Stem Cell Research & Therapy, 12(1), 159. https://doi.org/10.1186/s13287-021-02223-x |
| 1. Zhang L, Luo D, Bai L. (2026). Apelin-12 Attenuates LPS-Induced Cellular Senescence in Human Dental Pulp Cells via SIRT6-Mediated Pathways: Implications for Gingivitis Management. Molecular Oral Microbiology, 41(1), 48–56. https://doi.org/10.1111/omi.70012 |
| 1. Cui Y, Ji W, Gao Y, Xiao Y, Liu H, Chen Z. (2021). Single-cell characterization of monolayer cultured human dental pulp stem cells with enhanced differentiation capacity. International Journal of Oral Science, 13(1), 44. https://doi.org/10.1038/s41368-021-00140-6 |
| 1. Xing Y, Zhang Y, Wu X, Zhao B, Ji Y, Xu X. (2019). A comprehensive study on donor-matched comparisons of three types of mesenchymal stem cells-containing cells from human dental tissue. Journal of Periodontal Research, 54(3), 286–299. https://doi.org/10.1111/jre.12630 |
| 1. Lu H, Shi F, Wang B, Zheng Y, Lu J, Zeng B, Zhao W. (2025). Molecular Biological Comparison of Pulp Stem Cells from Supernumerary Teeth, Permanent Teeth, and Deciduous Teeth for Endodontic Regeneration. International Journal of Molecular Sciences, 26(5), 1933. https://doi.org/10.3390/ijms26051933 |
| 1. Wang X, Cai Y, Zhang M, Xu J, Zhang C, Li J. (2022). Effect of Biodentine on Odonto/Osteogenic Differentiation of Human Dental Pulp Stem Cells. Bioengineering, 10(1). https://doi.org/10.3390/bioengineering10010012 |
| 1. Dieterle MP, Gross T, Steinberg T, et al. (2022). Characterization of a Stemness-Optimized Purification Method for Human Dental-Pulp Stem Cells: An Approach to Standardization. Cells, 11(20). https://doi.org/10.3390/cells11203204 |
| 1. Chang SJ, Zou XY, Zhuang H, Yue L, Gao XJ. (2014). [Notch activation delayed ageing of human dental pulp cells]. Journal of Peking University. Health Sciences, 46(1), 5–11. https://pubmed.ncbi.nlm.nih.gov/24535339/ |
| 1. Dong XY, Huang YX, Yang Z, et al. (2021). Downregulation of ROR2 promotes dental pulp stem cell senescence by inhibiting STK4-FOXO1/SMS1 axis in sphingomyelin biosynthesis. Aging Cell, 20(8), e13430. https://doi.org/10.1111/acel.13430 |
| 1. Wang H, Zhong Q, Yang T, et al. (2018). Comparative characterization of SHED and DPSCs during extended cultivation in vitro. Molecular Medicine Reports, 17(5), 6551–6559. https://doi.org/10.3892/mmr.2018.8725 |
| 1. Liao C, Wang Y, Ou Y, Wu Y, Zhou Y, Liang S. (2019). Effects of sclerostin on lipopolysaccharide-induced inflammatory phenotype in human odontoblasts and dental pulp cells. The International Journal of Biochemistry & Cell Biology, 117, 105628. https://doi.org/10.1016/j.biocel.2019.105628 |
| 1. Vaseenon S, Apaijai N, Pratchayasakul W, Chattipakorn N, Chattipakorn SC. (2025). D-galactose-induced aging and obese conditions contribute to aging and pathologies in dental pulp of male wistar rats. Experimental Gerontology, 211, 112907. https://doi.org/10.1016/j.exger.2025.112907 |
| 1. Mas-Bargues C, Sanz-Ros J, Romero-García N, Huete-Acevedo J, Dromant M, Borrás C. (2023). Small extracellular vesicles from senescent stem cells trigger adaptive mechanisms in young stem cells by increasing antioxidant enzyme expression. Redox Biology, 62, 102668. https://doi.org/10.1016/j.redox.2023.102668 |
| 1. Peng X, Zhao L, Wang J, Zhang Y, Liu Z, Wang K, Zhang L. (2025). Melatonin Alleviates Oxidative Stress-Induced Mitochondrial Dysfunction Through Ameliorating NAD(+) Homeostasis of hDPSCs for Cell-Based Therapy. Journal of Pineal Research, 77(3), e70058. https://doi.org/10.1111/jpi.70058 |
| 1. Zhang S, Zhang R, Qiao P, et al. (2021). Metformin-Induced MicroRNA-34a-3p Downregulation Alleviates Senescence in Human Dental Pulp Stem Cells by Targeting CAB39 through the AMPK/mTOR Signaling Pathway. Stem Cells International, 2021, 6616240. https://doi.org/10.1155/2021/6616240 |
| 1. Malik MA, Perkins E, Nedell A, et al. (2025). Viability of Dental Pulp Derived Stem Cells After Long-Term Cryopreservation. Journal of Endodontics, 51(12), 1775-1782.e2. https://doi.org/10.1016/j.joen.2025.09.014 |
| 1. Diomede F, Tripodi D, Trubiani O, Pizzicannella J. (2019). HEMA Effects on Autophagy Mechanism in Human Dental Pulp Stem Cells. Materials, 12(14). https://doi.org/10.3390/ma12142285 |
| 1. Ma L, Hu J, Cao Y, et al. (2019). Maintained Properties of Aged Dental Pulp Stem Cells for Superior Periodontal Tissue Regeneration. Aging and Disease, 10(4), 793–806. https://doi.org/10.14336/AD.2018.0729 |
| 1. Sun Q, Bai R, Chen S, et al. (2024). Lysine demethylase 3A promotes chondrogenic differentiation of aged human dental pulp stem cells. Journal of Dental Sciences, 19(1), 86–91. https://doi.org/10.1016/j.jds.2023.05.030 |
| 1. Feng G, Zheng K, Cao T, et al. (2018). Repeated stimulation by LPS promotes the senescence of DPSCs via TLR4/MyD88-NF-κB-p53/p21 signaling. Cytotechnology, 70(3), 1023–1035. https://doi.org/10.1007/s10616-017-0180-6 |
| 1. Iezzi I, Lazzarini R, Cerqueni G, et al. (2021). MicroRNA Profiling in Mesenchymal Stromal Cells: the Tissue Source as the Missing Piece in the Puzzle of Ageing. Stem Cell Reviews and Reports, 17(3), 1014–1026. https://doi.org/10.1007/s12015-020-10095-6 |
| 1. Kharat A, Sakhare S, Sanap A, Potdar P, Kheur S, Bhonde R. (2025). Beneficial effect of cow urine on stem cell differentiation, senescence, oxidative stress and angiogenesis in chick yolk sac model. Journal of Ayurveda and Integrative Medicine, 16(4), 101194. https://doi.org/10.1016/j.jaim.2025.101194 |
| 1. Simon S, Smith AJ, Lumley PJ, et al. (2009). Molecular characterization of young and mature odontoblasts. Bone, 45(4), 693–703. https://doi.org/10.1016/j.bone.2009.06.018 |
| 1. Ghaffari M, Shrestha A. (2026). Mechanical Memory and NF-κB Signaling in Dental Pulp Stem Cell Odontogenic Differentiation. Journal of Endodontics. https://doi.org/10.1016/j.joen.2026.01.001 |
| 1. Ning T, Shao J, Zhang X, et al. (2020). Ageing affects the proliferation and mineralization of rat dental pulp stem cells under inflammatory conditions. International Endodontic Journal, 53(1), 72–83. https://doi.org/10.1111/iej.13205 |
| 1. Couve E, Schmachtenberg O. (2011). Autophagic activity and aging in human odontoblasts. Journal of Dental Research, 90(4), 523–528. https://doi.org/10.1177/0022034510393347 |
| 1. Saiyasilp S, Vaseenon S, Srisuwan T, Chuveera P. (2025). Effects of D-galactose Induction on Aging Characteristics of the Human Dental Pulp Cell Culture Model: An In Vitro Study. European Endodontic Journal, 10(2), 142–150. https://doi.org/10.14744/eej.2024.15010 |

**Supplementary table 3.** Included articles (n=94).
