# Supplementary material for: Cellular senescence in the dental pulp and its implications for endodontics: a scoping review
Source: Clin Oral Investig. 2026 Mar 31;30(4):161. doi: 10.1007/s00784-026-06822-x (PMC13035753; doi:10.1007/s00784-026-06822-x)
Supplement: Supplementary file 3 — Supplementary Material 3 (DOCX 31.2 KB) [file 784_2026_6822_MOESM3_ESM.docx]

| Title of the article | Citation count (Web of Science) | Citation count (Elsevier Scopus) | Citation  count  (Dimensions) | | Citation  count  (Google  Scholar) | Citation  density |
| --- | --- | --- | --- | --- | --- | --- |
| 1. Murray, P. E., Stanley, H. R., Matthews, J. B., Sloan, A. J., & Smith, A. J. (2002). Age-related odontometric changes of human teeth. Oral Surgery, Oral Medicine, Oral Pathology, Oral Radiology, and Endodontology, 93(4), 474–482. | 159 | 189 | 192 | 333 | | 9,9 |
| 2. Sundar, I. K., Javed, F., Romanos, G. E., & Rahman, I. (2016). E-cigarettes and flavorings induce inflammatory and pro-senescence responses in oral epithelial cells and periodontal fibroblasts. Oncotarget, 7(47), 77196–77204. | 148 | 158 | 184 | 261 | | 23,4 |
| 3. Fujihashi, K., & Kiyono, H. (2009). Mucosal immunosenescence: new developments and vaccines to control infectious diseases. Trends in Immunology, 30(7), 334–343. | 84 | 94 | 102 | 135 | | 6,9 |
| 4. Kang, M., Kameta, A., Shin, K., Baluda, M., Kim, H., & Park, N. (2003). Senescence-associated genes in normal human oral keratinocytes. *Experimental Cell Research*, *287*(2), 272–281. | 80 | 87 | 79 | 107 | | 4,2 |
| 5. Lu, S. Y., Chang, K. W., Liu, C. J., Tseng, Y. H., Lu, H. H., Lee, S. Y., & Lin, S.-C. (2006). Ripe areca nut extract induces G1 phase arrests and senescence-associated phenotypes in normal human oral keratinocyte. *Carcinogenesis*, *27*(6), 1273–1284. | 72 | 81 | 81 | 113 | | 4,8 |
| 6. Alraies, A., Alaidaroos, N. Y. A., Waddington, R. J., Moseley, R., & Sloan, A. J. (2017). Variation in human dental pulp stem cell ageing profiles reflect contrasting proliferative and regenerative capabilities. *BMC Cell Biology*, *18*(12), 1-14. | 73 | 81 | 82 | 106 | | 12,2 |
| 7. Kim, R. H., Lee, R. S., Williams, D., Bae, S., Woo, J., Lieberman, M., Oh, J.-E., Dong, Q., Shin, K.-H., Kang, M. K., & Park, N. H. (2011). Bisphosphonates Induce Senescence in Normal Human Oral Keratinocytes. *Journal of Dental Research*, *90*(6), 623-630. | 59 | 61 | 67 | 105 | | 5,6 |
| 8. Shin, K.-H., Kang, M. K., Dicterow, E., & Park, N. H. (2003). Hypermethylation of the hTERT promoter inhibits the expression of telomerase activity in normal oral fibroblasts and senescent normal oral keratinocytes. *British Journal of Cancer*, *89*(8), 1473–1478. | 53 | 56 | 56 | 95 | | 3,0 |
| 9. Cáceres, M., Oyarzun, A., & Smith, P. C. (2014). Defective Wound-healing in Aging Gingival Tissue. *Journal of Dental Research*, *93*(7), 691–697. | 54 | 60 | 64 | 80 | | 6,4 |
| 10. Ikawa, M., Komatsu, H., Ikawa, K., Mayanagi, H., & Shimauchi, H. (2003). Age‐related changes in the human pulpal blood flow measured by laser Doppler flowmetry. *Dental Traumatology*, *19*(1), 36–40. | 42 | 42 | 58 | 102 | | 2,9 |
| 11. Preshaw, P. M., Henne, K., Taylor, J. J., Valentine, R. A., & Conrads, G. (2017). Age‐related changes in immune function (immune senescence) in caries and periodontal diseases: a systematic review. *Journal of Clinical Periodontology*, *44*(18), 153–177. | 45 | 47 | 57 | 78 | | 8,1 |
| 12. Mehrazarin, S., Oh, J. E., Chung, C. L., Chen, W., Kim, R. H., Shi, S., Park, N.-H., & Kang, M. K. (2011). Impaired Odontogenic Differentiation of Senescent Dental Mesenchymal Stem Cells Is Associated with Loss of Bmi-1 Expression. *Journal of Endodontics*, *37*(5), 662–666. | 45 | 56 | 51 | 70 | | 4,2 |
| 13. Weng, J.-H., Yu, C.-C., Lee, Y.-C., Lin, C.-W., Chang, W.-W., & Kuo, Y.-L. (2016). miR-494-3p Induces Cellular Senescence and Enhances Radiosensitivity in Human Oral Squamous Carcinoma Cells. *International Journal of Molecular Sciences*, *17*(7), 1092-2002. | 49 | 51 | 47 | 57 | | 6,3 |
| 14. Maeda, H. (2020). Aging and Senescence of Dental Pulp and Hard Tissues of the Tooth. *Frontiers in Cell and Developmental Biology*, *8*(1), 1-9. | 43 | 44 | 49 | 62 | | 12,3 |
| 15. Robles, S. J., Buehler, P. W., Negrusz, A., & Adami, G. R. (1999). Permanent cell cycle arrest in asynchronously proliferating normal human fibroblasts treated with doxorubicin or etoposide but not camptothecin. *Biochemical Pharmacology*, *58*(4), 675–685. | 40 | 45 | 46 | 63 | | 1,9 |
| 16. Lee, Y. H., Kim, G. E., Cho, H. J., Yu, M. K., Bhattarai, G., Lee, N. H., & Yi, H. K. (2013). Aging of *In vitro* Pulp Illustrates Change of Inflammation and Dentinogenesis. *Journal of Endodontics*, *39*(3), 340–345. | 39 | 43 | 43 | 61 | | 4,2 |
| 17. Iezzi, I., Pagella, P., Mattioli-Belmonte, M., & Mitsiadis, T. (2019). The effects of ageing on dental pulp stem cells, the tooth longevity elixir. *European Cells and Materials*, *37*(1), 175–185. | 36 | 38 | 42 | 58 | | 8,7 |
| 18. Mas-Bargues, C., Viña-Almunia, J., Inglés, M., Sanz-Ros, J., Gambini, J., Ibáñez-Cabellos, J. S., García-Giménez, J. L., Viña, J., & Borrás, C. (2017). Role of p16INK4a and BMI-1 in oxidative stress-induced premature senescence in human dental pulp stem cells. *Redox Biology*, *12*(1), 690–698. | 39 | 41 | 43 | 50 | | 6,1 |
| 19. Satoh, R., Kishino, K., Morshed, S. R. M., Takayama, F., Otsuki, S., Suzuki, F., Hashimoto, K., Kikuchi, H., Nishikawa, H., Yasui, T., & Sakagami, H. (2005). Changes in fluoride sensitivity during *in vitro* senescence of normal human oral cells. *Anticancer Research*, *25*(3), 2085–2090. | 42 | 46 | 11 | 59 | | 2,0 |
| 20. Schoetz, U., Klein, D., Hess, J., Shnayien, S., Spoerl, S., Orth, M., Mutlu, S., Hennel, R., Sieber, A., Ganswindt, U., Luka, B., Thomsen, A. R., Unger, K., Jendrossek, V., Zitzelsberger, H., Blüthgen, N., Belka, C., Unkel, S., Klinger, B., & Lauber, K. (2021). Early senescence and production of senescence-associated cytokines are major determinants of radioresistance in head-and-neck squamous cell carcinoma. *Cell Death & Disease*, *12*(12), 1162-1176. | 34 | 36 | 41 | 47 | | 13,1 |
| 21. Kang, M. K., Kameta, A., Shin, K., Baluda, M. A., & Park, N. (2004). Senescence occurs with *hTERT* repression and limited telomere shortening in human oral keratinocytes cultured with feeder cells. *Journal of Cellular Physiology*, *199*(3), 364–370. | 34 | 35 | 37 | 47 | | 1,9 |
| 22. Kang, M. K., & Park, M.H. (2001). Conversion of Normal To Malignant Phenotype: Telomere Shortening, Telomerase Activation, and Genomic Instability During Immortalization of Human Oral Keratinocytes. *Critical Reviews in Oral Biology & Medicine*, *12*(1), 38–54. | 31 | 36 | 31 | 52 | | 1,6 |
| 23. Kim, E. K., Moon, S., Kim, D. K., Zhang, X., & Kim, J. (2018). CXCL1 induces senescence of cancer-associated fibroblasts via autocrine loops in oral squamous cell carcinoma. *Plos One*, *13*(1), 1-17. | 29 | 34 | 39 | 48 | | 6,2 |
| 24. Benatti, B. B., Silvério, K. G., Casati, M. Z., Sallum, E. A., & Nociti, F. H. (2008). Influence of Aging on Biological Properties of Periodontal Ligament Cells. *Connective Tissue Research*, *49*(6), 401–408. | 29 | 27 | 33 | 53 | | 2,2 |
| 25. Choi, Y. J., Lee, J. Y., Chung, C. P., & Park, Y. J. (2012). Cell-penetrating superoxide dismutase attenuates oxidative stress-induced senescence by regulating the p53-p21Cip1 pathway and restores osteoblastic differentiation in human dental pulp stem cells. *International Journal of Nanomedicine*, *2012*(7), 5091-5106. | 29 | 31 | 34 | 46 | | 2,9 |
| 26. Murray, P. E., Matthews, J. B., Sloan, A. J., & Smith, A. J. (2002). Analysis of incisor pulp cell populations in Wistar rats of different ages. *Archives of Oral Biology*, *47*(10), 709–715. | 24 | 30 | 32 | 51 | | 1,5 |
| 27. Prime, S. S., Cirillo, N., Hassona, Y., Lambert, D. W., Paterson, I. C., Mellone, M., Thomas, G. J., James, E. N. L., & Parkinson, E. K. (2017). Fibroblast activation and senescence in oral cancer. *Journal of Oral Pathology & Medicine*, *46*(2), 82–88. | 24 | 30 | 34 | 47 | | 4,8 |
| 28. Shimizu, N., Yamaguchi, M., Uesu, K., Goseki, T., & Abiko, Y. (2000). Stimulation of Prostaglandin E2 and Interleukin-1 Production From Old Rat Periodontal Ligament Cells Subjected to Mechanical Stress. *The Journals of Gerontology Series A: Biological Sciences and Medical Sciences*, *55*(10), 489–495. | 25 | 32 | 27 | 46 | | 1,3 |
| 29. Macrin, D., Alghadeer, A., Zhao, Y. T., Miklas, J. W., Hussein, A. M., Detraux, D., Robitaille, A. M., Madan, A., Moon, R. T., Wang, Y., Devi, A., Mathieu, J., & Ruohola-Baker, H. (2019). Metabolism as an early predictor of DPSCs aging. *Scientific Reports*, *9*(1), 2195-2214. | 25 | 26 | 30 | 43 | | 6,2 |
| 30. Campo-Trapero, J., Cano-Sánchez, J., Palacios-Sánchez, B., Llamas-Martínez, S., lo muzio, L., & Bascones-Martínez, A. (2008). Cellular senescence in oral cancer and precancer and treatment implications: A review. *Acta Oncologica*, *47*(8), 1464–1474. | 28 | 27 | 30 | 37 | | 1,9 |
| 31. Bae, W.-J., Park, J. S., Kang, S.-K., Kwon, I.-K., & Kim, E.-C. (2018). Effects of Melatonin and Its Underlying Mechanism on Ethanol-Stimulated Senescence and Osteoclastic Differentiation in Human Periodontal Ligament Cells and Cementoblasts. *International Journal of Molecular Sciences*, *19*(6), 1742-1763. | 26 | 29 | 32 | 33 | | 5,0 |
| 32. Shiba, H., Nakanishi, K., Sakata, M., Fujita, T., Uchida, Y., & Kurihara, H. (2000). Effects of ageing on proliferative ability, and the expressions of secreted protein, acidic and rich in cysteine (SPARC) and osteoprotegerin (osteoclastogenesis inhibitory factor) in cultures of human periodontal ligament cells. *Mechanisms of Ageing and Development*, *117*(1–3), 69–77. | 25 | 25 | 27 | 42 | | 1,2 |
| 33. Li, L., Zhu, Y. Q., Jiang, L., & Peng, W. (2012). Increased autophagic activity in senescent human dental pulp cells. *International Endodontic Journal*, *45*(12), 1074–1079. | 24 | 27 | 27 | 39 | | 2,4 |
| 34. Streckfus, C., Bigler, L., & O’Bryan, T. (2002). Aging and Salivary Cytokine Concentrations as Predictors of Whole Saliva Flow Rates among Women. *Gerontology*, *48*(5), 282–288. | 26 | 26 | 25 | 39 | | 1,3 |
| 35. Ahn, S.-H., Chun, S.-M., Park, C., Lee, J.-H., Lee, S.-W., & Lee, T.-H. (2017). Transcriptome profiling analysis of senescent gingival fibroblasts in response to Fusobacterium nucleatum infection. *PLOS ONE*, *12*(11), 1-19. | 23 | 26 | 27 | 39 | | 4,1 |
| 36. Çelenligil‐Nazliel, H., Ayhan, A., Uzun, H., & Ruacan, Ş. (2000). The Effect of Age on Proliferating Cell Nuclear Antigen Expression in Oral Gingival Epithelium of Healthy and Inflamed Human Gingiva. *Journal of Periodontology*, *71*(10), 1567–1574. | 22 | 23 | 28 | 39 | | 1,1 |
| 37. Ohzeki, K., Yamaguchi, M., Shimizu, N., & Abiko, Y. (1999). Effect of cellular aging on the induction of cyclooxygenase-2 by mechanical stress in human periodontal ligament cells. *Mechanisms of Ageing and Development*, *108*(2), 151–163. | 22 | 26 | 24 | 36 | | 1,0 |
| 38. Jang, D. H., Bhawal, U. K., Min, H.-K., Kang, H. K., Abiko, Y., & Min, B.-M. (2015). A Transcriptional Roadmap to the Senescence and Differentiation of Human Oral Keratinocytes. *The Journals of Gerontology: Series A*, *70*(1), 20–32. | 21 | 23 | 27 | 30 | | 2,8 |
| 39. Yue, Z., Nie, L., Zhao, P., Ji, N., Liao, G., & Wang, Q. (2022). Senescence-associated secretory phenotype and its impact on oral immune homeostasis. *Frontiers in Immunology*, *13*(1), 1-14. | 21 | 21 | 24 | 26 | | 11,5 |
| 40. Miura, S., Yamaguchi, M., Shimizu, N., & Abiko, Y. (2000). Mechanical stress enhances expression and production of plasminogen activator in aging human periodontal ligament cells. *Mechanisms of Ageing and Development*, *112*(3), 217–231. | 19 | 19 | 21 | 27 | | 0,8 |
| 41. Parkinson, E. K. (2010). Senescence as a modulator of oral squamous cell carcinoma development. *Oral Oncology*, *46*(12), 840–853. | 16 | 20 | 20 | 29 | | 1,5 |
| 42. Tan, M. L., Parkinson, E. K., Yap, L. F., & Paterson, I. C. (2021). Autophagy is deregulated in cancer-associated fibroblasts from oral cancer and is stimulated during the induction of fibroblast senescence by TGF-β1. *Scientific Reports*, *11*(1), 584-598. | 18 | 20 | 22 | 25 | | 7,0 |
| 43. Sanders, A. E., Divaris, K., Naorungroj, S., Heiss, G., & Risques, R. A. (2015). Telomere length attrition and chronic periodontitis: an ARIC Study nested case-control study. *Journal of Clinical Periodontology*, *42*(1), 12–20. | 17 | 19 | 21 | 27 | | 2,3 |
| 44. Steffens, J. P., Masi, S., D’Aiuto, F., & Spolidorio, L. C. (2013). Telomere length and its relationship with chronic diseases – New perspectives for periodontal research. *Archives of Oral Biology*, *58*(2), 111–117. | 15 | 18 | 17 | 33 | | 1,8 |
| 45. Saldías, M. P., Fernández, C., Morgan, A., Díaz, C., Morales, D., Jaña, F., Gómez, A., Silva, A., Briceño, F., Oyarzún, A., Maldonado, F., Cerda, O., Smith, P. C., & Cáceres, M. (2017). Aged blood factors decrease cellular responses associated with delayed gingival wound repair. *Plos One*, *12*(9), 1-16. | 20 | 19 | 16 | 25 | | 2,8 |
| 46. Konstantonis, D., Papadopoulou, A., Makou, M., Eliades, T., Basdra, E., & Kletsas, D. (2014). The role of cellular senescence on the cyclic stretching-mediated activation of MAPK and ALP expression and activity in human periodontal ligament fibroblasts. *Experimental Gerontology*, *57*, 175–180. | 18 | 19 | 19 | 23 | | 1,9 |
| 47. Nozu, A., Hamano, S., Tomokiyo, A., Hasegawa, D., Sugii, H., Yoshida, S., Mitarai, H., Taniguchi, S., Wada, N., & Maeda, H. (2019). Senescence and odontoblastic differentiation of dental pulp cells. *Journal of Cellular Physiology*, *234*(1), 849–859. | 18 | 19 | 21 | 21 | | 3,9 |
| 48. Zhai, Y., Wei, R., Liu, J., Wang, H., Cai, W., Zhao, M., Hu, Y., Wang, S., Yang, T., Liu, X., Yang, J., & Liu, S. (2017). Drug-induced premature senescence model in human dental follicle stem cells. *Oncotarget*, *8*(5), 7276–7293. | 17 | 18 | 18 | 25 | | 2,7 |
| 49. Kim, Y. G., Lee, S. M., Bae, S., Park, T., Kim, H., Jang, Y., Moon, K., Kim, H., Lee, K., Park, J., Byun, J.-S., & Kim, D.-Y. (2021). Effect of Aging on Homeostasis in the Soft Tissue of the Periodontium: A Narrative Review. *Journal of Personalized Medicine*, *11*(1), 58-73. | 11 | 13 | 16 | 27 | | 5,5 |
| 50. Hiratsuka, K., Kamino, Y., Nagata, T., Takahashi, Y., Asai, S., Ishikawa, K., & Abiko, Y. (2002). Microarray Analysis of Gene Expression Changes in Aging in Mouse Submandibular Gland. *Journal of Dental Research*, *81*(10), 679–682. | 8 | 10 | 15 | 22 | | 0,6 |

**Supplementary table 4.** Citation analysis of the top 50 most cited articles related to senescence and dentistry over the years (1999-2026).
