# Supplementary material for: Cellular senescence in the dental pulp and its implications for endodontics: a scoping review
Source: Clin Oral Investig. 2026 Mar 31;30(4):161. doi: 10.1007/s00784-026-06822-x (PMC13035753; doi:10.1007/s00784-026-06822-x)
Supplement: Supplementary file 4 — Supplementary Material 4 (DOCX 22.3 KB) [file 784_2026_6822_MOESM4_ESM.docx]

| Reference | Reason for exclusion |
| --- | --- |
| Galler, K. M., Schweikl, H., Thonemann, B., D'Souza, R. N., & Schmalz, G. (2006). Human pulp-derived cells immortalized with Simian Virus 40 T-antigen. *European journal of oral sciences*, *114*(2), 138–146. https://doi.org/10.1111/j.1600-0722.2006.00327.x | 3 |
| Schäfer, J., Kleszczynski, K., & Schäfer, E. (2025). Melatonin and the Dental Pulp: A Scoping Review. *International endodontic journal*, 10.1111/iej.70081. Advance online publication. https://doi.org/10.1111/iej.70081 | 8 |
| Ahmad, P., Estrin, N., Farshidfar, N., Zhang, Y., & Miron, R. J. (2025). Mechanistic insights into dental stem cells-derived exosomes in regenerative endodontics. *International endodontic journal*, *58*(9), 1384–1407. https://doi.org/10.1111/iej.14269 | 8 |
| Zhang, P., Cui, Y., Li, Z., Liu, L., Liu, X., Ding, X., & Ding, G. (2025). Senescence of dental pulp stem cells: phenotypes, underlying mechanisms and regulatory molecules. Human cell, 38(5), 127. https://doi.org/10.1007/s13577-025-01259-y | 8 |
| Brito, K. N. L., & Trentin, A. G. (2025). Role of mesenchymal stromal cell secretome on recovery from cellular senescence: an overview. *Cytotherapy*, *27*(4), 422–437. https://doi.org/10.1016/j.jcyt.2024.11.014 | 8 |
| Morita, M., Damle, E. B., Shinohara, I., Murayama, M., Susuki, Y., Gao, Q., Ma, C., Chow, S. K., & Goodman, S. B. (2025). Targeting cellular senescence in progenitor cells as a strategy to enhance bone regeneration by cell therapies: a systematic review of pre-clinical investigations. *Stem cell research & therapy*, *16*(1), 669. https://doi.org/10.1186/s13287-025-04767-8 | 8 |
| Huang, W., Hickson, L. J., Eirin, A., Kirkland, J. L., & Lerman, L. O. (2022). Cellular senescence: the good, the bad and the unknown. *Nature reviews. Nephrology*, *18*(10), 611–627. https://doi.org/10.1038/s41581-022-00601-z | 1 |
| De, D., Karmakar, P., & Bhattacharya, D. (2021). Stem Cell Aging and Regenerative Medicine. *Advances in experimental medicine and biology*, *1326*, 11–37. https://doi.org/10.1007/5584_2020_577 | 8 |
| Cao, Limin & Long, Xuan & Liang, Long & Zhang, Jushan & Tuerhong, Amanguli & Zhu, Shuli & Xie, Shuanshuan & Song, Xiaolian & Wang, Changhui. (2023). Dental Pulp Stem Cell-Derived Exosomes Inhibit Senescence-Induced Chronic Obstructive Pulmonary Disease Through the Nuclear Factor Kappa B Signaling Pathway. Journal of Biomedical Nanotechnology. 19. 658-666. 10.1166/jbn.2023.3556. | 2 |
| Couve, E., Osorio, R., & Schmachtenberg, O. (2013). The amazing odontoblast: activity, autophagy, and aging. Journal of dental research, 92(9), 765–772. https://doi.org/10.1177/0022034513495874 | 8 |
| Wang, Y., Jin, S., Guo, Y., Zhu, L., Lu, Y., Li, J., Heng, B. C., Liu, Y., & Deng, X. (2025). Cordycepin-Loaded Dental Pulp Stem Cell-Derived Exosomes Promote Aged Bone Repair by Rejuvenating Senescent Mesenchymal Stem Cells and Endothelial Cells. *Advanced healthcare materials*, *14*(2), e2402909. https://doi.org/10.1002/adhm.202402909 | 1 |
| Kok, Z. Y., Alaidaroos, N. Y. A., Alraies, A., Colombo, J. S., Davies, L. C., Waddington, R. J., Sloan, A. J., & Moseley, R. (2022). Dental Pulp Stem Cell Heterogeneity: Finding Superior Quality "Needles" in a Dental Pulpal "Haystack" for Regenerative Medicine-Based Applications. *Stem cells international*, *2022*, 9127074. https://doi.org/10.1155/2022/9127074 | 8 |
| Havelek, R., Soukup, T., Ćmielová, J., Seifrtová, M., Suchánek, J., Vávrová, J., Mokrý, J., Muthná, D., & Řezáčová, M. (2013). Ionizing radiation induces senescence and differentiation of human dental pulp stem cells. *Folia biologica*, *59*(5), 188–197. https://doi.org/10.14712/fb2013059050188 | 6 |
| Gao, H., Xing, D., Wu, M., Hu, Y., He, J., Chen, S., Zhang, G., Yao, F., Ma, P., & Xue, W. (2025). Injectable DAT-ALG Hydrogel Mitigates Senescence of Loaded DPMSCs and Boosts Healing of Perianal Fistulas in Crohn's Disease. *ACS biomaterials science & engineering*, *11*(2), 1171–1183. https://doi.org/10.1021/acsbiomaterials.4c02043 | 6 |
| Iezzi, I., Pagella, P., Mattioli-Belmonte, M., & Mitsiadis, T. A. (2019). The effects of ageing on dental pulp stem cells, the tooth longevity elixir. *European cells & materials*, *37*, 175–185. https://doi.org/10.22203/eCM.v037a11 | 8 |
| Egbuniwe, O., Idowu, B. D., Funes, J. M., Grant, A. D., Renton, T., & Di Silvio, L. (2011). P16/p53 expression and telomerase activity in immortalized human dental pulp cells. *Cell cycle (Georgetown, Tex.)*, *10*(22), 3912–3919. https://doi.org/10.4161/cc.10.22.18093 | 3 |
| Seifrtova, M., Havelek, R., Soukup, T., Filipova, A., Mokry, J., & Rezacova, M. (2013). Mitoxantrone ability to induce premature senescence in human dental pulp stem cells and human dermal fibroblasts. *Journal of physiology and pharmacology : an official journal of the Polish Physiological Society*, *64*(2), 255–266. | 6 |
| de Farias, J. O., & Rezende, T. M. B. (2023). Dental pulp and apical papilla cells senescence: causes, consequences, and prevention. *Biogerontology*, *24*(4), 533–539. https://doi.org/10.1007/s10522-023-10029-y | 8 |
| Maeda H. (2020). Aging and Senescence of Dental Pulp and Hard Tissues of the Tooth. *Frontiers in cell and developmental biology*, *8*, 605996. https://doi.org/10.3389/fcell.2020.605996 | 8 |
| Egbuniwe, O., Grant, A. D., Renton, T., & Di Silvio, L. (2013). Phenotype-independent effects of retroviral transduction in human dental pulp stem cells. *Macromolecular bioscience*, *13*(7), 851–859. https://doi.org/10.1002/mabi.201300020 | 3 |
| Morsczeck C. (2019). Cellular senescence in dental pulp stem cells. *Archives of oral biology*, *99*, 150–155. https://doi.org/10.1016/j.archoralbio.2019.01.012 | 8 |
| Mas-Bargues, C., Inglés, M., Gimeno-Mallench, L., Sanz-Ros, J., Viña-Almunia, J., Dromant, M., Borrás, C., & Viña, J. (2017). The importance of culturing primary cells under physiological conditions: Proliferation, senescence, pluripotency. Free Radical Biology and Medicine, 108(Suppl. 1), S43. https://doi.org/10.1016/j.freeradbiomed.2017.04.161 | 8 |
| Borrás, C., Inglés, M., Villalba, J. M., & Viña, J. (2015). Oxygen concentration in oxidative stress and replicative senescence in dental pulp stem cells. Free Radical Biology and Medicine, 86(Suppl. 1), P41. https://doi.org/10.1016/j.freeradbiomed.2015.07.116 | 8 |
| Martín-Piedra, M. A., Viñuela, J. M., Oliveira, A. C., Garzón, I., Alfonso, C., Rodríguez, I. A., & Sánchez-Quevedo, M. C. (2012). Selection of dental pulp stem cell populations as a cell source for use in tissue engineering of the skin and oral mucosa. *Tissue Engineering Part A*, 18(Suppl. 1), P29. https://doi.org/10.1002/term.1586 | 8 |
| Li, Y. L., Yang, J. Y., & Xu, R. (2020). *Shanghai kou qiang yi xue = Shanghai journal of stomatology*, *29*(5), 466–470. | 7 |
| Zou, X., Zhuang, H., Yue, L., & Gao, X. (2014). *Zhonghua kou qiang yi xue za zhi = Zhonghua kouqiang yixue zazhi = Chinese journal of stomatology*, *49*(4), 210–215. | 7 |
| Liu, J., Liu, Z., Wang, C., Yu, F., Cai, W., Lu, X., Wei, R., Zhao, S., Zhao, Y., & Liu, S. (2017). Tranylcypromine attenuates senescence in dental pulp stem cells. Letters in Drug Design & Discovery, 14(7), 819–826. https://doi.org/10.2174/1570180814666161229165048 | 7 |
| Wang, G. Y., Liao, L., & Tian, W. D. (2024). *Zhonghua kou qiang yi xue za zhi = Zhonghua kouqiang yixue zazhi = Chinese journal of stomatology*, *59*(5), 444–452. https://doi.org/10.3760/cma.j.cn112144-20240119-00030 | 7 |
| Li, X., Feng, L., Zhang, C., Wang, J., Wang, S., & Hu, L. (2022). Insulin-like growth factor binding proteins 7 prevents dental pulp-derived mesenchymal stem cell senescence via metabolic downregulation of p21. *Science China. Life sciences*, *65*(11), 2218–2232. https://doi.org/10.1007/s11427-021-2096-0 | 7 |
| Gupta, A. A., Kheur, S., Varadarajan, S., Rajkumar, C., & Patil, V. R. (2022). In-vitro analysis on the potential use of dental pulp mesenchymal stem cells on arecoline-induced oral epithelial cells. *Medical oncology (Northwood, London, England)*, *39*(5), 77. https://doi.org/10.1007/s12032-022-01673-4 | 7 |
| **Li, D., Liu, H., Wang, Y., Chen, Z., Zhang, X., & Li, W. (2024). Effect of WW-domain transcription regulator 1 on aging regulation of human dental pulp stem cells.** Chinese Journal of Stomatology**, 59(12), 1240–1247. https://doi.org/10.3760/cma.j.cn112144-20240521-00212** | 7 |
| Hou, H., Qiu, Z., Che, J., Li, Y., Sun, J., Zhang, W., Ma, J., Zhang, S., Li, M., Niu, Y., & He, L. (2025). Effects of simulated microgravity on dental pulp stem cell stemness. *Journal of Molecular Histology*, 56(2), 1–??. https://doi.org/10.1007/s10735-025-10377-8 | 7 |
| Ma, X., Liu, H., Zheng, Y., Dai, Y., Lingling, E., Zhang, R., & Zhang, S. (2023). Genome-Wide Screening of Differentially Expressed Genes and their Potential Associations with Aging Dental Pulp Stem Cells. *Combinatorial chemistry & high throughput screening*, *26*(7), 1337–1350. https://doi.org/10.2174/1386207325666220705120904 | 7 |
| Li, X. Y., Zhang, L., & Sun, Y. (2025). *Zhonghua kou qiang yi xue za zhi = Zhonghua kouqiang yixue zazhi = Chinese journal of stomatology*, *60*(1), 43–53. https://doi.org/10.3760/cma.j.cn112144-20240926-00360 | 7 |

**Supplementary table 2.** Excluded articles and reasons for exclusion (n=34).

**Exclusion Criteria**

1. Studies evaluating cellular senescence exclusively in non-dental tissues or organs, without relevance to oral biology or dentistry.

2. Studies focusing on senescence in systemic diseases or conditions (e.g., cancer, neurodegenerative diseases, metabolic disorders) without a clear dental, oral, or endodontic context.

3. Studies using immortalized or cancer cell lines with no translational relevance to dental tissues, unless explicitly used to model senescence-related mechanisms applicable to dentistry.

4. Studies that do not evaluate, describe, or discuss cellular senescence or senescence-associated phenomena, even if related to aging or dental materials.

5. Articles in which the senescent state is neither experimentally assessed nor conceptually addressed.

6. Publications focusing solely on molecular or genetic pathways of senescence without contextualization to dental tissues, pulp biology, tissue repair, or endodontic implications.

7. Studies for which the full text was unavailable or inaccessible.

8. Secondary literature and non-original research, including narrative reviews, systematic reviews, meta-analyses, scoping reviews, bibliometric analyses, editorials, letters, opinions, short communications, protocols, conference abstracts, posters, and case reports.
