# Supplementary material for: Cellular senescence in the dental pulp and its implications for endodontics: a scoping review
Source: Clin Oral Investig. 2026 Mar 31;30(4):161. doi: 10.1007/s00784-026-06822-x (PMC13035753; doi:10.1007/s00784-026-06822-x)
Supplement: Supplementary file 5 — Supplementary Material 5 (DOCX 81.5 KB) [file 784_2026_6822_MOESM5_ESM.docx]

| Reference | Study Design | Cell  Or Tissue | Main Objective | Senescence Induction Method | Key Senescence Markers Assessed | Key Findings | Implications for Endodontics | Limitations |
| --- | --- | --- | --- | --- | --- | --- | --- | --- |
| Dhok M et al. 2024 | *In vitro* | hDPSCs | Investigate effect of C. asiatica on DPSC senescence & differentiation | Long-term culture (passaging) | SA-β-gal, morphology | C. asiatica extract reduced senescence, inhibited adipogenesis, promoted osteogenesis | Potential for developing novel endodontic regenerative medicaments; anti-aging strategies | *In vitro* study, requires *in vivo* validation; specific bioactive compounds not isolated |
| Tong Z et al. 2025 | Observational & *In vitro* | DPCs, DPFs | Identify novel markers & mechanisms in aged dental pulp via scRNA-seq | Natural aging, H2O2 exposure *in vitro* | SA-β-gal, SASP genes (e.g., IGFBP7), γ-H2AX | IGFBP7 highly expressed in aged pulp; rIGFBP7 reduced H2O2-induced senescence in DPFs | IGFBP7 as a potential therapeutic target to modulate pulp aging and senescence | scRNA-seq findings require functional validation; clinical relevance to be determined |
| Bhandi S et al. 2021 | *In vitro* | SHEDs, yDPSCs, oDPSCs | Compare secretome profiles of dental stem cells from different age groups | Natural aging | Secretome profile (cytokines, growth factors), differentiation capacity | oDPSCs secrete more pro-inflammatory cytokines & fewer growth factors; lower osteo/chondrogenic potential | Aged patient DPSCs may have reduced regenerative capacity; suggests need for age-stratified therapies | *In vitro* secretome analysis; direct senescence markers not extensively assessed |
| Morsczeck C et al. 2018 | *In vitro* | DFCs | Investigate role of P16 in DFC senescence & osteogenic differentiation | Long-term culture (passaging) | SA-β-gal, P16, P21, CDKs, telomere length | P16-dependent pathway drives senescence in DFCs; P16 silencing reduced β-gal+ cells but did not restore osteogenic potential | Highlights P16's role in senescence of dental stem cells; potential target for maintaining stemness | Findings specific to DFCs; effect on differentiation not fully restored by P16 knockdown |
| Xu K et al. 2016 | *In vitro* | hDPSCs | Investigate effect of H2O2-induced oxidative stress on DPSC senescence | H2O2 exposure | SA-β-gal, p16, SIRT1, proliferation, morphology | H2O2 induced senescence in DPSCs, increased p16, decreased SIRT1 | Highlights impact of oxidative stress (common in periapical lesions) on DPSC function; suggests antioxidant strategies | Acute, high-dose H2O2 model may not fully mimic chronic inflammatory stress *in vivo* |
| Chen et al. 2024 | *In vitro* & *In vivo* | Human Dental Pulp Stem Cells (HDPSCs) | To investigate the role of ILK/AKT/mTOR/STAT1 pathway in HDPSC senescence and identify potential rejuvenation strategies | Age-related (donors aged 16–70 years) and pharmacological inhibition (OSU-T315, Rapamycin) | SA-β-gal, p21, p53, p16, IL-6, IL-8, MMP13, ILK, mTOR, STAT1, STAT3, Akt phosphorylation | HDPSCs show age-dependent decline in proliferation, migration, and osteogenic differentiation. ILK/AKT/mTOR/STAT1 pathway is upregulated with aging. Inhibition of ILK or mTOR reduces senescence markers and restores osteogenic potential. | Targeting senescence pathways (e.g., with ILK inhibitors) could rejuvenate aged DPSCs, improving their regenerative potential in regenerative endodontics and pulp regeneration. | *In vitro* and animal model only; clinical translation requires further validation. Heterogeneity in donor samples. Long-term effects of inhibitors unknown. |
| Lee et al. 2024 | *In vitro* & *In vivo* | Odontoblasts, human dental pulp cells (hDPCs), mouse dental pulp | To investigate the role of Cpne7 in dental pulp aging, oxidative stress, DNA damage, and senescence | Genetic knockout (Cpne7 KO), oxidative stress (H₂O₂), natural aging in mice | SA-β-gal, γ-H2AX, p21, ROS levels, TUNEL (apoptosis), DSP, DMP-1 | Cpne7 deficiency leads to premature pulp aging, increased oxidative stress, DNA damage accumulation, and pathological dentin formation. Cpne7 peptide rescues senescence and promotes physiological dentin repair. | Cpne7 or its derived peptide could be used to delay pulp aging, enhance dentin repair, and potentially treat age-related dentin hypersensitivity or caries progression. | Mouse model may not fully replicate human pulp aging. Incisors showed compensatory mechanisms, indicating complexity. Long-term safety and efficacy of peptide therapy in humans unknown. |
| Iezzi et al. 2019 | *In vitro* | Human Dental Pulp Stem Cells (hDPSCs) | To investigate age-related changes in DPSC morphology, proliferation, and differentiation potential | Age-related (donors: 20–64 years, grouped by age) | SA-β-gal, p16INK4a, telomere length, stemness genes (Sox2, Oct4, Nanog, Klf4), differentiation markers (DMP1, DSPP, nestin, β-tubulin III) | Older hDPSCs showed increased senescence markers, reduced proliferation, telomere shortening, and impaired osteogenic/odontogenic/neurogenic differentiation. Spontaneous odontogenic potential persists but mineralization is age-dependent. | Age-related decline in DPSC function should be considered in regenerative endodontic strategies. Older patients may have reduced regenerative capacity; donor age selection could optimize outcomes. | *In vitro* study only; no *in vivo* validation. Limited sample size and age range. Mechanisms of differentiation impairment not fully explored. |
| Dong et al. 2021 | *In vivo* | Submandibular gland cells (mouse), hDPSC-derived small extracellular vesicles (sEV) | To evaluate the therapeutic effect of hDPSC-sEV on irradiation-induced senescence in salivary glands | Irradiation (25 Gy) in mouse submandibular glands | SA-β-gal, pH2A.X, p16INK4a, p19Arf, p21, SASP factors (IL-6, MMP3, PAI-1, NF-κB, TGF-β), AQP5, SOD activity | Irradiation induced senescence in ductal epithelial cells; hDPSC-sEV reduced senescence markers, SASP, and oxidative stress, and preserved AQP5 expression. sEV protected ductal cells from senescence. | hDPSC-sEV could be a cell-free therapy to mitigate senescence in irradiated tissues, potentially applicable to protect dental pulp from radiotherapy-induced damage or inflammatory senescence. | Study focused on salivary glands, not dental pulp directly. Long-term effects and mechanisms of sEV action not fully elucidated. |
| Ok et al., 2020 | *In vitro* | Human dental pulp cells (hDPCs) | To investigate the role of visfatin in senescence of human dental pulp cells | Exogenous visfatin treatment; H₂O₂-induced oxidative stress | SA-β-galactosidase, p21, p53, γH2AX (telomere damage), SASP factors (IL-1β, IL-8, COX-2), NF-κB activation | Visfatin promotes hDPC senescence via NADPH consumption, oxidative stress, telomere damage, and SASP upregulation. FK866 (visfatin inhibitor) reverses these effects. | Visfatin may be a therapeutic target to delay pulp aging and reduce inflammation in endodontic conditions. FK866 could be explored as a protective agent in pulp therapy. | *In vitro* study; lacks *in vivo* validation; immortalized cell line may not fully represent primary pulp cells; clinical relevance not yet tested. |
| Xu et al., 2024 | *In vitro* | Human dental pulp stem cells (hDPSCs) | To investigate effects of long-term *in vitro* culture on hDPSC senescence and differentiation potential | Replicative senescence induced by prolonged *in vitro* passaging (up to P12) | SA-β-galactosidase, surface markers (CD73, CD90, CD105), osteogenic/lipogenic differentiation capacity, transcriptomic changes (RNA-seq) | Prolonged passaging induces senescence, reduces proliferation and differentiation, alters surface marker expression, and upregulates genes (CFH, WNT16, HSD17B2, IDI1, COL5A3) linked to senescence. | Highlights the importance of optimizing *in vitro* expansion protocols for hDPSC-based regenerative endodontics to avoid loss of stemness and function due to culture-induced senescence. | *In vitro* model only; no functional validation of identified genes (e.g., knockdown); limited sample size; clinical translation not addressed. |
| He et al., 2022 | *In vitro* | Human dental pulp stem cells (hDPSCs) | To elucidate the role of the ROR2/MSX2/NSUN2 axis in hDPSC senescence | Replicative senescence (long-term culture), ROR2 knockdown | SA-β-galactosidase, p21, p53, CDK4, NSUN2, MSX2, SASP factors (IL-8, IGFBP-2, GM-CSF) | ROR2 downregulation promotes senescence via MSX2 upregulation and NSUN2-mediated m5C methylation of p21 mRNA. ROR2 overexpression or MSX2/NSUN2 inhibition rescues senescence. | The ROR2/MSX2/NSUN2 axis is a potential target for modulating hDPSC senescence to improve stem cell-based regenerative outcomes in endodontics. | *In vitro* focus; complex signaling pathway; requires *in vivo* validation; therapeutic targeting not explored in clinical setting. |
| Zhang et al., 2024 | *In vitro* | Human Dental Pulp Stem Cells (DPSCs) | To investigate whether melatonin attenuates DPSC senescence due to *in vitro* expansion via inhibiting MMP3. | Long-term *in vitro* passaging (P2, P7, P12). | SA-β-gal, P53, P21, P16, MMP3, SASP factors (IL-6, MMP3). | Melatonin (0.1 µM) reduced senescence, improved osteogenic potential, and downregulated MMP3 and senescence markers. MMP3 overexpression reversed melatonin's protective effects. | Melatonin could be used during DPSC expansion for regenerative endodontic procedures to maintain stem cell quality and functionality. | *In vitro* study only; no *in vivo* validation. Mechanisms were identified bioinformatically but not fully validated experimentally. |
| de Farias et al., 2024 | *In vitro* | Human dental pulp cells (non-specified, likely heterogeneous population) | To evaluate morphological, proliferative, migratory, and immunomodulatory changes in senescent human pulp cells. | Chemical induction with 1 µL/mL doxorubicin for 24 hours. | SA-β-gal, cell morphology (size, extensions), proliferation rate, migration capacity, cytokine expression (TNF-α, IL-6, IL-10, TGF-β1). | Senescent cells showed enlarged morphology, fewer extensions, reduced proliferation and migration, increased pro-inflammatory (TNF-α, IL-6) and decreased anti-inflammatory (IL-10) cytokine expression. TGF-β1 expression was unchanged. | Senescence creates a pro-inflammatory, less reparative pulp environment, which may compromise the success of vital pulp therapies and conservative endodontic treatments by impairing the tissue's innate repair capacity. | Use of a chemotherapeutic agent (doxorubicin) may not fully mimic physiological senescence. Study focused on a heterogeneous pulp cell population, not specifically stem/progenitor cells. |
| Liu et al., 2025 | *In vitro* | Human Dental Pulp Stem Cells (DPSCs) | To explore the influence and mechanism of Pleiotrophin (PTN) on DPSCs under H₂O₂-induced oxidative stress. | Chemical induction with 100 µM H₂O₂ for 4 hours. | SA-β-gal, P53, P21, P16, intracellular ROS, antioxidant capacity (Nrf2, HO-1), osteogenic markers (RUNX2, BSP, DSPP). | PTN (50 pg/mL) pretreatment reduced senescence markers, decreased ROS, increased antioxidant capacity (via Nrf2/HO-1), and rescued osteogenic potential impaired by H₂O₂. PTN binds Nrf2, promotes its nuclear translocation via the PI3K/AKT pathway. | PTN could be a therapeutic agent to protect DPSCs from oxidative stress during regenerative procedures, potentially improving outcomes in inflamed or aged pulp tissues. | *In vitro* model using acute H₂O₂ exposure. The clinical translatability and optimal delivery method for PTN require further investigation. |
| Liang et al., 2020 | *In vitro* | Human Dental Pulp Stem Cells (DPSCs) from young (22-26 yrs) and elderly (62-75 yrs) donors | To identify senescence-associated protein changes in DPSCs and investigate the role of FAM96B in senescence, osteogenic differentiation, and proliferation. | Comparative analysis of DPSCs from young vs. elderly donors (natural aging model). Proteomic analysis. Functional assays via FAM96B knockdown/overexpression. | SA-β-gal, TERT activity, P53, P16, ALP activity, mineralization (Alizarin Red), osteogenic gene expression (DSPP, OCN), proliferation (CFSE). Proteomics identified 144 differentially expressed proteins (75 up, 69 down) in aged DPSCs. | FAM96B expression was higher in young DPSCs. Its knockdown increased senescence (↑SA-β-gal, ↑P53/P16, ↓TERT) and impaired osteogenesis/proliferation. Overexpression had the opposite effect, suppressing senescence and enhancing osteogenic differentiation and proliferation. | FAM96B is a key regulator of DPSC aging. Targeting FAM96B could be a strategy to rejuvenate aged DPSCs, potentially enhancing the efficacy of regenerative endodontic therapies in elderly patients. | The study is correlative and *in vitro*; the exact molecular mechanism by which FAM96B regulates senescence (e.g., interaction partners, signaling pathways) was not fully elucidated. Clinical relevance requires *in vivo* validation. |
| Nozu et al., 2018 | *In vitro* | Human dental pulp cells (HDPCs) isolated from healthy premolars of young adults (21-24 years old) | To clarify how TNF-α affects the odontoblastic differentiation of dental pulp cells during aging, focusing on the role of cellular senescence. | Long-term serial passaging until cells reached proliferation plateau (high population doublings, PD28). | Senescence-associated β-galactosidase (SA-β-gal) activity; mRNA expression of p16, p21, and p53. | Senescent HDPCs (sHDPCs) showed upregulated senescence markers and preserved odontoblastic differentiation capacity. TNF-α promoted odontoblastic differentiation in sHDPCs but inhibited it in young HDPCs (yHDPCs). This differential effect was mediated by increased expression of TNF receptor 1 (TNFR1) in sHDPCs. | Age-related changes in pulp cell responses to inflammatory mediators like TNF-α may influence reparative dentin formation and pulp calcification (e.g., denticles, pulp stones). Understanding senescence-associated signaling could inform new strategies for managing aged or inflamed pulp tissue in conservative endodontic therapies. | *In vitro* model using replicative senescence from long-term culture, which may not fully replicate the *in vivo* aging microenvironment. Limited to three HDPC lines from young donors, potentially not representative of aged individuals or *in vivo* pathophysiological conditions. The study focused on TNF-α/TNFR1; other SASP factors and signaling pathways in pulp senescence were not explored. |
| Wang et al., 2025 | *In vitro* & *In vivo* | Human dental pulp stem cells (HDPSCs) isolated from donors aged 16-70 years | To investigate the role of the nuclear pore protein NUP62 in HDPSC senescence and its regulatory mechanism via epigenetic reprogramming. | Natural aging (comparison of cells from young vs. old donors) and replicative senescence induced by long-term passaging. | SA-β-gal activity; protein and mRNA levels of p16, p21, p53; γH2AX (DNA damage); expression of SASP factors (IL-6, IL-8, MMP13). | NUP62 expression decreased with age in HDPSCs. Overexpression of NUP62 alleviated senescence phenotypes, enhanced multilineage differentiation, and promoted bone regeneration *in vivo*. The mechanism involves NUP62 facilitating nuclear transport of E2F1, which upregulates the histone methyltransferase NSD2, leading to increased H3K36me2/3 modifications on anti-aging genes (HMGA1, HMGA2, SIRT6). | Targeting the NUP62-NSD2 epigenetic axis could be a novel strategy to rejuvenate aged dental pulp stem cells, enhancing their regenerative capacity for pulp repair, dentin regeneration, and potential use in regenerative endodontic procedures (REPs). | The study primarily establishes a correlation and mechanism *in vitro* and in animal models; clinical translatability requires further validation. The specific role of NUP62 in the context of pulp inflammation (pulpitis) was not addressed. The long-term safety and efficacy of modulating NUP62 in human therapies are unknown. |
| Alraies et al., 2017 | *In vitro* | Clonal populations of human dental pulp stem cells (DPSCs) isolated from young adults (18-30 years) via fibronectin adhesion assay | To assess the heterogeneity in proliferative and regenerative potential among clonal DPSC populations and investigate the role of telomere length and senescence in these differences. | Replicative senescence induced by prolonged *in vitro* expansion until proliferation plateau (PD <0.5/week). | Cumulative population doublings (PDs); SA-β-gal activity; cell morphology/size; mRNA expression of p53, p21, p16; telomere length (TRF assay); expression of stem cell markers (CD73, CD90, CD105, CD271). | High proliferative capacity DPSC clones (e.g., A3, >80 PDs) had longer telomeres (~18.9 kb), maintained stem cell markers longer, and were multipotent (osteogenic, chondrogenic, adipogenic). Low proliferative clones (<40 PDs) had shorter telomeres (5-13 kb), expressed CD271, entered senescence earlier, and were mostly unipotent (osteogenic only). Prolonged expansion of high-capacity clones led to telomere shortening, senescence, and loss of multipotency. | Identifying DPSC subpopulations with high proliferative and regenerative potential (long telomeres, CD271-negative) could improve cell selection for regenerative endodontics. Understanding senescence-linked loss of potency informs limits of *in vitro* expansion for therapeutic use. CD271 may be a negative marker for selecting superior DPSCs. | The study is limited to a small number of clones (n=6) from three young donors, restricting generalizability. The findings are based on *in vitro* expansion; *in vivo* relevance and behavior within the native pulp niche are not confirmed. The role of hTERT was not detected, suggesting other telomere maintenance mechanisms exist but were not identified. |
| Ding & Ran, 2025 | *In vitro* | Human dental pulp stem cells (DPSCs) isolated from third molars of young donors (18-26 years) | To investigate the role of O-GlcNAcylation, specifically via O-GlcNAcase (OGA), in regulating DPSC senescence and mitophagy, focusing on the transcription factor KLF2. | Replicative senescence induced by long-term passaging (comparison of passage 7 (p7) vs. passage 15 (p15) cells). | SA-β-gal activity; TERT activity (ELISA); protein levels of p53, p21, p16; EdU assay (proliferation); mitophagy markers (Pink1, Parkin, Beclin-1, LC3B-II/I); O-GlcNAc levels; OGA and KLF2 expression. | OGA expression increased in senescent p15 DPSCs. Knockdown of OGA inhibited senescence, promoted proliferation, and enhanced mitophagy. OGA interacted with and suppressed O-GlcNAcylation of KLF2 at Ser177, reducing KLF2 stability. The anti-senescence and pro-mitophagy effects of OGA knockdown were reversed by simultaneous KLF2 knockdown. | Modulating the OGA/KLF2/O-GlcNAcylation axis could be a therapeutic strategy to delay DPSC replicative senescence, potentially improving the yield and quality of expanded DPSCs for use in regenerative endodontic procedures and pulp tissue engineering. | The study is conducted *in vitro* using a replicative senescence model from young donors; the relevance to *in vivo* aging or age-related pulp changes is not established. The translational potential of targeting OGA/KLF2 in a clinical setting requires extensive validation. The study does not explore the role of this pathway in inflammatory pulp conditions. |
| Li et al., 2025 | *In vitro* | Human Dental Pulp Stem Cells (DPSCs) | To explore the role of the m⁶A eraser FTO in DPSC senescence. | Replicative senescence (extended *in vitro* passaging, passages 6-12) and FTO knockdown (siRNA, inhibitor FB23-2). | β-galactosidase activity, p16, p53, γH2AX, ROS levels, cell cycle arrest (G0/G1), proliferation rate. | FTO expression decreases during DPSC senescence. FTO depletion accelerates senescence, increases ROS, and inhibits proliferation by upregulating NOLC1 via m⁶A-dependent mRNA stabilization. The FTO/NOLC1/p53 axis regulates senescence via inhibition of pre-rRNA synthesis and nucleolar stress. | Identifies FTO as a potential therapeutic target to delay DPSC senescence, which could improve the efficacy and scalability of DPSC-based regenerative endodontic therapies by maintaining stem cell vitality. | The study is *in vitro*; validation *in vivo* is needed. The m⁶A sites on NOLC1 mRNA were identified in HEK293 cells and require confirmation in DPSCs. The role of m⁶A reader proteins in this pathway was not explored. |
| Gu et al., 2016 | *In vitro* | Human Dental Pulp Stem Cells (HDPSCs) | To investigate the role of miR-152 in HDPSC senescence and its relationship with SIRT7. | Replicative senescence (serial passaging to Population Doubling 54). | SA-β-galactosidase activity, p21, p16, Ki67 (proliferation marker), cell viability. | miR-152 is upregulated in senescent HDPSCs. Overexpression of miR-152 induces senescence and inhibits proliferation by directly targeting and downregulating SIRT7. Inhibition of miR-152 or overexpression of SIRT7 rescues the senescent phenotype. | The miR-152/SIRT7 axis is a key regulator of HDPSC aging. Modulating this pathway (e.g., inhibiting miR-152 or enhancing SIRT7) could be a strategy to maintain HDPSC function, potentially improving outcomes in regenerative endodontic procedures like pulp capping. | The study is *in vitro*. The therapeutic potential of targeting miR-152/SIRT7 in a clinical or *in vivo* setting remains to be investigated. Mechanisms of miR-152 upregulation during aging were not explored. |
| Zhang et al., 2025 | *In vitro* | Human Dental Pulp Stem Cells (hDPSCs) from young (18-27y) and aged (60-70y) donors | To evaluate the anti-aging effects of Biodentine on hDPSCs and explore the underlying mechanism involving the Wnt/β-catenin pathway. | Natural aging (using cells isolated from elderly donors) and chemical inhibition of Wnt pathway (XAV939). | SA-β-galactosidase activity, p53, p21, p16, cell cycle analysis (S-phase fraction). | Biodentine extract (optimal at 0.2 mg/mL) promoted proliferation, odonto/osteogenic differentiation, and reduced senescence markers in aged hDPSCs. These anti-aging effects were mediated by activation of the Wnt/β-catenin pathway, as they were reversed by the inhibitor XAV939. | Biodentine, a clinically used pulp capping material, may rejuvenate aged dental pulp by counteracting cellular senescence via the Wnt/β-catenin pathway. This supports its use in vital pulp therapy for older patients to improve treatment success rates. | The study uses cell extracts rather than direct material contact. The specific bioactive component(s) in Biodentine responsible for the effect are unknown. *In vivo* validation and long-term clinical studies are needed. |
| Liu et al., 2023 | *In vivo* | Rat dental pulp cells (*in situ*) | To investigate the localization of senescent cells after dental cavity preparation (DCP) and the effect of senolytics on reparative dentin formation. | Mechanical stress from dental cavity preparation (DCP) without pulp exposure. | p21, p16 (immunohistochemistry), reparative dentin area (histomorphometry). | DCP induced senescent cell accumulation (p21+/p16+) primarily in the pulp horn. Senolytic treatment (dasatinib + quercetin) eliminated these cells and restored reparative dentin formation volume, similar to the effect of calcium hydroxide pulp capping. | Senescent cells induced by clinical procedures (e.g., cavity preparation) may impair pulp repair. Targeted removal of senescent cells using senolytics could be a novel therapeutic strategy to enhance reparative dentin formation and improve outcomes of vital pulp therapies. | The study is in rats; translation to humans is needed. The specific cell types that become senescent (odontoblasts, stem cells, etc.) were not identified. Long-term safety and efficacy of senolytics in the oral cavity are unknown. |
| Yang et al., 2021 | *In vitro* & *Ex vivo* | Human DPSCs from deciduous teeth (SHED), young (Y-DPSCs), and old (A-DPSCs) permanent teeth | To investigate the role of serine metabolism in DPSC aging and its regulation of p16 via DNA methylation. | Natural aging (comparison of cells from different age groups) and siRNA knockdown of PHGDH. | p16 expression (protein/mRNA), DNA methylation (5-mC enrichment at p16 promoter), proliferation (BrdU, Ki67), differentiation capacity. | Serine metabolism decreases with age in DPSCs. Reduced serine metabolism lowers S-adenosylmethionine (SAM) levels, leading to hypomethylation of the p16 promoter and increased p16 expression, driving senescence. Knockdown of PHGDH (serine synthesis enzyme) in young DPSCs recapitulates aging phenotypes. | Aging-related decline in serine metabolism is a key driver of DPSC senescence. Nutritional or pharmacological strategies to support serine metabolism or DNA methylation could potentially delay pulp aging and improve the success of regenerative endodontic treatments in older patients. | The study is correlative; direct causal proof *in vivo* is limited. The complexity of one-carbon metabolism and interactions with other aging pathways require further investigation. Clinical translatability of modulating serine metabolism needs evaluation. |
| da Silva et al., 2025 | *In vitro* | Primary human dental pulp cells (heterogeneous, mainly fibroblast-like) | To investigate how senescence influences the viability, morphology, migration, proliferation, and immune-inflammatory response of human dental pulp cells under inflammatory conditions. | Chemical induction using 500 µM doxorubicin for 24 hours. | Senescence-associated β-galactosidase (SA-β-gal) activity. | Senescent pulp cells showed reduced viability, proliferation, and migration; increased cell size and decreased cellular extensions; exacerbated pro-inflammatory profile (increased IDO, TNF-α, IL-6); and reduced TGF-β1 expression under inflammatory stimuli (LPS, IFN-γ). | Senescence impairs pulp regenerative capacity and immune balance, potentially compromising the success of conservative/regenerative endodontic treatments, especially in aged or inflamed pulps. Targeting senescence (senotherapeutics) could be a promising strategy. | Use of an acute chemical senescence model (doxorubicin) rather than replicative or age-related models. Heterogeneity of primary pulp cell cultures. *In vitro* study limits direct translation to clinical complexity. |
| Song et al., 2024 | *In vitro* | Human Dental Pulp Stem Cells (hDPSCs) | To develop a novel scanning ion conductance microscopy (SICM) method for simultaneously imaging extracellular membrane topography and surface charge distribution in aging cells. | Replicative senescence (long-term culture, ≥12 passages). | Surface charge density (via ΔI-SICM), SA-β-Gal activity, morphology (F-actin), p53/p21 expression, cell cycle, mitochondrial membrane potential (JC-1). | Senescent hDPSCs exhibited significantly more negative extracellular surface charge, altered morphology, increased SA-β-Gal activity, higher p53/p21 expression, cell cycle arrest (G1), and decreased mitochondrial membrane potential. | Provides a new tool (SICM) to detect biophysical changes (surface charge) in senescent pulp cells. Altered membrane properties in aging cells could affect cell signaling, repair capacity, and response to biomaterials in regenerative endodontics. | *In vitro* model may not fully mimic the complex *in vivo* pulp environment. Method is technically complex and may not be readily applicable in clinical settings. |
| Vongprommool et al., 2024 | *In vitro* | Human Dental Pulp Stem Cells (DPSCs) | To investigate changes in Alu element methylation levels during the replicative senescence of human DPSCs. | Replicative senescence (long-term culture until proliferation ceased). | Alu methylation levels (via qCOBRA-Alu), morphology, SA-β-Gal activity, doubling time. | Late-passage (senescent) DPSCs showed significant Alu hypomethylation, increased SA-β-Gal activity, altered morphology (larger, flatter cells), and longer doubling times. | Epigenetic alterations (Alu hypomethylation) are a feature of DPSC aging. This could impact genomic stability and differentiation potential, crucial for stem cell-based regenerative endodontic procedures. Senescence may be a limiting factor for *ex vivo* expansion of DPSCs for therapy. | Study does not establish a direct causal relationship between Alu hypomethylation and functional senescence. Findings are associative. Focus on one epigenetic marker; other pathways are not explored. |
| Feng et al., 2014 | *In vitro* | Human Dental Pulp Stem Cells (DPSCs) | To investigate whether repeated inflammatory stimulation with lipopolysaccharide (LPS) induces cellular senescence in DPSCs and to explore the underlying mechanisms. | Repeated LPS stimulation (3 or 6 times). | SA-β-Gal activity, morphology, cell proliferation (BrdU), cell cycle (flow cytometry), ROS, γ-H2A.X (DNA damage), p16INK4A, TLR4. | Repeated LPS stimulation induced DPSC senescence, characterized by enlarged/flattened morphology, increased SA-β-Gal activity, G0/G1 arrest, elevated ROS, DNA damage (γ-H2A.X), and upregulation of p16INK4A via TLR4 signaling. Knockdown of p16INK4A reversed senescent features. | Chronic inflammation (as in pulpitis) can drive DPSCs into a senescent state via the p16INK4A pathway, potentially compromising the pulp's intrinsic repair capacity. Targeting this pathway (e.g., anti-inflammatory strategies) might preserve DPSC function during endodontic therapy. | The LPS stimulation model may represent an acute/severe inflammatory challenge; relevance to low-grade chronic inflammation in pulp needs further study. *In vivo* validation is required. |
| Zayed et al., 2020 | *In vitro* | Canine Dental Pulp Stem Cells (DPSCs) | To investigate the effects of the uremic toxin p-Cresol (PC) on inducing senescence and age-related phenotypic changes in DPSCs. | Treatment with p-Cresol (PC) at various concentrations (100-500 μM). | SA-β-Gal activity, cell size/proliferation, senescence markers (p21, IL-1β, IL-8, p53, p16), apoptosis markers (Bax/Bcl-2), inflammation (IL-6), odontoblast differentiation markers (DSPP, DMP1, Osx, ALP activity, mineralization). | PC induced DPSC senescence, evidenced by reduced proliferation, enlarged morphology, increased SA-β-Gal activity, elevated p21, IL-1β, IL-8, p53, and p16 protein. It also promoted apoptosis, inflammation (IL-6), and severely inhibited odontoblast differentiation and mineralization. | Systemic factors (like uremic toxins in aging or disease) can induce pulp cell senescence, impairing the dentin-pulp complex's regenerative potential. This highlights the need to consider patient systemic health in endodontic prognosis and the potential for senolytic or anti-toxin adjunctive therapies. | Use of canine cells may limit direct translation to human biology. PC concentrations used may be supra-physiological. The study focuses on one toxin; the combined effect of multiple age-related factors is unknown. |
| Lyu et al., 2025 | *In vitro* | Human Dental Pulp Stem Cells (hDPSCs) | To explore molecular mechanism changes during replicative senescence of hDPSCs and investigate the potential role of pregnancy-specific glycoprotein 4 (PSG4) in modulating this process. | Replicative senescence (long-term culture, passages P4 to P12) and H2O2-induced senescence. | SA-β-Gal activity, colony formation, cell cycle analysis (flow cytometry), P16 protein, transcriptome sequencing (RNA-seq), PSG4 expression (qPCR, WB). | Senescent hDPSCs showed G0/G1 arrest, reduced proliferation, increased SA-β-Gal and P16. PSG4 expression was significantly upregulated during senescence. PSG4 knockdown alleviated senescence, while its overexpression exacerbated it, likely via cell cycle disruption. | Identifies PSG4 as a novel key regulator of hDPSC senescence. Targeting PSG4 could be a strategy to delay senescence during *in vitro* expansion of DPSCs for regenerative endodontic procedures, improving cell quality and therapeutic potential. | The specific molecular pathway by which PSG4 regulates the cell cycle and senescence remains unclear. Findings are based on *in vitro* models; *in vivo* validation is needed. |
| Qiao Yi et al., 2017 | *In vitro* | Human dental pulp stem cells (DPSCs) | To investigate age-related differences in proliferation, differentiation potential, and gene expression profiles of DPSCs. | Natural aging (comparison of DPSCs from young donors aged 12–25 years vs. old donors aged 60–70 years). | Proliferation rate, doubling time, ALP activity, mineralization (alizarin red), lipid accumulation (Oil Red O), LncRNA and mRNA expression profiles. | DPSCs from older donors showed reduced proliferation, osteogenic, and adipogenic potential, but chondrogenic potential was unaffected. Numerous LncRNAs and mRNAs were differentially expressed with age. NFYB, GTF2B, NR3C1 identified as core transcription factors. | Donor age affects DPSC functionality, which may impact the efficacy of DPSC-based regenerative endodontic therapies. Older patients may have less potent endogenous stem cells for pulp regeneration. | *In vitro* study; does not fully replicate the *in vivo* microenvironment. Small sample size (n=4 per group). Limited to DPSCs; other pulp cell types not examined. |
| Shi et al., 2024 | *In vitro* | SCAPs (Stem Cells from Apical Papilla) | To investigate how 3D culture inhibits replicative senescence in SCAPs and identify underlying mechanisms. | Replicative senescence via serial passaging in 2D vs. 3D methylcellulose culture. | SA-β-gal, P53, P21, P16 mRNA/protein, mitochondrial function (OCR, MMP, ROS), UQCRC2 expression. | 3D culture delays senescence by maintaining mitochondrial homeostasis via UQCRC2-mediated oxidative phosphorylation; knockdown of UQCRC2 accelerates senescence. | Suggests 3D culture as a strategy to maintain stemness and delay senescence in SCAPs for regenerative endodontics and pulp-dentin regeneration. | *In vitro* model; limited *in vivo* validation; mechanism primarily focused on mitochondrial pathway. |
| Li et al., 2012 | *In vitro* | Human Dental Pulp Cells (DPCs) | To determine whether autophagy is involved in dental pulp cell senescence. | Replicative senescence via long-term serial passaging. | SA-β-gal, LC3-II, Beclin 1, autophagic vacuoles (TEM). | Autophagic activity (LC3-II, Beclin 1, autophagic vacuoles) increases in senescent DPCs. | Highlights autophagy as a potential modulator of pulp aging; may inform strategies to mitigate senescence in pulp repair and regeneration. | Only *in vitro*; correlative findings; no interventional or mechanistic exploration; small sample size. |
| Macrin et al., 2019 | *In vitro* | Dental Pulp Stem Cells (DPSCs) | To identify early predictors (metabolic and molecular) of DPSC aging and variability in senescence rates among individuals. | Replicative senescence via serial passaging; comparison of rapid aging (RA) vs. slow aging (SA) DPSC lines. | SA-β-gal, CDKN2A (P16), CDKN1A (P21), metabolic flux (OCR, ECAR), BARX1, TGF-β pathway, cytoskeletal proteins. | BARX1 identified as a DPSC marker; RA-DPSCs show early metabolic signature (low glycolysis/fatty acid oxidation) and upregulated TGF-β/cytoskeletal pathways predictive of senescence. | Early metabolic profiling could help select DPSC populations with better longevity for clinical use; BARX1 as a potential purity marker for DPSCs. | *In vitro* model; donor variability; predictive signature requires validation in larger cohorts and *in vivo*. |
| Das et al., 2022 | *In vitro* | DPSCs and Umbilical Cord Stem Cells (UCSCs) | To compare proliferation capacity and replicative senescence between DPSCs and UCSCs. | Replicative senescence via serial passaging to P6. | SA-β-gal, intracellular ROS, vimentin, proliferation (MTT, PD), cytoskeleton (phalloidin). | DPSCs showed higher proliferation, lower ROS, and lower SA-β-gal activity than UCSCs at later passages, indicating lower replicative senescence. | Suggests DPSCs as a superior source for long-term expansion in regenerative endodontics due to slower senescence and better retention of proliferative capacity. | Only two passages compared (P2 vs. P6); limited molecular mechanistic insight; no differentiation potential assessed in senescence context. |
| Li et al., 2025 | *In vitro* | Human dental pulp stem cells (hDPSCs) | To evaluate the effects of Resveratrol on the senescence of hDPSCs and explore new therapeutic strategies. | Serial passaging (up to passage 9, P9) to create a replicative senescence model. | SA-β-gal activity, protein expression (P16, P21, P53), mRNA expression (P16, P21, P53). | Resveratrol delayed senescence, enhanced proliferation and osteogenic differentiation in senescent hDPSCs. It activated SIRT1, which upregulated PINK1/PGC-1α, enhancing mitochondrial autophagy and biogenesis. | Resveratrol could be a therapeutic agent to rejuvenate aged or senescent hDPSCs, potentially improving outcomes in regenerative endodontic procedures like pulp capping or revascularization. | Entirely *in vitro* study; lack of *in vivo* validation. Did not analyze senescence-associated secretory phenotype (SASP). P9 model may not fully replicate the complex *in vivo* aging microenvironment. |
| Zhang et al., 2025 | *In vitro* | Human dental pulp stem cells (DPSCs) | To identify the regulatory role of SIRT7 in DPSC senescence and investigate the underlying mechanism involving desuccinylation. | Replicative senescence induced by serial passaging (Population Doubling 54, PD54). | SA-β-gal activity, TERT activity, protein expression (p53, p21, p16, SIRT7). | SIRT7 expression decreases in senescent DPSCs. SIRT7 knockdown accelerates senescence, while its overexpression delays it. SIRT7 binds to ROCK1 and desuccinylates it at K520, regulating ROCK1 stability. The SIRT7/ROCK1 axis is key in DPSC senescence. | Targeting the SIRT7/ROCK1 axis could be a strategy to delay DPSC senescence, potentially improving the efficacy of DPSC-based regenerative endodontic therapies like pulp regeneration. | *In vitro* study using a replicative senescence model that may not fully mimic the complex *in vivo* aging microenvironment. Mechanisms were explored primarily through genetic manipulation. |
| Yaghoobi et al., 2020 | *In vitro* | Human dental pulp stem cells (DPSCs) from three donors | To evaluate and compare the ability of hydrogen peroxide, doxorubicin, and UV irradiation to induce senescence in DPSCs. | Stress-induced premature senescence using chemical (H₂O₂, doxorubicin) and physical (UV irradiation) stressors. | SA-β-gal activity, cell cycle analysis (flow cytometry), mRNA expression (p21, p16, BTG1, CCND1, etc.). | All three agents successfully induced senescence, characterized by SA-β-gal positivity, G1 cell cycle arrest, and increased p21 expression. UV irradiation (1 min) was particularly potent. Senescence proceeded via the p53-p21 pathway. | Highlights that common lab stressors (ROS, light, chemotherapeutics) can prematurely age DPSCs. For clinical cell expansion in regenerative endodontics, protocols must minimize these stressors to maintain DPSC potency. | Study focused on acute induction of senescence; long-term consequences and relevance to chronic *in vivo* aging were not assessed. Did not investigate rescue or reversal of the senescent phenotype. |
| Zayed et al., 2020 | *In vitro* & *In vivo* | Human periodontal ligament cells (HPDLCs) and *in vivo* model in aged dogs | To examine the effect of CCR3 antagonist (CCR3A) on cellular senescence, anti-inflammation, and rejuvenation to promote pulp regeneration in aged teeth. | Chemical induction using para-Cresol (p-Cresol; 500 µM for 72h) to induce senescence in HPDLCs. | Cell size, proliferation (PrestoBlue), mRNA expression (p21, IL-1β, CCL11). | CCR3A protected HPDLCs from p-Cresol-induced senescence, reducing cell size increase, restoring proliferation, and decreasing p21 and IL-1β. CCR3A also decreased CCL11 and increased immunomodulatory IDO. In aged dogs, CCR3A enhanced pulp regeneration and neurite extension when co-transplanted with MDPSCs. | CCR3A could be a therapeutic adjunct to counteract senescence and chronic inflammation in aged dental pulp, potentially improving outcomes of regenerative endodontic procedures in older patients. | *In vivo* study was performed in a canine model; human clinical relevance needs confirmation. The molecular mechanism linking CCR3A to senescence reversal was not fully elucidated. |
| Choi et al., 2012 | *In vitro* | Human dental pulp stem cells (DPSCs) | To investigate the effect of a cell-penetrating superoxide dismutase (LMWP-SOD1) conjugate on oxidative stress-induced senescence and differentiation in DPSCs. | Exposure to hydrogen peroxide (H₂O₂; 200 µM for 2 hours). | Cell morphology (enlargement/flattening), SA-β-gal activity, cell cycle analysis (G1 arrest), p53 and p21Cip1/WAF1 expression (mRNA and protein). | LMWP-SOD1 attenuated H₂O₂-induced senescence phenotypes: reduced SA-β-gal activity, restored morphology, suppressed p53/p21 pathway. It reversed H₂O₂-impaired osteogenic but not odontogenic differentiation. | Potential for antioxidant-based therapies to mitigate oxidative stress-induced DPSC senescence, potentially improving pulp regeneration in endodontic therapy. | *In vitro* study; limited to DPSCs; does not address other pulp cell types; LMWP-SOD1 did not restore odontogenic potential. |
| Mas-Bargues et al., 2017 | *In vitro* | Human dental pulp stem cells (hDPSCs) | To analyze the role of p16INK4a and BMI-1 in oxidative stress-induced premature senescence during long-term hDPSC culture. | Long-term *in vitro* culture at ambient (21%) vs. physiological (3%) oxygen tension. | SA-β-gal activity, cell morphology (enlargement/flattening), p16INK4a and p14ARF mRNA, BMI-1 protein, OSKM and TET1 mRNA expression. | Culture at 21% O2 induced oxidative stress, premature senescence (increased SA-β-gal, p16INK4a), and stemness loss. BMI-1 knockdown rescued SOX2/OCT4 expression. Physiological O2 (3%) delayed senescence and maintained stemness. | Highlights the critical importance of using physiological oxygen tension (low pO2) during DPSC expansion for clinical applications to delay senescence and preserve regenerative potential for pulp regeneration therapies. | *In vitro* study; long-term culture model may not fully replicate *in vivo* aging; mechanisms linking BMI-1 to stemness in this context require further elucidation. |
| Ou et al., 2018 | *In vitro* & *Ex vivo* | Human dental pulp cells (HDPCs) from young and old patients; primary HDPC cultures. | To elucidate the role of sclerostin in HDPC senescence and senescence-related impairment of proliferation and odontoblastic differentiation. | Serial subculture until replicative arrest; comparison of young vs. old dental pulp tissue; sclerostin overexpression/knockdown. | SA-β-gal activity, proliferation rate (CCK-8), p16, p53, p21 mRNA and protein, odontogenic markers (ALP, OCN, OPN, DSPP), Wnt/β-catenin pathway (p-β-catenin). | Sclerostin expression increased in aged pulp and senescent HDPCs. Sclerostin promoted senescence (increased SA-β-gal, p16/p53/p21) and impaired odontoblastic differentiation, potentially via inhibiting Wnt/β-catenin signaling. | Identifies sclerostin as a potential therapeutic target to delay HDPC senescence and preserve odontogenic capacity, which could improve outcomes in vital pulp therapy and pulp regeneration. | Primarily *in vitro* and *ex vivo*; causal role of sclerostin *in vivo* not established; exact mechanism linking sclerostin to p16/p53 pathways requires further investigation. |
| Zhang et al., 2023 | *In vitro* | Human dental pulp stem cells (DPSCs) | To investigate the effect of pleiotrophin (PTN) on senescent DPSCs and its role in attenuating senescence. | Replicative senescence model (long-term culture to passage 15); PTN knockdown via shRNA. | SA-β-gal activity, p16, p53, p21, TERT, telomerase activity, cell cycle regulators (Cyclin D, CDK4, CDK1, Cyclin B), osteogenic markers (ALP, OPN, RUNX2, OSX), p-p38 MAPK. | PTN knockdown accelerated senescence (increased SA-β-gal, p16; decreased TERT, telomerase). PTN recombinant protein (50 pg/ml) rescued senescence phenotypes, improved proliferation, restored osteogenic potential, and activated p38 MAPK signaling. | Suggests PTN as a potential therapeutic factor to delay DPSC senescence and enhance pulp-dentin regeneration efficacy, possibly improving outcomes in regenerative endodontic procedures. | *In vitro* study using a replicative senescence model; *in vivo* validation is needed; exact downstream mechanisms of PTN/p38 MAPK require further investigation. |
| Muthna et al., 2010 | *In vitro* | Adult human dental pulp stem cells (DPSCs) | To characterize the response of DPSC lines to ionizing radiation (IR). | Gamma irradiation (2–20 Gy) | SA-β-galactosidase, p16, p21, p53 (total and phosphorylated at Ser15 and Ser392) | IR induced permanent G2-phase cell cycle arrest and stress-induced premature senescence, not apoptosis. p53/p21 increased early; p16 and SA-β-gal activity increased from day 3. | DPSCs may enter senescence after radiotherapy (head/neck cancer), potentially compromising pulp repair and regeneration in endodontic therapy. | *In vitro* model; DPSCs from young donors only (impacted third molars); long-term clinical relevance not investigated. |
| Wang et al., 2015 | *In vitro* | Human dental pulp cells (HDPCs) from young (18-28y) and aging (65-75y) donors | To identify senescence-associated miRNAs in HDPCs and investigate the role of miR-433. | Natural aging (comparison between age groups); experimental modulation via lentiviral vectors (overexpression/knockdown of miR-433) | miRNA expression profiles (microarray, qRT-PCR), SA-β-gal (implied), GRB2, RAS (protein/mRNA), ALP activity, apoptosis assay | miR-433 was significantly upregulated in aging HDPCs. It negatively regulates GRB2 and the RAS-MAPK pathway, leading to reduced proliferation/mineralization and increased apoptosis. | Targeting miR-433 could be a strategy to enhance the repair and regeneration capacity of aging dental pulp, potentially improving outcomes in regenerative endodontics for older patients. | *In vitro* model; complex miRNA network interactions not fully explored; direct clinical translation requires further validation. |
| Zou et al., 2010 | *In vitro* | Human dental pulp cells | To evaluate whether the Notch signalling pathway is involved in the senescence of human dental pulp cells. | Pharmacological inhibition using DAPT (γ-secretase inhibitor) | Senescence-associated β-galactosidase (SA-β-gal) activity | DAPT inhibition of Notch signalling decreased cell proliferation and induced SA-β-gal positive staining. | Notch pathway inhibition may promote pulp cell senescence, potentially impairing pulp repair capacity and regenerative potential in endodontic therapy. | *In vitro* study using cells from young donors (third molars); long-term effects and *in vivo* relevance not examined. |
| Lee et al., 2013 | *In vitro* | Human dental pulp cells (HDPCs) | To investigate age-related changes in HDPCs, focusing on inflammation and dentinogenesis, and compare replicative senescence with stress-induced premature senescence (SIPS). | Replicative senescence (serial subculture up to PD25); SIPS induced by H₂O₂ (150 µmol/L) | SA-β-galactosidase activity, ROS formation, p53, p21, p-ERK1/2, c-myb, ICAM-1, VCAM-1, PPAR-γ, HO-1 | Replicative senescence and SIPS increased SA-β-gal activity and ROS, altered inflammatory/aging markers (↑ICAM-1, VCAM-1; ↓PPAR-γ, HO-1; ↑p53, p21, p-ERK, c-myb), and decreased odontogenic/osteogenic markers (DSPP, DMP-1, Runx-2, BMP-2/7, OPN) and mineralization. | Aging pulp cells exhibit chronic inflammation and reduced dentinogenic capacity, which may impair pulp repair and response to injury, affecting outcomes of vital pulp therapy and regenerative endodontics. | *In vitro* model; long-term *in vivo* relevance not tested; specific signaling pathways linking inflammation to dentinogenesis not fully elucidated. |
| Asghari et al., 2021 | *In vitro* | Human dental pulp stem cells (mesenchymal stem cells) | To investigate the effect of high glucose on pulp cell aging and evaluate the role of Wnt/β-catenin signaling as the underlying mechanism. | Long-term exposure to high glucose (20 mM and 30 mM for 7, 14, 21 days) | SA-β-galactosidase activity, p21 gene expression, β-catenin, Wnt1 | High glucose reduced cell proliferation, increased SA-β-gal activity and p21 expression, and upregulated β-catenin and Wnt1. β-catenin inhibitor (PNU-74654) reduced senescence, while inducer (LiCl) enhanced it under high glucose. | Hyperglycemia-induced pulp cell senescence via Wnt/β-catenin may impair pulp healing in diabetic patients. Targeting this pathway could inform tailored endodontic therapies (e.g., pulp capping materials) for diabetic individuals. | *In vitro* study; clinical correlation not established; complex *in vivo* diabetic microenvironment not fully replicated. |
| Dou et al., 2023 | *In vitro* | Human Dental Pulp Stem Cells (DPSCs) | To investigate whether ADM overexpression alleviates DPSC senescence and explore the miR-152/CCNA2 pathway. | Natural passage (up to P15) and H₂O₂ treatment (400 nM). | β-galactosidase (SA-β-gal), p53, p21^WAF1^, p16^INK4A^, ROS, cell cycle (G2/M phase), CCNA2. | ADM overexpression reduced SA-β-gal activity, decreased p53/p21/p16 levels, lowered ROS, increased G2/M phase ratio, and upregulated CCNA2 by inhibiting miR-152. | Suggests ADM as a potential target to maintain DPSC youthfulness, possibly improving stem cell-based regenerative endodontic outcomes. | *In vitro* study; clinical relevance and long-term effects *in vivo* are unknown. |
| Liu et al., 2011 | *In vitro* | Human Dental Pulp Cells (DPCs) from explant culture | To investigate the expression pattern of reprogramming markers (Oct-4, Sox2, c-Myc) in DPCs during long-term culture and its relation to replicative senescence. | Natural long-term *in vitro* passaging (up to P7). | Cell morphology (enlargement, flattening), nuclear/cytoplasmic translocation of Oct-4, Sox2, c-Myc, mRNA expression of these markers. | Reprogramming markers (Oct-4, Sox2, c-Myc) were expressed in early passages (nuclear localization), peaked at P2, and diminished/translocated to cytoplasm by P7, correlating with senescent morphology. | Suggests loss of stemness markers during culture may limit DPC regenerative potential. Optimizing culture conditions to maintain these markers could improve cell-based endodontic therapies. | *In vitro* study using explant culture; direct functional link between marker loss and regenerative capacity not tested. |
| Ghaffari & Shrestha, 2025 | *In vitro* | Human Dental Pulp Stem Cells (DPSCs) | To investigate how substrate stiffness (2D/3D) regulates DPSC quiescence, activation, and senescence via the NF-κB pathway. | Culture on substrates of varying stiffness (2 kPa, 50 kPa) for up to 14 days. | SA-β-gal activity, nuclear size/chromatin condensation, gene expression (p16<sup>INK4A</sup>, SIRT1, BMI-1, pluripotency markers). | Softer substrates (2 kPa) promoted quiescence, reduced senescence, and enhanced stemness markers (OCT4, NANOG, SOX2) via NF-κB activation. Stiffer substrates (50 kPa) increased senescence. | Optimizing scaffold stiffness in bioreactors or delivery systems could preserve DPSC stemness during *ex vivo* expansion, improving outcomes in regenerative endodontics. | *In vitro* study; long-term effects and *in vivo* translation not assessed. 3D scaffold mechanical heterogeneity was noted as a limitation. |
| Yao et al., 2023 | *In vitro* & *Ex vivo* | CD51+/PDGFR-α+ Human Dental Pulp Stromal Cells (hDPSCs) | To compare the distinct effects of chronological (donor age) and replicative (*in vitro* passaging) senescence on CD51+/PDGFR-α+ hDPSCs. | Chronological: Donor age (young vs. aged). Replicative: Long-term *in vitro* passaging (up to p15). | SA-β-gal, p21, p16, Ki67, γ-H2AX, stemness markers (NANOG, OCT4), surface markers (CD51, PDGFR-α, CD90, CD105). | Chronological senescence decreased CD51+/PDGFR-α+ cell number and self-renewal, but osteogenic potential was maintained or increased. Replicative senescence decreased CD51 expression, self-renewal, and osteogenic potential, but increased PDGFR-α. Both types impaired self-renewal. | Highlights the need to consider donor age and limit *in vitro* expansion to maintain hDPSC regenerative capacity for cell-based endodontic therapies. Distinguishing senescence type is crucial for therapeutic strategy. | *In vivo* functional validation of the isolated cell population is needed. Distinction between stem and progenitor cells within the population is unclear. |
| Tang et al., 2025 | *In vitro* | Human Dental Pulp Stem Cells (DPSCs) | To investigate the role of abnormal mitochondrial fusion/fission in inflammatory senescence of DPSCs. | Inflammatory model: E. coli Lipopolysaccharide (LPS). Pharmacological modulation of fusion (OPA1 inhibitor MYLS22) and fission (DRP1 inhibitor Mdivi-1). | SA-β-gal, p53, p21, apoptosis markers, mitochondrial dynamics proteins (OPA1, DRP1), oxidative stress (ROS, MDA, GSH), mitochondrial function (MMP, ATP). | LPS induced inflammatory senescence (increased p21, SA-β-gal, apoptosis; decreased proliferation/differentiation) associated with decreased OPA1 and increased DRP1. Inhibiting fission (Mdivi-1) ameliorated, while inhibiting fusion (MYLS22) exacerbated senescence. | Targeting mitochondrial dynamics (promoting fusion/inhibiting fission) could be a therapeutic strategy to counteract inflammation-induced DPSC senescence, potentially improving vital pulp therapy outcomes. | *In vitro* study; *in vivo* validation of pharmacological modulation in pulpitis models is required. Clinical translatability needs evaluation. |
| Horibe et al. (2014) | *In vitro* & *In vivo* | Mobilized Dental Pulp Stem Cells (MDPSCs) and colony-derived Dental Pulp Stem Cells (DPSCs) from young and aged donors | To determine the influence of donor age on the stem cell properties and regenerative potential of MDPSCs. | Natural aging (comparison of cells from young vs. aged donors); Long-term culture (serial passaging up to 20th passage). | SA-β-gal activity; mRNA expression of p16, p21, IL-1β, IL-6, IL-8, GROz; Telomerase activity; Telomere length. | Aged MDPSCs showed minimal age-related decline in properties vs. aged DPSCs. SA-β-gal and senescence markers increased significantly in aged DPSCs but only slightly in aged MDPSCs during long-term culture. Telomerase activity and telomere length were better maintained in MDPSCs. Regenerative potential of aged MDPSCs was similar to young MDPSCs. | MDPSCs isolated via G-CSF mobilization may provide a superior, age-independent cell source for pulp regeneration and endodontic therapies in aged patients. | *In vivo* models used immunodeficient mice; Clinical translation in humans requires further validation. |
| Nakashima & Iohara (2014) | *In vitro* & *In vivo* | Mobilized Dental Pulp Stem Cells (MDPSCs) from young and aged dogs/humans | To assess the efficacy, safety, and age-independence of MDPSCs for pulp regeneration prior to clinical trials. | Natural aging (comparison of cells/tissues from young vs. aged donors); Long-term culture (serial passaging). | SA-β-gal activity; Expression of p16, p21; Telomerase activity; Karyotype stability. | Aged MDPSCs maintained high proliferative, migratory, and regenerative potential with minimal senescence marker increase vs. aged DPSCs. SA-β-gal and p16/p21 were lower in MDPSCs. Autologous transplantation in aged dogs showed successful but reduced pulp regeneration volume. | MDPSCs are a promising, age-resistant cell source for regenerative endodontics, supporting clinical translation for pulp regeneration even in aged patients. | *In vivo* volume of regenerated pulp was smaller in aged dogs; Clinical efficacy in human aged patients needs further validation. |
| Feng et al. (2014) | *In vitro* | Human Dental Pulp Mesenchymal Stem Cells (DP-MSCs) from five age groups (5-12 to >50 years) | To investigate age-related changes in DP-MSCs and the role of p16INK4A in senescence via DNA damage and stress response. | Natural aging (comparison of cells from different donor age groups). | SA-β-gal activity; Expression of p16INK4A, p21, γ-H2A.X, HSP60; ROS levels; Cell cycle analysis (G0/G1 arrest). | DP-MSCs showed age-dependent senescence: increased SA-β-gal, p16INK4A, p21, γ-H2A.X, ROS; decreased proliferation, differentiation, HSP60; G0/G1 arrest. p16INK4A knockdown reversed senescent features. | Highlights that aging impairs DP-MSC function via p16INK4A, suggesting targeting this pathway could enhance autologous stem cell therapy outcomes in older patients. | *In vitro* study only; No *in vivo* validation of p16INK4A inhibition for pulp regeneration. |
| Zhai et al., 2017 | *In vitro* | Human Dental Follicle Stem Cells (DFSCs) from young and old donors | To establish a cellular senescence model using hydroxyurea (HU) treatment and explore molecular mechanisms of DFSC aging. | Chemical induction with Hydroxyurea (HU, 8 mM for 12-24 hours). | SA-β-gal activity; γH2AX foci (DNA damage); p53, p21, p16 expression; ROS levels; Cell cycle (G0/G1 arrest); Apoptosis (Annexin V/PI). | HU treatment induced premature senescence: increased SA-β-gal, DNA damage, ROS, p53/p21/p16; reduced proliferation/differentiation; G0/G1 arrest. Old donor DFSCs showed higher sensitivity. Key pathways: DNA damage repair (NHEJ/HR), mitochondrial dysfunction, oxidative stress. | Provides a model to study senescence mechanisms in dental stem cells. Understanding senescence pathways may inform strategies to enhance stem cell function for regenerative endodontics in aged patients. | Model uses chemical induction, which may not fully replicate physiological aging; Findings are *in vitro* and require *in vivo* validation. |
| Ok et al., 2021 | *In vitro* | Immortalized human dental pulp cells (hDPCs); also tested HUVEC, mBMDM, hDPSCs | To investigate whether FK866 protects hDPCs from H₂O₂-induced premature senescence | Hydrogen peroxide (H₂O₂) exposure (400 nM for 24h) | SA-β-gal activity; p21 and p53 protein levels; Telomere damage (γH2AX foci co-localized with TRF1); SASP factors (IL-1β, IL-6, IL-8, COX-2, TNF-α mRNA); NF-κB p65 activation | FK866 inhibited H₂O₂-induced SA-β-gal activity, p21/p53 upregulation, telomere damage, ROS production, NADPH consumption, SASP factor expression, and NF-κB activation. | Suggests FK866 as a potential therapeutic agent to protect dental pulp from oxidative stress-induced senescence and inflammation, potentially improving pulp vitality and repair. | *In vitro* study using an immortalized cell line; effects in primary hDPSCs were not clearly established under the conditions used; direct clinical relevance is not yet proven. |
| Hu et al. 2025 | *In vitro* | Dental pulp stem cells (DPSCs) from healthy and mild periodontitis patients | To examine the impact of mild periodontitis and donor age on the biological properties of DPSCs. | Natural aging (comparison of young vs. older donor cells). | SA-β-gal activity, apoptosis rate (flow cytometry), cell morphology, proliferation, surface markers. | Donor age significantly increased senescence (SA-β-gal positivity) and apoptosis, and decreased proliferation. Mild periodontitis did not significantly affect senescence markers or biological properties of DPSCs. | Teeth with mild periodontitis may be suitable sources for DPSC isolation, potentially expanding the donor pool for regenerative endodontic procedures. | Small sample size; only mild periodontitis was investigated; *in vitro* model may not fully represent the *in vivo* microenvironment. |
| Zhou et al. 2024 | *In vitro* | Human dental pulp cells (hDPCs) from healthy wisdom teeth | To explore the role of serine metabolism and PHGDH in replicative senescence of hDPCs. | Replicative senescence induced by serial passaging (P5 vs. P12). | SA-β-gal activity, P21, LMNB1, Ki67, γH2AX, H3K36me3, SAM levels. | Replicative senescence decreased PHGDH expression, SAM, and H3K36me3 levels. PHGDH inhibition phenocopied senescence. Serine supplementation failed to rescue senescence, implicating folate metabolism and histone methylation dysregulation. | Targeting PHGDH or one-carbon metabolism could be a strategy to delay hDPC senescence during *in vitro* expansion, improving cell quality for pulp regeneration therapies. | Study focused on early osteogenic induction; telomere shortening not assessed as a senescence mechanism; findings are from *in vitro* culture. |
| Zhang et al. 2024 | *In vitro* | Human dental pulp cells (hDPCs) from healthy third molars | To investigate the protective effect of Nesfatin-1 against LPS-induced inflammatory response and senescence in hDPCs. | LPS stimulation (bacterial endotoxin). | SA-β-gal activity, telomerase activity, hTERT, TERF2, PAI-1, p16, SIRT1 expression. | Nesfatin-1 attenuated LPS-induced senescence, reduced SA-β-gal positivity, restored telomerase activity and SIRT1 expression, and downregulated PAI-1 and p16. Its protective effect was mediated by SIRT1. | Nesfatin-1 could be a potential therapeutic agent to protect dental pulp from inflammation-induced senescence, relevant for managing pulpitis. | Senescence and telomerase activity assessed at a single time point; lacks *in vivo* validation; experiments limited to cell culture. |
| Ok et al. 2023 | *In vitro* | Immortalized human dental pulp cells (hDPCs) | To explore the role of the visfatin/TLR4 signaling axis in the senescence of hDPCs and the protective effect of TLR4 blockade. | Treatment with exogenous visfatin. | SA-β-gal activity, p21, p53, γH2AX foci (telomere damage), ROS, NADP+/NADPH ratio, inflammatory cytokines (IL-1β, IL-6, IL-8, COX-2, TNF-α). | Visfatin induced hDPC senescence via TLR4. TLR4 blockade (antibodies or inhibitor) reduced senescence markers, oxidative stress, DNA damage, and inflammatory cytokine expression. | Targeting the visfatin/TLR4 axis could be a novel strategy to counteract pulp cell senescence and "inflammaging," potentially treating pulpitis. | Use of an immortalized cell line may not fully replicate primary cell behavior; findings require *in vivo* validation. |
| Sattari et al. 2022 | *In vitro* | Human dental pulp stem cells (hDPSCs) | To investigate the effect of LPS from periodontal pathogenic bacteria (P. gingivalis and E. coli) on the expression of senescence-related genes in hDPSCs. | Stimulation with LPS from P. gingivalis or E. coli. | mRNA and protein expression of TP53 (p53), CDKN1A (p21), CDKN2A (p16), and SIRT1. | LPS from both bacteria significantly upregulated senescence-related genes (TP53, CDKN1A, CDKN2A, SIRT1) in a time-dependent manner. P. gingivalis LPS had the strongest effect on CDKN1A and SIRT1 at early time points, while E. coli LPS had the greatest effect on TP53 and CDKN2A at 48 hours. | Understanding LPS-induced molecular senescence pathways can inform strategies to protect DPSCs from inflammatory damage, potentially improving outcomes in regenerative endodontic procedures. | *In vitro* model; used a single LPS concentration; focused on gene/protein expression without direct functional senescence assays (e.g., SA-β-gal); does not examine downstream functional consequences of senescence. |
| Alaidaroos et al. 2021 | *In vitro* | Human dental pulp stem cells (DPSCs) | To examine whether intrinsic differences in oxidative stress susceptibility and antioxidant profiles contribute to heterogeneity in DPSC senescence and regenerative potential. | Continuous exposure to sub-lethal doses of exogenous H<sub>2</sub>O<sub>2</sub> (0–200 µM) during culture expansion. | Population doublings (PDs), telomere length, SA-β-galactosidase, p53, p16^INK4a^, p21^waf1^, hTERT, oxidative DNA (8-OHdG) and protein (carbonyl) damage. | High proliferative DPSCs resisted H_2_O_2_-induced senescence (>80 PDs) and had elevated SOD2/GSTZ1 expression. Low proliferative DPSCs underwent premature senescence (4–34 PDs) with higher oxidative damage and inferior antioxidant profiles. Differential SOD2 and GSTZ1 expression is linked to senescence resistance. | Suggests that screening for high antioxidant (SOD2/GSTZ1) DPSC subpopulations could yield more robust cells for pulp regeneration therapies, improving clinical outcomes. | High proliferative DPSCs were derived from only one patient donor; results may not fully represent the heterogeneity across all patient samples. The *in vitro* model may not fully replicate the *in vivo* pulp environment. |
| Ma et al. 2009 | *In vitro* | Rat dental pulp stem cells (DPSCs) from juvenile (2-week) and adult (4-month) donors | To assess age-related changes in proliferation and osteogenic differentiation of DPSCs and the influence of the extrinsic microenvironment (conditioned medium). | Comparison of cells from different ages; exposure to juvenile or adult dental pulp cell-conditioned medium (DPC-CM). | Proliferating Cell Nuclear Antigen (PCNA) for proliferation; osteogenic markers (ALP, BSP, OCN). | Juvenile DPSCs proliferated more but had lower osteogenic potential than adult DPSCs. The phenotype (proliferation vs. differentiation) could be reversed by exposure to age-specific DPC-CM (e.g., adult DPC-CM enhanced juvenile DPSC differentiation). | Highlights the importance of donor age and the local microenvironment (stem cell niche) on DPSC function. Strategies to modulate the niche (e.g., with specific conditioned media) could optimize DPSC properties for regenerative endodontics. | *In vivo* relevance of the conditioned medium model is uncertain. Findings are from a rat model and may not directly translate to human DPSCs. The study focuses on osteogenic, not odontogenic, differentiation. |
| Meng et al. 2022 | *In vitro* | Human dental pulp stem cells (hDPSCs) | To investigate the effect of long-term hypoxic (5% O2) culture on the stemness, senescence, and differentiation potential of hDPSCs during *in vitro* expansion. | Long-term culture under normoxic (ambient O2) vs. hypoxic (5% O2) conditions from passage 3 to 6. | β-galactosidase (SA-β-gal) staining; gene expression of p53 and TGF-β. | Long-term hypoxia inhibited the passage-dependent increase in senescence (reduced SA-β-gal+ cells and p53/TGF-β expression) and stemness loss (increased STRO-1 and OCT4). It also suppressed spontaneous and induced osteogenic/adipogenic differentiation. | Hypoxic culture could be a beneficial strategy for large-scale expansion of hDPSCs for clinical use by delaying senescence and maintaining a more primitive, undifferentiated state, potentially improving the quality of cells for regenerative therapies. | Study focused on passages 3-6; effects of longer-term hypoxia or subsequent differentiation after normoxic recovery are unknown. Mechanisms underlying the observed effects were not explored. |
| Luo et al. 2021 | *In vitro* | Human dental pulp stem cells (DPSCs) | To explore the biological effect of m6A RNA methylation, specifically the role of methyltransferase METTL3, in DPSC biology, including cell cycle control, senescence, and apoptosis. | Lentiviral-mediated knockdown of METTL3. | β-galactosidase (SA-β-gal) staining, TUNEL assay, Annexin V/PI staining (apoptosis), p53 phosphorylation, cell cycle analysis (flow cytometry). | METTL3 is highly expressed in immature DPSCs. Its knockdown induced DPSC senescence and apoptosis, and caused S-phase cell cycle arrest. METTL3 regulates the cell cycle via m6A-dependent modification of PLK1 mRNA. | Identifies RNA epigenetics (m6A/METTL3) as a key regulator of DPSC homeostasis. Targeting this pathway could help maintain DPSC stemness and delay senescence, potentially improving outcomes in vital pulp therapy and regeneration. | Findings are from *in vitro* studies; *in vivo* validation is needed. The precise molecular mechanisms downstream of METTL3/PLK1 require further elucidation. The study focuses on one methyltransferase; the role of other m6A regulatory proteins in DPSCs is not explored. |
| Zhang et al., 2026 | *In vitro* | Human dental pulp cells (hDPCs) | To investigate the protective effects and mechanism of Apelin-12 against LPS-induced cellular senescence in hDPCs. | Lipopolysaccharide (LPS) exposure. | APJ, γH2AX, SA-β-Gal activity, p21 (mRNA/protein), acetylated p53 (ac-p53), SIRT1, SIRT6, hTERT, telomerase activity. | Apelin-12 attenuated LPS-induced senescence by restoring SIRT6 expression (but not SIRT1), which led to reduced p21 and ac-p53, decreased SA-β-Gal and γH2AX, and increased hTERT/telomerase activity. SIRT6 knockdown abolished these effects. | Suggests Apelin-12 and the SIRT6 pathway as potential targets for modulating pulp cell senescence and inflammation, relevant for treating pulpitis and maintaining pulp vitality. | Not reported. |
| Cui et al., 2021 | Single-cell RNA sequencing & *In vitro* | Human dental pulp stem cells (hDPSCs) | To characterize the cellular composition shift during monolayer culture and identify a subpopulation (MCAM+ JAG+ PDGFRA-) that maintains fresh cell-like properties and enhanced differentiation. | Physiological aging (comparison of young vs. aged pulp) and long-term *in vitro* culture (passaging). | SA-β-Gal activity; P16 and P21 mentioned in context of replicative senescence. | The MCAM+ JAG+ PDGFRA- subpopulation, located in the perivascular niche, maintains transcriptional similarity to fresh cells, shows enhanced multilineage differentiation, and its proportion remains stable despite aging or caries. However, its proportion gradually decreases during long-term *in vitro* expansion. | Identifies a resilient stem cell subpopulation within the pulp that is resistant to aging and inflammation, which could be targeted for optimizing cell-based pulp regeneration therapies. | The proportion of the MCAM+ JAG+ PDGFRA- subpopulation declines with prolonged *in vitro* culture, which may limit its expansion for clinical use. The study primarily focuses on characterization; functional senescence rescue experiments were not performed. |
| Xing et al., 2019 | *In vitro* | Mesenchymal stem cell-containing cells from gingiva (MSCs/GCs), dental pulp (MSCs/DPCs), and periodontal ligament (MSCs/PDLCs) | To comprehensively compare the biological characteristics (proliferation, senescence, differentiation) of three types of dental MSCs under donor-matched conditions. | Long-term *in vitro* culture (serial passaging to passage 11). | SA-β-Gal staining; P16 and P21 protein levels. | MSCs/GCs showed the highest proliferation and were least susceptible to senescence. MSCs/PDLCs had the best osteogenic and adipogenic potential but were most prone to senescence. All cell types showed decreased stemness and differentiation potential after long-term culture. | Provides guidance for selecting optimal dental MSC sources for regenerative procedures: MSCs/PDLCs are best for bone regeneration but have limited expansion potential due to senescence, whereas MSCs/GCs are more proliferative and senescence-resistant. | Different isolation methods were used for the cell types (outgrowth for PDL, digestion for pulp/gingiva). The study only evaluated cells up to passage 11; effects of further passaging are unknown. Donor sample size was limited (n=5). |
| Lu et al., 2025 | *In vitro* | Supernumerary tooth-derived pulp stem cells (SNTSCs), dental pulp stem cells (DPSCs), stem cells from human exfoliated deciduous teeth (SHED) | To comprehensively compare the biological characteristics (proliferation, migration, differentiation, vitality, senescence) of three types of dental pulp stem cells for evaluating their potential in endodontic regeneration. | Long-term cryopreservation (storage for two years) and serial passaging (evaluated at passage 10). | SA-β-Gal staining; mRNA expression of P16, P21, P53, RBL1. | After two years of storage, DPSCs showed higher SA-β-Gal activity and P16 expression, indicating more senescence, compared to SNTSCs and SHED. SNTSCs and SHED showed similar, lower levels of senescence markers. SNTSCs exhibited superior proliferation and odontogenic potential compared to DPSCs but were inferior to SHED. | SNTSCs, with their favorable proliferation, odontogenic potential, and relative resistance to senescence post-storage, represent a promising alternative cell source for regenerative endodontic procedures. | Donor age differed between groups (SNTSCs/SHED from children, DPSCs from adults), potentially confounding comparisons. The study did not deeply investigate the immunomodulatory capacity of the cells. The specific impact of long-term culture (independent of storage) on senescence was not fully isolated. |
| Wang et al., 2022 | *In vitro* | Human dental pulp stem cells (hDPSCs) | To compare biological characteristics of hDPSCs from different-aged populations and examine the effects of Biodentine on their proliferation and odonto/osteogenic differentiation. | Natural aging (comparison of cells from donors of different age groups: ≤18, 19–59, ≥60 years). | p21 and p16 protein expression; S-phase fraction (cell cycle analysis). | hDPSCs from older donors (≥60 years) showed increased expression of senescence markers (p21, p16), decreased S-phase fraction, prolonged adhesion time, and reduced proliferation and odonto/osteogenic differentiation capacity compared to younger groups. Biodentine promoted proliferation and differentiation in all age groups. | Biodentine may enhance the success of vital pulp therapy (VPT) in elderly patients by promoting the activity of aged hDPSCs, suggesting its potential as a pulp capping material for regenerative endodontic procedures in older populations. | *In vitro* study; clinical efficacy not validated; sample size per age group was small (n=5-6); mechanisms of Biodentine's action on senescence pathways not fully elucidated. |
| Dieterle et al., 2022 | *In vitro* | Human dental pulp stem cells (hDPSCs) | To compare different purification methods for hDPSCs and analyze their impact on biological properties, including senescence. | Natural senescence through *in vitro* culture/passaging; comparison of cells from different purification methods. | Senescence-associated β-galactosidase (SA-β-Gal) activity. | STRO-1+ MACS-enriched (+) hDPSCs exhibited the lowest cellular senescence compared to colony-derived (c) and colony-derived STRO-1+ (c/+) cells. Colony selection methods promoted higher cellular senescence. | Standardized purification (STRO-1+ MACS) yielding low-senescence hDPSCs may improve cell quality and efficacy for pulp regenerative therapies and tissue engineering in endodontics. | *In vitro* study; small donor sample (n=3); findings may not directly translate to *in vivo* clinical outcomes; long-term effects of purification on senescence not fully characterized. |
| Chang et al., 2014 | *In vitro* | Human dental pulp cells (HDPCs) | To establish a Notch signaling-activated HDPC model and investigate its effect on cell senescence. | Serial passaging (replicative senescence); experimental group treated with Jagged1 protein to activate Notch. | Senescence-associated β-galactosidase (SA-β-Gal), p53, p16. | Activation of Notch signaling via Jagged1 delayed senescence phenotypes: reduced SA-β-Gal activity, lower p16 expression (all passages), lower p53 expression (later passages), maintained higher cell viability and ALP activity at later passages compared to controls. | Modulating Notch signaling could be a strategy to delay cellular aging in dental pulp, potentially enhancing the regenerative capacity of pulp cells in vital pulp therapy and improving outcomes in aged patients. | *In vitro* study using a specific Jagged1 activation method; clinical relevance not tested; molecular mechanisms linking Notch to p16/p53 regulation require further study. |
| Dong et al., 2021 | *In vitro* | Human dental pulp stem cells (DPSCs) | To investigate the role of ROR2 in DPSC senescence and the regulatory mechanism involving the STK4-FOXO1/SMS1 axis in sphingomyelin biosynthesis. | Cell passaging (serial cultivation to passage 12). | SA-β-gal activity, p16, p21, p53, SASP genes (MMP3, IL-8, IGFBP-2, GM-CSF). | ROR2 expression decreases in aged pulp/DPSCs. ROR2 downregulation promotes senescence by inhibiting the STK4-FOXO1 axis, reducing SMS1 expression and sphingomyelin biogenesis. Overexpression of ROR2 or SMS1, or SM supplementation, rescues DPSC proliferation and self-renewal. | ROR2 could be a novel therapeutic target to antagonize DPSC aging, potentially enhancing pulp regeneration and the success of endodontic regenerative procedures. | Not reported. |
| Wang et al., 2018 | *In vitro* | SHED and DPSCs | To assess alterations in SHED and DPSC characteristics underlying cellular senescence due to extended *in vitro* amplification. | Serial expansion/long-term *in vitro* culture to passage 20 (P20). | SA-β-gal activity, p53, p21, p16. | Both SHED and DPSCs underwent senescence at P20 (altered morphology, decreased proliferation/migration/differentiation, increased SA-β-gal, apoptosis). SHED exhibited higher proliferation and osteogenic potential than DPSCs at early passage. Senescent DPSCs had higher SA-β-gal+ cells and distinct p53/p21/p16 expression patterns vs. SHED, suggesting different senescence pathways. | SHED may be a more suitable cell source for regenerative endodontic procedures due to better maintenance of stem cell properties during expansion. Selection of appropriate passage number is critical for cell-based therapies. | Not reported. |
| Liao et al., 2019 | *In vitro* | Human odontoblast-like cells (hOBs) and human dental pulp cells (hDPCs) | To investigate the effects of sclerostin on inflammatory responses and cell behavior in LPS-induced inflammatory environment. | Inflammation induced by LPS treatment; study references previous findings that sclerostin accelerates DPC senescence. | Senescence markers not primary focus; study assessed inflammatory cytokines (IL-6, IL-8, IL-1β), adhesion/migration, odontogenic differentiation markers (DSPP, BSP, OPN, OCN, ALP), and angiogenic factors (VEGF, PIGF, VEGFR-1). | LPS upregulated sclerostin in hOBs. Sclerostin enhanced pro-inflammatory cytokine production in hOBs via NF-κB pathway, promoted adhesion/migration/angiogenesis in hDPCs, but inhibited their odontoblastic differentiation. | Sclerostin inhibition could be a therapeutic target to reduce pulpal inflammation and promote regenerative dentinogenesis in vital pulp therapy. | Not reported. |
| Vaseenon et al., 2025 | *In vivo* | Rat dental pulp tissue | To examine the effects of D-galactose-mediated aging and high-fat diet-promoted obesity on senescence and pathologies in rat dental pulp over time. | Systemic administration of D-galactose (150 mg/kg/day) for 4 or 8 weeks; high-fat diet (HF) for induction of obesity. | p21, p16, RAGE, SA-β-gal. | D-gal administration in obese (HF-fed) rats for 8 weeks synergistically increased pulpal senescence markers (p16, RAGE), inflammation, oxidative stress, impaired mitochondrial dynamics/autophagy/mitophagy, and increased apoptosis. HF alone did not induce senescence but promoted inflammation. | Systemic metabolic diseases (obesity, insulin resistance) and aging can exacerbate dental pulp senescence and pathology, potentially compromising pulp vitality and repair. This highlights the need to consider systemic health in endodontic prognosis and regenerative strategies. | Findings are from an animal model; human relevance needs confirmation. Used aggregated pulp tissue, not specific cell types. Mechanisms of ROS production and specific cell vulnerabilities not identified. |
| Mas-Bargues et al., 2023 | *In vitro* | Human dental pulp stem cells (hDPSCs) | To assess the effect of extracellular vesicles (EVs) from senescent hDPSCs (cultured at 21% O2) on young hDPSCs (cultured at 3% O2). | Culture under ambient oxygen tension (21% O2) to induce oxidative stress and premature senescence. | Antioxidant gene expression (MnSOD, CAT, GPx), proliferation, viability, migration, apoptosis, mitochondrial bioenergetics (OCR, membrane potential, ROS). | EVs from senescent hDPSCs induced antioxidant gene expression in young hDPSCs, improved their proliferation, viability, and migration, and reduced apoptosis. Mitochondrial bioenergetics were adapted (reduced maximal/spare respiration) but function was preserved. | Senescence-associated EVs may promote adaptive responses and improve the regenerative capacity (proliferation, survival, migration) of neighboring pulp stem cells, potentially aiding pulp repair. | Not reported. |
| Peng et al., 2025 | *In vitro* & *In vivo* | Human dental pulp stem cells (hDPSCs) | To explore the protective effect and mechanism of melatonin (Mel) on hDPSCs under oxidative stress. | *In vitro*: H₂O₂ treatment. *In vivo*: Streptozotocin (STZ)-induced type 1 diabetes mellitus (T1DM) rat model. | SA-β-gal staining, p16, mitochondrial ROS, membrane potential, NAD+ levels, SIRT1, PARP1, apoptosis markers (caspase-3, BCL2/BAX). | Melatonin alleviates oxidative stress-induced mitochondrial dysfunction and senescence in hDPSCs by ameliorating NAD+ homeostasis via the NAMPT-NAD+-SIRT1 axis. | Melatonin could protect dental pulp stem cells from oxidative stress and senescence, potentially improving survival and function in regenerative endodontic procedures. | Not reported |
| Zhang et al., 2021 | *In vitro* | Human dental pulp stem cells (DPSCs) from young (18-27 yrs) and aging (65-74 yrs) donors | To determine the effects of metformin on DPSC senescence. | Natural aging (comparison of young vs. aging DPSCs); metformin treatment effect on senescence markers. | SA-β-gal staining, p53, p21, p16, miR-34a-3p, CAB39, p-AMPK, p-mTOR. | Metformin alleviates senescence in DPSCs by downregulating miR-34a-3p, upregulating CAB39, and activating the AMPK/mTOR signaling pathway. | Metformin may serve as a pharmacological agent to mitigate DPSC senescence, potentially enhancing the efficacy of stem cell-based regenerative endodontic therapies. | Not reported |
| Malik et al., 2025 | *In vitro* | Dental pulp-derived stem cells (DPSCs) | To evaluate the properties of DPSCs after long-term cryopreservation (up to 13 years). | Senescence assessed post-cryopreservation (no active induction). | SA-β-gal activity, gene expression of p16, p21, p53. | DPSCs maintained high viability, immunophenotype (CD73+/CD90+/CD105+), proliferative capacity, multipotency, and showed no significant senescence (no SA-β-gal+ cells, variable but non-significant gene changes) after up to 13 years of cryopreservation. | Long-term cryopreservation does not compromise DPSC viability or function, supporting the feasibility of DPSC biobanking for future autologous/allogeneic regenerative endodontic therapies. | Small sample size (n=3 per group, pilot study); control group from different donor cohort; assessment limited to early passages; potential donor variability; *in vitro* findings may not fully predict *in vivo* behavior. |
| Diomede et al., 2019 | *In vitro* | Human Dental Pulp Stem Cells (hDPSCs) | To investigate the effect of HEMA on proliferation and autophagy in hDPSCs. | Not reported | Not reported (Study assessed autophagic markers, not senescence markers) | HEMA treatment decreased cell proliferation and induced autophagy, evidenced by increased LC3-II, Beclin1, ERK/pERK and decreased p62. Senescence was not assessed. | Suggests that resin monomer release (e.g., HEMA) from restorative materials may disrupt pulp cell homeostasis via autophagy, potentially affecting pulp vitality and repair. | The study did not investigate cellular senescence; findings are limited to autophagy mechanisms. |
| Ma et al., 2019 | *In vitro* & *In vivo* | Human Dental Pulp Stem Cells (DPSCs), Periodontal Ligament Stem Cells (PDLSCs), Bone Marrow Mesenchymal Stem Cells (BMMSCs), Adipose-derived Stem Cells (ADSCs) | To compare senescence resistance and regenerative properties of different mesenchymal stem cells (MSCs) during aging and under inflammatory conditions. | Replicative senescence (subculture to passage 6) and inflammation-induced senescence (LPS or TNFα stimulation). | SA-β-gal activity, ROS levels, p16, p21, p53 mRNA expression. | DPSCs showed superior resistance to replicative and inflammation-induced senescence, lower ROS, lower expression of senescence markers (p16, p21, p53), and better maintained osteogenic potential compared to BMMSCs, ADSCs, and PDLSCs. | Suggests DPSCs are a superior, senescence-resistant cell source for regenerative endodontic procedures, particularly in inflamed environments, potentially leading to more predictable pulp/dentin regeneration. | Primarily *in vitro* and animal model data; direct translation to human endodontic clinical scenarios needs validation. |
| Sun et al., 2024 | *In vitro* | Human Dental Pulp Stem Cells (hDPSCs) | To investigate the role of lysine demethylase 3A (KDM3A) in hDPSCs aging and its effect on chondrogenic differentiation capacity. | Replicative senescence (subculture to passage 12) and chemical-induced senescence (Etoposide treatment). | SA-β-gal activity, protein levels of KDM3A and SOX9. | KDM3A expression decreased in senescent hDPSCs. Overexpression of KDM3A in aged hDPSCs promoted their chondrogenic differentiation capacity without affecting stemness markers. | Provides insight into epigenetic mechanisms of DPSC aging. Modulating KDM3A could be a strategy to rejuvenate aged DPSCs, potentially enhancing their utility in regenerative therapies, though the direct link to endodontic-specific regeneration (dentin/pulp) is not explored. | Focus is on chondrogenic differentiation, not odontogenic/dentinogenic potential directly relevant to endodontics. Mechanisms were studied *in vitro* only. |
| Feng et al., 2018 | *In vitro* | Human Dental Pulp Stem Cells (DPSCs) | To investigate the mechanism by which repeated LPS stimulation promotes senescence in DPSCs. | Repeated stimulation with Escherichia coli LPS (10 ng/ml) at different frequencies (once, 3 times, 6 times). | SA-β-gal activity, p53 and p21 protein/mRNA expression, nuclear translocation of p65 (NF-κB). | Repeated LPS stimulation induced DPSC senescence, activating the TLR4/MyD88-NF-κB-p53/p21 signaling pathway. Increased SA-β-gal activity, decreased proliferation, and upregulation of p53/p21 were observed. | Chronic inflammation (e.g., from pulpitis) may induce pulp stem cell senescence via TLR4/NF-κB signaling, potentially impairing pulp repair and regeneration capacity, influencing endodontic treatment outcomes. | *In vitro* study; direct *in vivo* relevance in human pulpitis needs validation. Senescence was induced by high-frequency LPS exposure, which may not fully mimic chronic *in vivo* conditions. |
| lezzi et al., 2021 | Observational (miRNA profiling) | Human Dental Pulp Stromal Cells (hDPSCs) and Human Periosteum-Derived Progenitor Cells (hPDPCs) | To compare senescence-related miRNA profiles between hDPSCs and hPDPCs to understand ageing differences based on tissue origin. | Not reported (Cells from middle-aged donors were compared without external senescence induction). | miRNA expression profiles related to p53, mTOR, and MAPK signaling pathways (e.g., miRNAs targeting CDKN1A/p21, CDKN2A/p16). | hDPSCs showed differential expression of 69 miRNAs compared to hPDPCs, with patterns suggesting an earlier activation of senescence pathways (e.g., p53/p16) in hDPSCs. | Provides insight into the molecular basis of DPSC ageing. Suggests DPSCs may have a distinct, possibly accelerated, ageing profile compared to other MSCs, which could impact their long-term efficacy in regenerative endodontic procedures. | Descriptive miRNA profiling without functional validation of the identified miRNAs in senescence. Does not assess traditional senescence markers (e.g., SA-β-gal) or test senescence induction. |
| Kharat et al., 2025 | *In vitro* | Human Dental Pulp Stem Cells (DPSCs) | To examine the effect of cow urine (SR) on cytotoxicity, differentiation, anti-aging (senescence), anti-oxidative stress, and angiogenesis. | Passage-induced senescence (assessed at passages P10, P12, P14). | Senescence-associated β-galactosidase (SA-β-gal). | Treatment with 1% SR delayed cellular senescence in DPSCs, showing fewer SA-β-gal positive cells compared to untreated controls at later passages. | Cow urine may serve as an adjuvant to delay stress-induced senescence in DPSCs, potentially enhancing cell viability and function in regenerative endodontic procedures. | *In vitro* study; findings may not directly translate to clinical outcomes. Use of a single stem cell type (DPSCs). Specific bioactive components in cow urine not identified. |
| Simon et al., 2009 | *In vitro* & *In vivo* | Bovine odontoblasts (early stage/primary vs. late stage/secondary dentinogenesis) | To compare the transcriptomes of odontoblasts at young (primary dentinogenesis) and mature (secondary dentinogenesis) stages to identify differentially regulated genes and key signaling pathways. | Not reported (comparison of naturally occurring maturation stages). | Not specifically reported. General transcriptomic changes related to maturity were assessed (e.g., DMP1, Osteocalcin, SOST). | The odontoblast transcriptome evolves with functional maturity. Mature odontoblasts showed upregulation of markers like DMP1 and Osteocalcin, and downregulation of others like Collagen I and DSPP, suggesting a less active secretory state. | Provides a molecular basis for understanding odontoblast aging and reduced secretory activity during physiological secondary dentinogenesis. This knowledge could inform strategies to reactivate odontoblasts for reparative dentin formation. | Use of bovine model; findings may not directly translate to humans. Senescence was not a primary focus; specific senescence pathways and markers were not assessed. |
| Ghaffari & Shrestha, 2026 | *In vitro* | Human Dental Pulp Stem Cells (hDPSCs) | To investigate how substrate stiffness (2kPa vs. 50kPa) and dimensionality (2D vs. 3D) regulate stemness, senescence, odontogenic differentiation, and NF-κB signaling in DPSCs. | Mechanical priming on substrates of varying stiffness and dimensionality (culture-induced). | SA-β-gal activity; gene expression of P16, SIRT1, XRCC5, BMI-1. | Soft (2kPa) substrates minimized senescence and promoted early odontogenic differentiation. 3D scaffolds led to intermediate, stable senescence levels and late-stage differentiation with increased DNA repair activity (XRCC5). Standard 2D culture plates (CP) induced the highest senescence. | Substrate mechanics are crucial for controlling DPSC senescence and differentiation. Designing regenerative scaffolds with soft, 3D architectures may optimize stem cell function and reduce senescence for improved dentin-pulp regeneration. | *In vitro* study; lack of NF-κB pathway inhibition experiments to confirm causal links; effects of mechanical unloading or substrate switching not assessed. |
| Ning et al., 2020 | *In vitro* & *In vivo* | Rat Dental Pulp Stem Cells (JDPSCs from juveniles, ADPSCs from adults) | To comparatively evaluate changes in proliferation and mineralization abilities of DPSCs from juvenile vs. adult rats in an LPS-induced inflammatory microenvironment. | Age-related (comparison of cells from juvenile vs. adult rats). | Senescence-associated β-galactosidase (SA-β-gal). | ADPSCs exhibited higher SA-β-gal positivity than JDPSCs. Low-concentration LPS promoted proliferation and mineralization in both, but effects were stronger in JDPSCs. Adult rat inflamed pulp showed higher IL-1β and lower OCN expression. | Age-related decline in DPSC function under inflammation suggests younger donor cells may be more effective for regenerative endodontic procedures. The inflammatory microenvironment differentially affects cellular repair capacity with aging. | Use of a rat model; findings may not directly translate to human clinical outcomes. Inflammatory stimulation was limited to LPS; other inflammatory mediators were not explored. |
| Couve & Schmachtenberg, 2011 | Observational (*ex vivo* human tissue) | Human odontoblasts | To characterize the autophagic-lysosomal system in human odontoblasts and analyze its changes with aging. | Natural aging process (comparison of teeth from young, young-adult, and adult patients). | Lipofuscin accumulation; autophagic markers (LC3, LAMP2); acid phosphatase activity. | Autophagic activity (evident by autophagic vacuoles and LC3/LAMP2 expression) is prominent in young odontoblasts but declines with age, accompanied by progressive lipofuscin accumulation within lysosomes. This indicates reduced lysosomal function and autophagic turnover in aged odontoblasts. | The age-related decline in autophagic activity and accumulation of cellular waste (lipofuscin) may compromise odontoblast viability and secretory function, reducing the dentinogenic repair capacity in older teeth. Understanding this process is key for managing age-related differences in pulp repair. | Observational study; causal relationships between autophagic decline and functional loss are inferred. No direct experimental induction or modulation of senescence was performed. Sample size was limited. |
| Saiyasilp et al., 2025 | *In vitro* | Human Dental Pulp Cells (HDPCs) | To investigate the effects of D-galactose on cellular senescence induction, cell proliferation, mineralization, and odontogenic gene expression in HDPCs. | Treatment with D-galactose (1 g/L and 10 g/L) for 48 hours. | SA-β-gal activity, p16 and p21 gene expression. | 10 g/L D-gal significantly reduced cell proliferation, increased SA-β-gal positive cells, and upregulated p16 and p21. Co-incubation with Biodentine (BD) reduced senescence markers but did not improve proliferation or mineralization. | Suggests D-gal can be used to create an *in vitro* aging pulp model; BD may have anti-senescence properties beneficial for vital pulp therapy in aged pulp. | Short-term induction (48-72 hrs); lack of investigation into other aging pathways (e.g., oxidative stress, inflammation); *in vitro* model may not fully replicate *in vivo* aging. |

**Supplementary table 5.** Characteristics of Studies on Senescence in Dental Pulp Cells.
