# Supplementary figures and images for: Cellular senescence in the dental pulp and its implications for endodontics: a scoping review
Source: Clin Oral Investig. 2026 Mar 31;30(4):161. doi: 10.1007/s00784-026-06822-x (PMC13035753; doi:10.1007/s00784-026-06822-x)

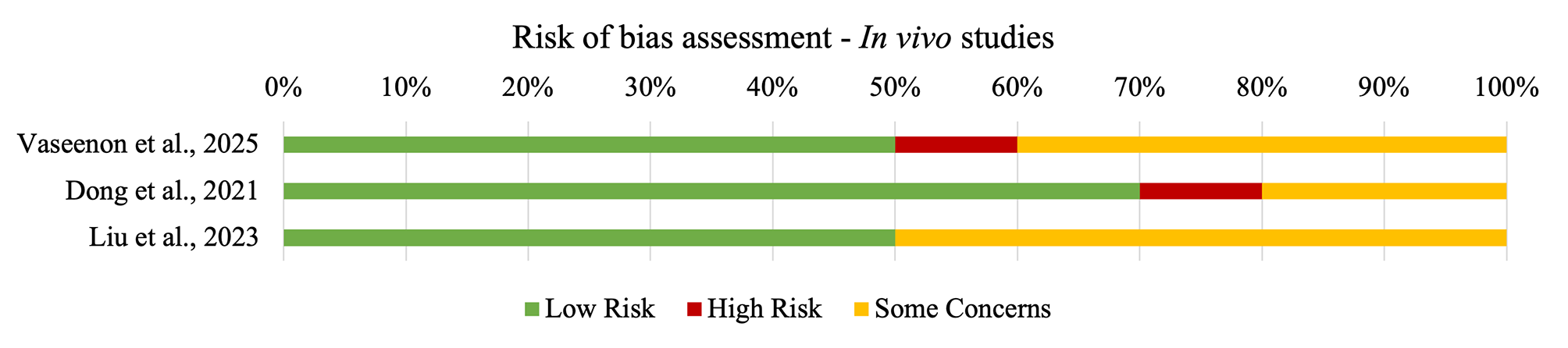

Supplement: Supplementary file 6 — (PNG 70.9 KB) [file 784_2026_6822_Fig4_ESM.png]

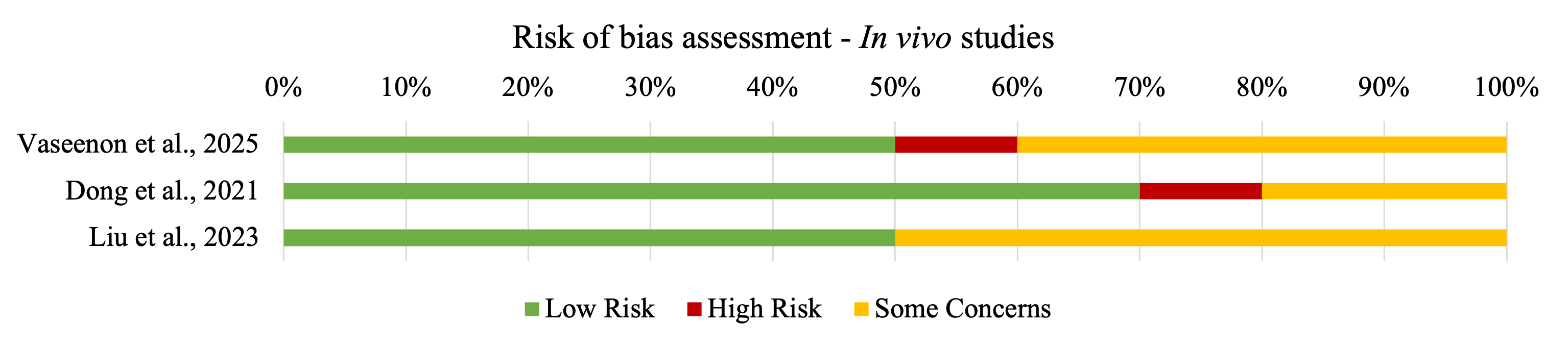

Supplement: Supplementary file 7 — High Resolution Image (TIF 6.38 MB) [file 784_2026_6822_MOESM6_ESM.tif]

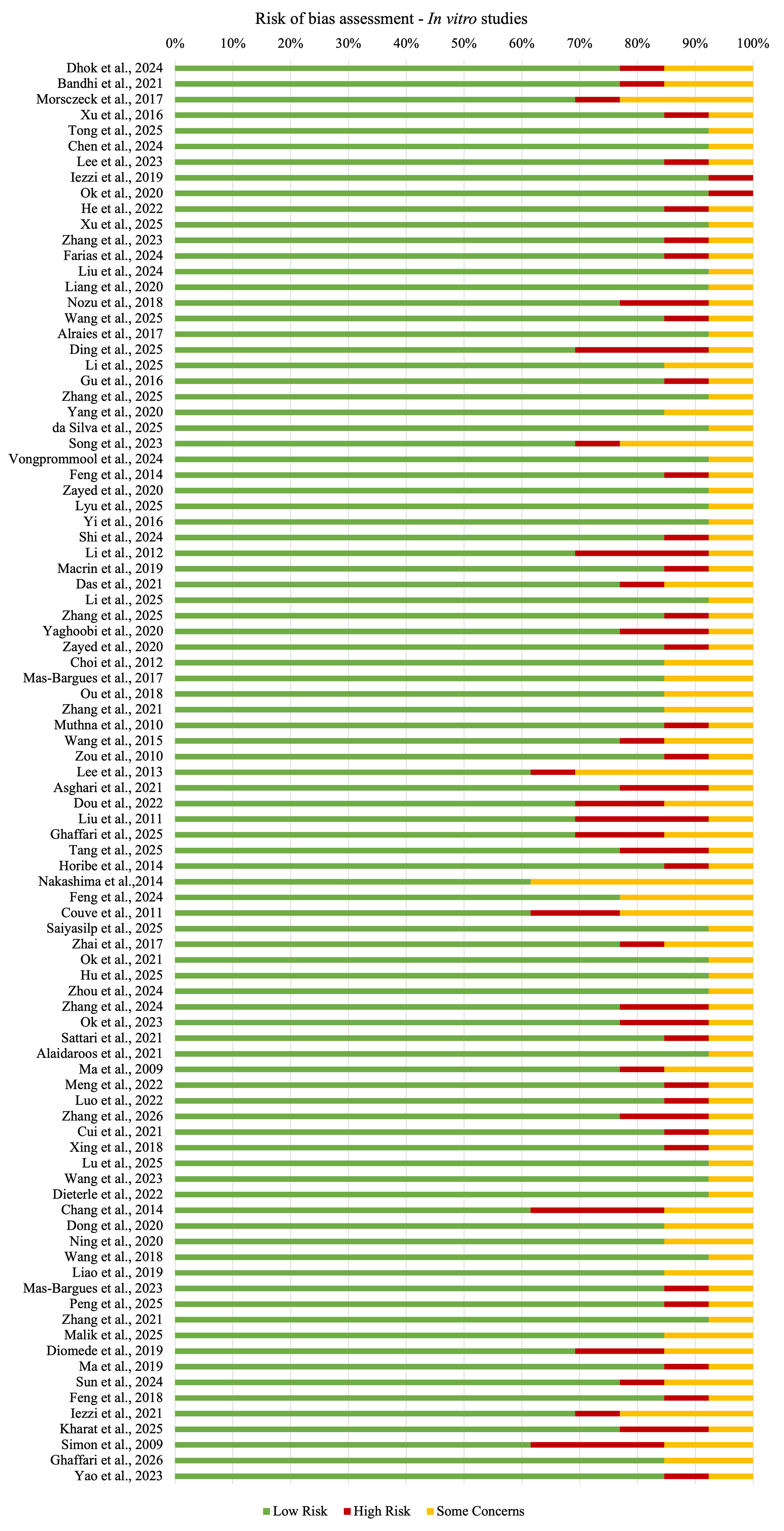

Supplement: Supplementary file 8 — (PNG 144 KB) [file 784_2026_6822_Fig5_ESM.png]

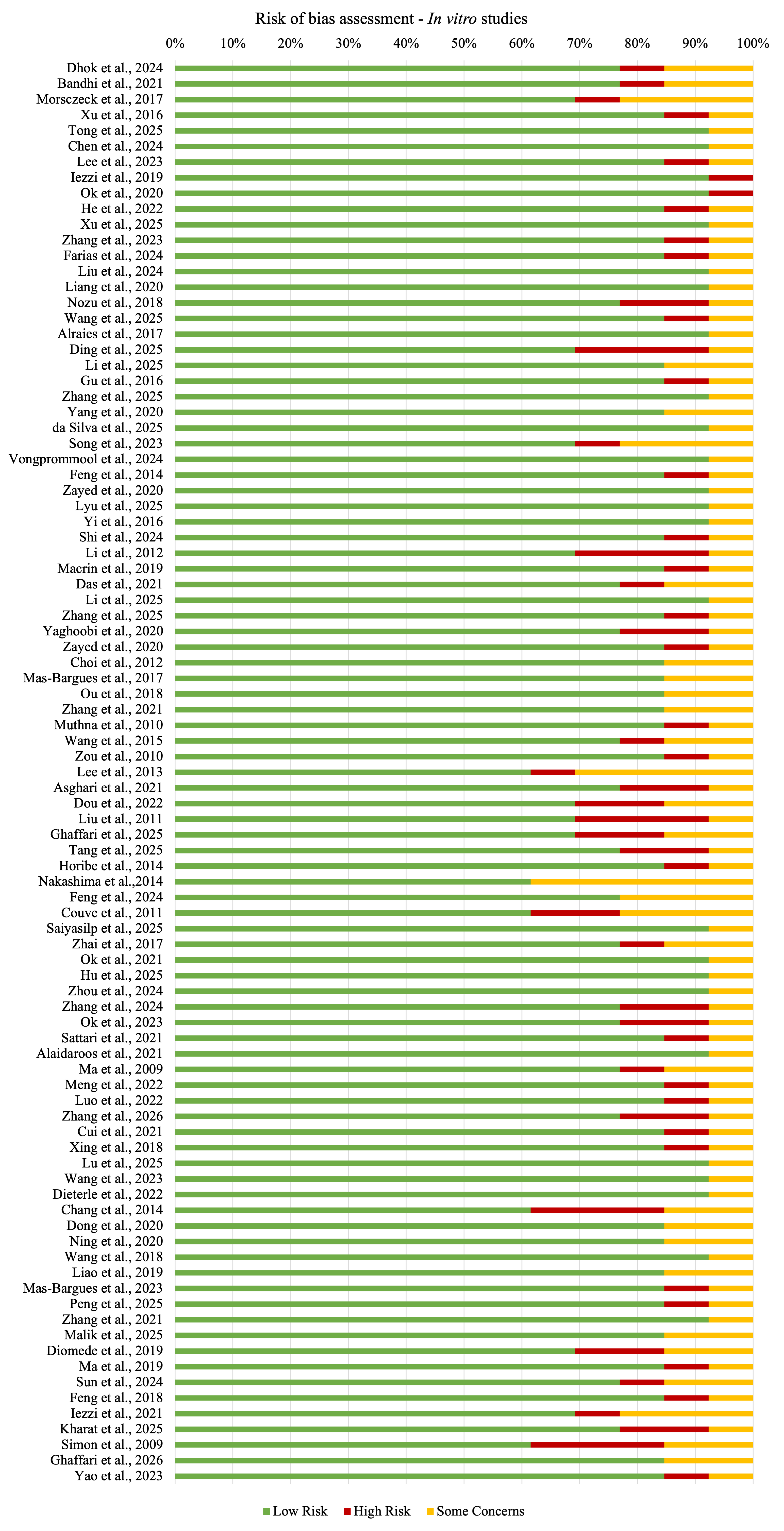

Supplement: Supplementary file 9 — High Resolution Image (TIF 57.6 MB) [file 784_2026_6822_MOESM7_ESM.tif]

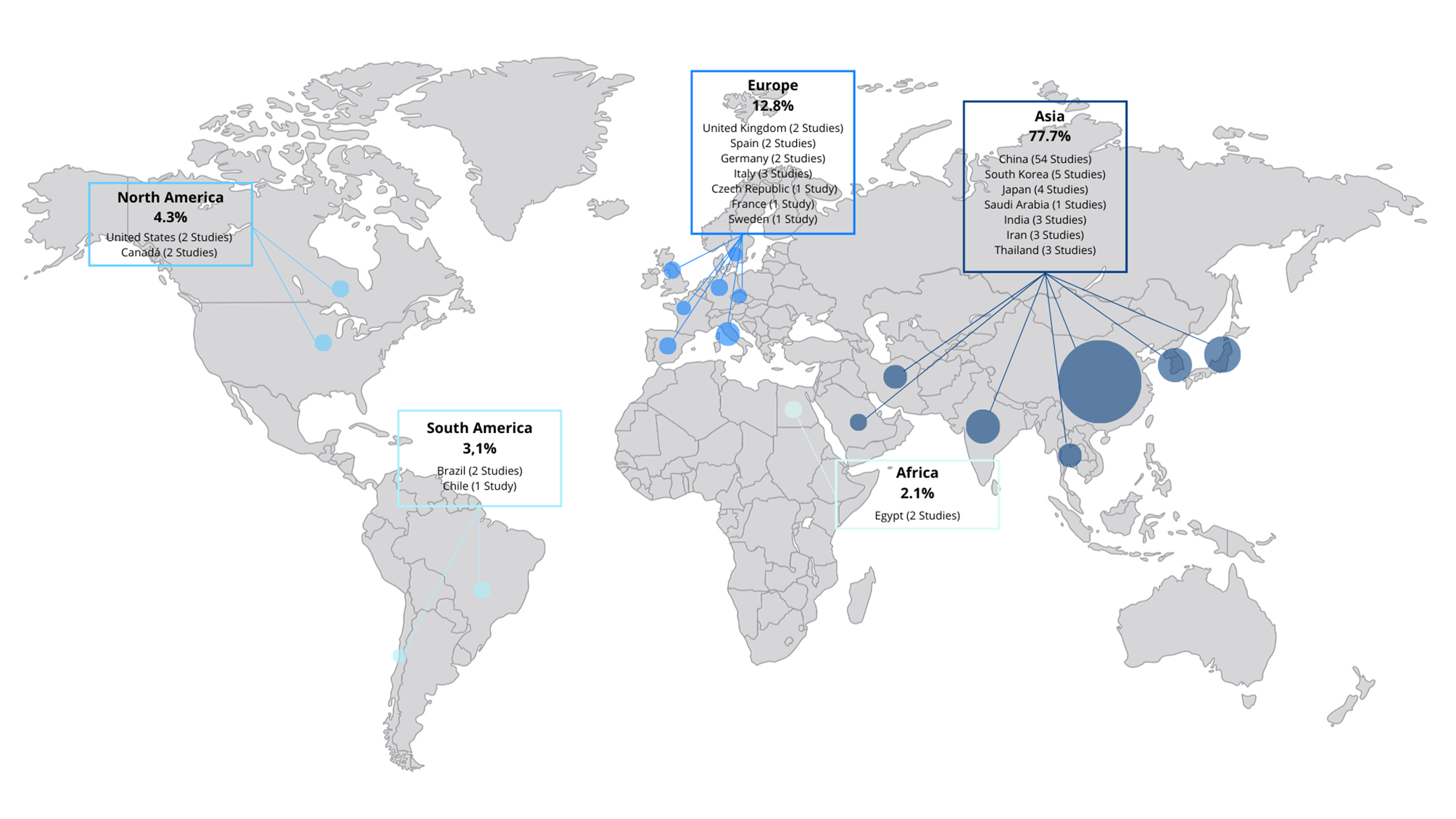

Supplement: Supplementary file 10 — (PNG 585 KB) [file 784_2026_6822_Fig6_ESM.png]

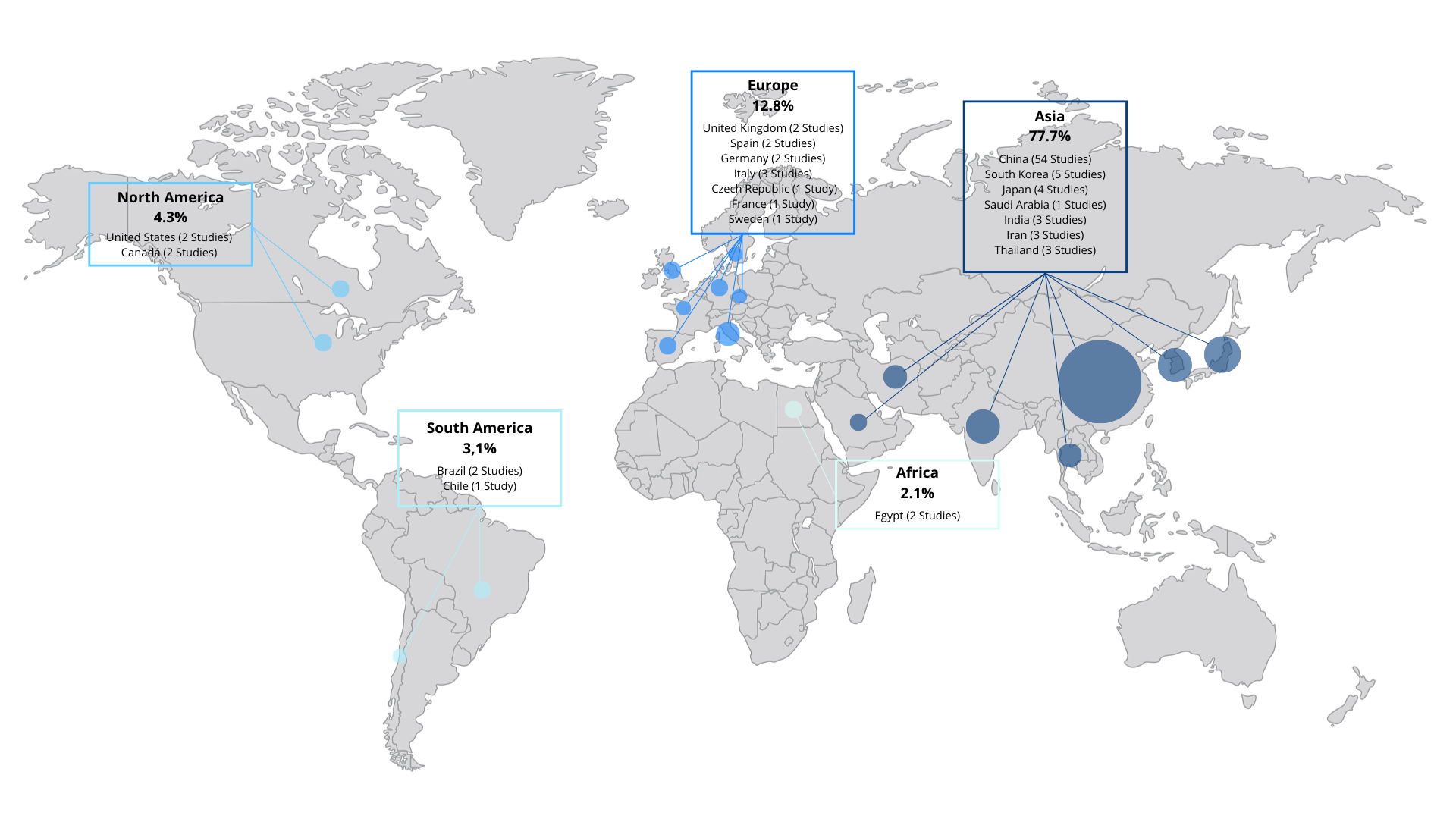

Supplement: Supplementary file 11 — High Resolution Image (TIF 5.95 MB) [file 784_2026_6822_MOESM8_ESM.tif]
